# Supplementary material for: C–O Coupling of Hydrazones with Diacetyliminoxyl Radical Leading to Azo Oxime Ethers—Novel Antifungal Agents
Source: Molecules. 2023 Nov 30;28(23):7863. doi: 10.3390/molecules28237863 (PMC10707749; doi:10.3390/molecules28237863)
Supplement: Supplementary file 1 [file molecules-28-07863-s001.zip › 2023-11-25 Supporting info.pdf]

Supporting Information

C–O Coupling of Hydrazones with Diacetyliminoxyl Radical Leading to Azo Oxime Ethers—Novel Antifungal Agents

Alexander S. Budnikov,[1,2] Igor B. Krylov,[1,2,3*] Mikhail I. Shevchenko,[1,3] O.O. Segida,[1,2] Andrey V. Lastovko,[1] Anna L. Alekseenko,[1,3] Alexey I. Ilovaisky,[1,2] Gennady I. Nikishin,[1] Alexander O. Terent'ev[1,2,3*]

E-mail: krylovigor@yandex.ru; terentev@ioc.ac.ru

[1] N. D. Zelinsky Institute of Organic Chemistry, Russian Academy of Sciences, 47 Leninsky Prospekt, 119991 Moscow, Russia

[2] All-Russian Research Institute for Phytopathology, B. Vyazyomy, 143050 Moscow, Russian

[3] Higher Chemical College of the Russian Academy of Sciences, D. I. Mendeleev University of Chemical Technology of Russia, 9 Miusskaya Square, 125047 Moscow, Russian

Table of contents

General 3
Characterization data of synthesized products 3
X-ray single-crystal diffraction: Structure determination of compound 3ag 13
Computational details 15
References 24
The ^{1}H and ^{13}C spectra of synthesized compounds..26

General

In all experiments RT stands for 22–25 °C. ^{1}H and ^{13}C NMR spectra were recorded on a Bruker AVANCE II 300 and Bruker Fourier 300HD (300.13 for ^{1}H and 75.47 MHz for ^{13}C, respectively) spectrometers in $CDCl_3$. Chemical shifts were reported in parts per million (ppm), and the residual solvent peak was used as an internal reference: ^{1}H ($CDCl_3$ δ = 7.26 ppm), ^{13}C ($CDCl_3$ δ = 77.16 ppm). Multiplicity was indicated as follows: s (singlet), d (doublet), t (triplet), q (quartet), m (multiplet). Coupling constants were reported in Hertz (Hz). FT-IR spectra were recorded on Bruker Alpha instrument. High resolution mass spectra (HR-MS) were measured on a Bruker maXis instrument using electrospray ionization (ESI). The measurements were performed in a positive ion mode (interface capillary voltage – 4500 V); mass range from m/z 50 to m/z 3000 Da; external calibration with Electrospray Calibrant Solution (Fluka). A syringe injection was used for all acetonitrile solutions (flow rate 3 μL/min). Nitrogen was applied as a dry gas; interface temperature was set at 180 °C.

Phenylhydrazine 97%, *p*-tolylhydrazine hydrochloride 98%, 4-chlorophenylhydrazine hydrochloride 97%, 4-(trifluoromethyl)phenylhydrazine 96%, 2-hydrazinopyridine 98%, 2,4-dinitrophenylhydrazine 97%, methylhydrazine 98%, acetophenone 98%, 4-methylacetophenone 95%, 4-nitroacetophenone 98%, 4-methoxyacetophenone 98%, 4-bromoacetophenone 98%, 2-hydroxyacetophenone 98%, propiophenone 99%, benzophenone 99%, benzaldehyde 98%, 4-chlorobenzaldehyde 98%, 4-methoxybenzaldehyde 99%, 4-heptanone 98%, 6-undecanone 97%, 5-hexen-2-one 98%, cyclobutanone 98%, cyclopentanone 99%, cyclohexanone 99%, acetaldehyde 99.5%, propionaldehyde 98%, isobutyraldehyde 99+%, pivaldehyde 96%, hexanal 96%. Hydrazones **2** were synthesized by condensation with corresponding carbonyl compounds.[1–7] Ketones and corresponding hydrazones **2l–n** were synthesized according published procedures.[8–11] CH_2Cl_2 was distilled prior to use. Acetone was distilled over $KMnO_4$. Preparation of diacetyliminoxyl radical is described earlier.[12] To a stirred solution of diacetyl oxime (258 mg, 2 mmol) in 4 mL of CH_2Cl_2 $Pb(OAc)_4$ (469 mg, 1.0 mmol) was added with vigorous stirring. Stirring was continued for 10 min, then the reaction mixture was chromatographed on silica gel using CH_2Cl_2 as eluent. The fraction corresponding to the dark-red spot was collected, so that the volume of the fraction was 50 mL.

Characterization data of synthesized products

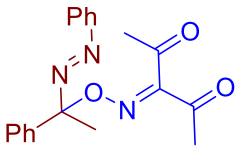

(*E*)-3-((1-phenyl-1-(phenyldiazenyl)ethoxy)imino)pentane-2,4-dione, **3aa** was synthesized as yellow oil (84%, purified by column chromatography with DCM as eluent). **^{1}H NMR** (300.13

MHz, $CDCl_3$): δ = 7.81 – 7.69 (m, 2H), 7.56 – 7.43 (m, 5H), 7.43 – 7.28 (m, 3H), 2.48 (s, 3H), 2.27 (s, 3H), 2.05 (s, 3H). **^{13}C NMR** (75.47 MHz, $CDCl_3$): δ = 198.8, 194.7, 156.6, 151.6, 139.4, 131.6, 129.2, 128.7, 128.6, 126.5, 122.9, 105.2, 30.5, 25.9, 23.8. **FT-IR** (thin layer): ν_{max} = 1725, 1690, 1363, 960, 695. **HR-MS** (ESI): *m/z* = 360.1313, calcd. for $C_{19}H_{19}N_3O_3+Na^+$: 360.1319.

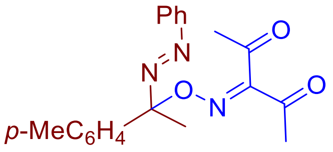

(*E*)-3-((1-(phenyldiazenyl)-1-(p-tolyl)ethoxy)imino)pentane-2,4-dione, **3ab** was synthesized as yellow oil (96%, purified by column chromatography with DCM as eluent). **^{1}H NMR** (300.13 MHz, $CDCl_3$): δ = 7.81 – 7.71 (m, 2H), 7.54 – 7.44 (m, 3H), 7.41 (d, *J* = 8.2 Hz, 2H), 7.19 (d, *J* = 8.2 Hz, 2H), 2.49 (s, 3H), 2.35 (s, 3H), 2.29 (s, 3H), 2.06 (s, 3H). **^{13}C NMR** (75.47 MHz, $CDCl_3$): δ = 198.7, 194.7, 156.5, 151.6, 138.6, 136.4, 131.4, 129.24, 129.18, 126.5, 122.8, 105.2, 30.5, 25.8, 23.5, 21.2. **FT-IR** (thin layer): ν_{max} = 1725, 1687, 1363, 1305, 968. **HR-MS** (ESI): *m/z* = 352.1654, calcd. for $C_{20}H_{21}N_3O_3+H^+$: 352.1656.

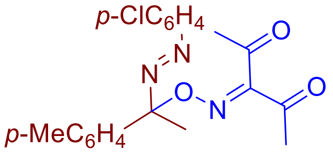

(*E*)-3-((1-((4-chlorophenyl)diazenyl)-1-(p-tolyl)ethoxy)imino)pentane-2,4-dione, **3ac** was synthesized as yellow oil (81%, purified by column chromatography with DCM as eluent). **^{1}H NMR** (300.13 MHz, $CDCl_3$): δ = 7.70 (d, *J* = 8.7 Hz, 2H), 7.44 (d, *J* = 8.7 Hz, 2H), 7.39 (d, *J* = 8.3 Hz, 2H), 7.19 (d, *J* = 8.3 Hz, 2H), 2.47 (s, 3H), 2.35 (s, 3H), 2.29 (s, 3H), 2.04 (s, 3H). **^{13}C NMR** (75.47 MHz, $CDCl_3$): δ = 198.6, 194.6, 156.7, 149.9, 138.7, 137.5, 136.2, 129.4, 129.3, 126.4, 124.1, 105.3, 30.5, 25.8, 23.4, 21.2. **FT-IR** (thin layer): ν_{max} = 1726, 1690, 1362, 1300, 1088, 959. **HR-MS** (ESI): *m/z* = 386.1252, 388.1230, calcd. for $C_{20}H_{20}ClN_3O_3+H^+$: 386.1266, 388.1238.

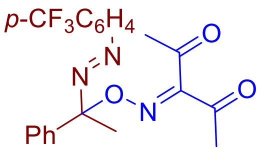

(*E*)-3-((1-phenyl-1-((4-(trifluoromethyl)phenyl)diazenyl)ethoxy)imino)pentane-2,4-dione, **3ad** was synthesized as yellow oil (95%, purified by column chromatography with DCM as eluent). **^{1}H NMR** (300.13 MHz, $CDCl_3$): δ = 7.85 (d, *J* = 8.4 Hz, 2H), 7.75 (d, *J* = 8.4 Hz, 2H), 7.58 – 7.49 (m, 2H), 7.47 – 7.32 (m, 3H), 2.49 (s, 3H), 2.29 (s, 3H), 2.10 (s, 3H). **^{13}C NMR** (75.47 MHz, $CDCl_3$): δ = 198.5, 194.5, 156.7, 153.3, 138.8, 132.89 (q, *J* = 32.7 Hz), 128.9, 128.7, 126.42 (q, *J* = 3.6

Hz), 123.84 (q, J = 272.5 Hz), 123.0, 105.5, 30.5, 25.7, 23.6. **FT-IR** (thin layer): ν_{max} = 1726, 1692, 1364, 1324, 1169, 1131, 1066, 959. **HR-MS** (ESI): m/z = 428.1198, calcd. for $C_{20}H_{18}F_3N_3O_3+Na^+$: 428.1192.

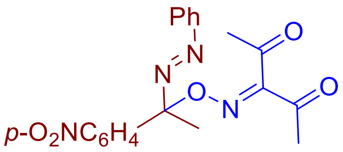

(*E*)-3-((1-(4-nitrophenyl)-1-(phenyldiazenyl)ethoxy)imino)pentane-2,4-dione, **3ae** was synthesized as yellow oil (98%, purified by column chromatography with DCM as eluent). **^{1}H NMR** (300.13 MHz, $CDCl_3$): δ = 8.25 (d, J = 8.9 Hz, 2H), 7.81 – 7.67 (m, 4H), 7.56 – 7.45 (m, 3H), 2.48 (s, 3H), 2.24 (s, 3H), 2.03 (s, 3H). **^{13}C NMR** (75.47 MHz, $CDCl_3$): δ = 198.2, 194.3, 157.1, 151.3, 148.0, 146.6, 132.2, 129.4, 127.8, 123.8, 123.0, 104.1, 30.4, 25.9, 24.4. **FT-IR** (thin layer): ν_{max} = 1727, 1693, 1605, 1522, 1350, 1300, 1142, 1109, 1079, 1067, 958, 855, 769, 758, 693. **HR-MS** (ESI): m/z = 405.1161, calcd. for $C_{19}H_{18}N_4O_5+Na^+$: 405.1169.

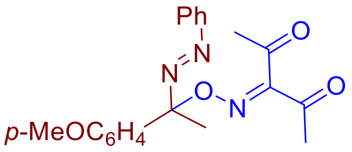

(*E*)-3-((1-(4-methoxyphenyl)-1-(phenyldiazenyl)ethoxy)imino)pentane-2,4-dione, **3af** was synthesized as yellow oil (75%, purified by column chromatography with DCM as eluent). **^{1}H NMR** (300.13 MHz, $CDCl_3$): δ = 7.80–7.67 (m, 2H), 7.55–7.38 (m, 5H), 6.95–6.82 (m, 2H), 3.80 (s, 3H), 2.47 (s, 3H), 2.28 (s, 3H), 2.04 (s, 3H). **^{13}C NMR** (75.47 MHz, $CDCl_3$): δ = 198.8, 194.7, 159.9, 156.5, 151.6, 131.4, 129.2, 128.0, 122.8, 113.9, 105.1, 55.4, 30.5, 25.8, 23.3. **FT-IR** (thin layer): ν_{max} = 1725, 1690, 1608, 1514, 1363, 1303, 1253, 1185, 1109, 1030, 960, 834, 769. **HR-MS** (ESI): m/z = 390.1423, calcd. for $C_{20}H_{21}N_3O_4+Na^+$: 390.1424.

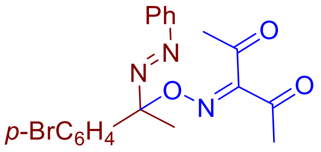

(*E*)-3-((1-(4-bromophenyl)-1-(phenyldiazenyl)ethoxy)imino)pentane-2,4-dione, **3ag** was synthesized as yellow crystals (82%, purified by column chromatography with DCM as eluent). Mp = 90–91 °C. **^{1}H NMR** (300.13 MHz, $CDCl_3$): δ = 7.81 – 7.70 (m, 2H), 7.55 – 7.45 (m, 5H), 7.44–7.35 (m, 2H), 2.47 (s, 3H), 2.27 (s, 3H), 2.01 (s, 3H). **^{13}C NMR** (75.47 MHz, $CDCl_3$): δ = 198.6, 194.6, 156.8, 151.4, 138.6, 131.8, 129.3, 128.4, 123.1, 122.9, 104.6, 102.8, 30.5, 25.9, 23.8. **FT-IR** (thin layer): ν_{max} = 1773, 1484, 1397, 1362, 1302, 1135, 1078, 1010, 966, 920, 828, 685, 550. **HR-MS** (ESI): m/z = 416.0608, 418.0592, calcd. for $C_{19}H_{18}BrN_3O_3+H^+$: 416.0604, 418.0585. Single crystal X-Ray analysis is available (see Fig. 1, page S15).

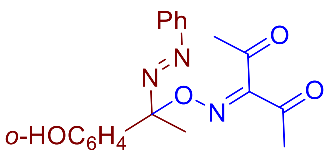

(*E*)-3-((1-(2-hydroxyphenyl)-1-(phenyldiazenyl)ethoxy)imino)pentane-2,4-dione, **3ah** was synthesized as pale yellow solid (74%, purified by column chromatography with DCM as eluent) Mp = 103–104 °C. **^{1}H NMR** (300.13 MHz, $CDCl_3$): δ = 8.16 (s, 1H), 7.74–7.71 (m, 2H), 7.54–7.49 (m, 3H), 7.36–7.27 (m, 2H), 6.97–6.90 (m, 2H), 2.46 (s, 3H), 2.25 (s, 3H), 2.09 (s, 3H). **^{13}C NMR** (75.47 MHz, $CDCl_3$): δ = 198.2, 194.4, 157.0, 155.4, 150.9, 132.5, 131.3, 129.6, 127.3, 124.0, 123.0, 120.3, 118.5, 106.9, 30.6, 25.9, 22.8. **FT-IR** (thin layer): ν_{max} = 1727, 1692, 1483, 1458, 1364, 1299, 1246, 1201, 1105, 957, 939, 76. **HR-MS** (ESI): *m/z* = 376.1260, cald. For $C_{19}H_{19}N_3O_4$+Na^+ = 376.1268.

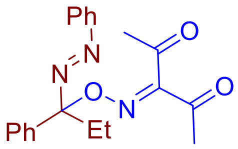

(*E*)-3-((1-phenyl-1-(phenyldiazenyl)propoxy)imino)pentane-2,4-dione, **3ai** was synthesized as yellow oil (87%, purified by column chromatography with DCM as eluent). **^{1}H NMR** (300.13 MHz, $CDCl_3$): δ = 7.85–7.69 (m, 2H), 7.57–7.53 (m, 2H), 7.51–7.47 (m, 3H), 7.44–7.30 (m, 3H), 2.52 (s, 3H), 2.60 – 2.35 (m, 2H), 2.23 (s, 3H), 0.88 (t, *J* = 7.4 Hz, 3H). **^{13}C NMR** (75.47 MHz, $CDCl_3$): δ = 198.8, 194.7, 156.7, 151.6, 138.2, 131.4, 129.2, 128.5, 128.3, 126.9, 122.8, 106.9, 31.1, 30.3, 25.8, 7.7. **FT-IR** (thin layer): ν_{max} =2979, 1726, 1691, 1450, 1363, 1296, 1138, 1070, 963, 763, 699, 691. **HR-MS** (ESI): *m/z* = 374.1472, calcd. for $C_{20}H_{21}N_3O_3$+Na^+: 374.1475.

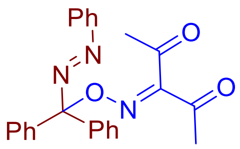

(*E*)-3-((diphenyl(phenyldiazenyl)methoxy)imino)pentane-2,4-dione, **3aj** was synthesized as slightly yellow solid (98%, purified by column chromatography with DCM as eluent). Mp = 103-104 °C. **^{1}H NMR** (300.13 MHz, $CDCl_3$): δ = 7.86–7.75 (m, 2H), 7.58–7.45 (m, 7H), 7.43–7.30 (m, 6H), 2.56 (s, 3H), 2.05 (s, 3H). **^{13}C NMR** (75.47 MHz, $CDCl_3$): δ = 198.7, 194.7, 156.3, 151.5, 139.9, 131.6, 129.3, 128.6, 128.4, 128.0, 123.0, 105.6, 30.1, 25.7. **FT-IR** (thin layer): ν_{max} = 1725, 1686, 1300, 1013, 976, 941, 762, 695. **HR-MS** (ESI): *m/z* = 422.1461, calcd. for $C_{24}H_{21}N_3O_3$+Na^+: 422.1475.

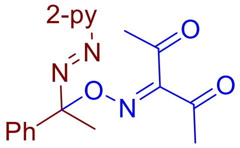

(*E*)-3-((1-phenyl-1-(pyridin-2-yldiazenyl)ethoxy)imino)pentane-2,4-dione, **3ak** was synthesized as yellow oil (89%, purified by column chromatography with PE/EtOAc = 2/5 as eluent). **^{1}H NMR** (300.13 MHz, $CDCl_3$): δ = 8.70 (d, *J* = 4.2 Hz, 1H), 7.85 (td, *J* = 7.7, 1.8 Hz, 1H), 7.60 – 7.48 (m, 3H), 7.46 – 7.29 (m, 4H), 2.48 (s, 3H), 2.28 (s, 3H), 2.12 (s, 3H). **^{13}C NMR** (75.47 MHz, $CDCl_3$): δ = 198.6, 194.6, 162.2, 156.8, 149.6, 138.6, 138.5, 129.0, 128.7, 126.5, 125.8, 114.3, 106.0, 30.6, 25.9, 23.5. **FT-IR** (thin layer): ν_{max} = 1725, 1690, 1583, 1455, 1425, 1363, 1299, 1261, 1194, 1145, 1119,1069, 955, 791, 770, 699. **HR-MS** (ESI): *m/z* = 339.1448, calcd. for $C_{18}H_{18}N_4O_3+H^+$: 339.1452.

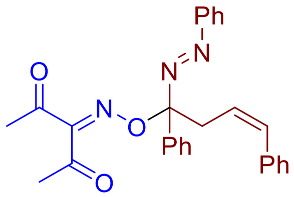

3-((((*Z*)-1,4-diphenyl-1-((*E*)-phenyldiazenyl)but-3-en-1-yl)oxy)imino)pentane-2,4-dione, **3al** was synthesized as slightly yellow viscous gum (87%, purified by column chromatography with DCM as eluent). **^{1}H NMR** (300.13 MHz, $CDCl_3$): δ = 7.79–7.70 (m, 2H), 7.58–7.45 (m, 5H), 7.45–7.17 (m, 8H), 6.54 (d, *J* = 11.8 Hz, 1H), 5.58 (dt, *J* = 11.8, 7.2 Hz, 1H), 3.57 (dd, *J* = 7.2, 1.8 Hz, 2H), 2.50 (s, 3H), 1.98 (s, 3H). **^{13}C NMR** (75.47 MHz, $CDCl_3$): δ = 198.6, 194.7, 156.6, 151.5, 137.3, 137.2, 132.5, 131.6, 129.2, 128.74, 128.67, 128.61, 128.4, 127.0, 126.9, 124.9, 122.9, 106.3, 36.2, 30.3, 25.6. **FT-IR** (thin layer): ν_{max} = 1725, 1690, 1600, 1494, 1449, 1363, 1301, 1193, 1059, 1018, 1003, 950, 765, 699. **HR-MS** (ESI): *m/z* = 462.1781, calcd. for $C_{27}H_{25}N_3O_3+Na^+$: 462.1788.

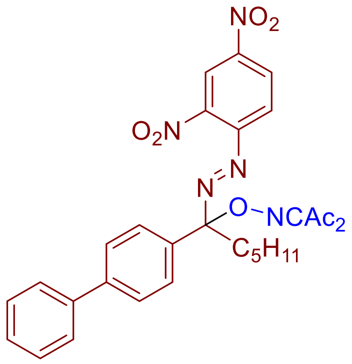

(*E*)-3-(((1-([1,1'-biphenyl]-4-yl)-1-((2,4-dinitrophenyl)diazenyl)hexyl)oxy)imino)pentane-2,4-dione, **3am** was synthesized as viscous orange gum (46%, purified by column chromatography with DCM as eluent). **^{1}H NMR** (300.13 MHz, $CDCl_3$): δ = 8.83 (d, *J* = 2.3 Hz, 1H),

8.51 (dd, *J* = 8.7, 2.3 Hz, 1H), 7.73 – 7.56 (m, 6H), 7.52–7.41 (m, 3H), 7.41–7.32 (m, 1H), 2.60–2.47 (m, 2H), 2.44 (s, 3H), 2.33 (s, 3H), 1.42–1.20 (m, 6H), 0.94 – 0.77 (m, 3H). **^{13}C NMR** (75.47 MHz, $CDCl_3$): δ = 198.5, 194.4, 157.1, 148.6, 148.1, 146.1, 141.7, 140.2, 135.6, 129.0, 128.4, 127.9, 127.5, 127.18, 127.15, 120.5, 120.3, 108.1, 37.3, 31.8, 30.2, 25.8, 22.6, 22.4, 14.0. **FT-IR** (thin layer): ν_{max} = 3103, 2957, 2931, 2869, 1726, 1692, 1608, 1536, 1487, 1346, 1298, 1147, 954, 836, 766, 744, 698. **HR-MS** (ESI): *m/z* = 582.1955, calcd. for $C_{29}H_{29}N_5O_7+Na^+$: 582.1959.

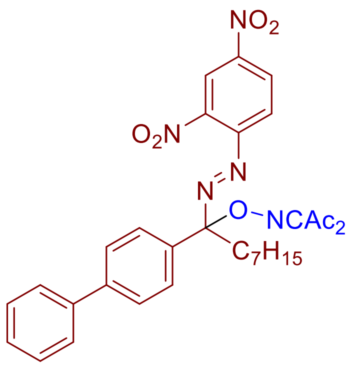

(*E*)-3-(((1-([1,1'-biphenyl]-4-yl)-1-((2,4-dinitrophenyl)diazenyl)octyl)oxy)imino)pentane-2,4-dione, **3an** was synthesized as viscous orange gum (42%, purified by column chromatography with DCM as eluent). **^{1}H NMR** (300.13 MHz, $CDCl_3$): δ = 8.82 (d, *J* = 2.3 Hz, 1H), 8.51 (dd, *J* = 8.7, 2.3 Hz, 1H), 7.78–7.55 (m, 6H), 7.54–7.40 (m, 3H), 7.39–7.29 (m, 1H), 2.66 – 2.49 (m, 2H), 2.45 (s, 3H), 2.34 (s, 3H), 1.55 – 1.09 (m, 10H), 0.86 (t, *J* = 6.5 Hz, 3H). **^{13}C NMR** (75.47 MHz, $CDCl_3$): δ = 198.5, 194.4, 157.1, 148.6, 148.1, 146.1, 141.8, 140.2, 135.6, 129.0, 128.4, 127.9, 127.5, 127.2, 120.5, 120.3, 108.1, 37.4, 31.8, 30.3, 29.6, 29.1, 25.8, 23.0, 22.7, 14.2. **FT-IR** (thin layer): ν_{max} = 1724, 1691, 1607, 1545, 1541, 1346, 1297, 1194, 1146, 963, 835, 766, 747, 698. **HR-MS** (ESI): *m/z* = 605.2712, calcd. for $C_{31}H_{33}N_5O_7+H^+$: 605.2718.

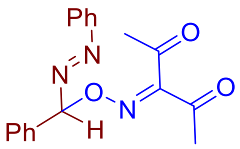

(*E*)-3-((phenyl(phenyldiazenyl)methoxy)imino)pentane-2,4-dione, **3ba** was synthesized as yellow oil (79%, purified by column chromatography with DCM as eluent). **^{1}H NMR** (300.13 MHz, $CDCl_3$): δ = 7.79–7.76 (m, 2H), 7.55–7.51 (m, 2H), 7.50–7.47 (m, 3H), 7.44–7.39 (m, 3H), 6.50 (s, 1H), 2.46 (s, 3H), 2.35 (s, 3H). **^{13}C NMR** (75.47 MHz, $CDCl_3$): δ = 198.9, 194.4, 157.2, 151.6, 134.4, 131.9, 130.7, 129.8, 129.3, 129.0, 127.9, 123.7, 107.3, 30.6, 25.9. **FT-IR** (thin layer): ν_{max} =1725, 1693, 1453, 1419, 1360, 1195, 1098, 1019, 952, 766, 695. **HR-MS** (ESI): *m/z* = 346.1162 cald. For $C_{18}H_{17}N_3O_3+Na^+$ = 346.1162.

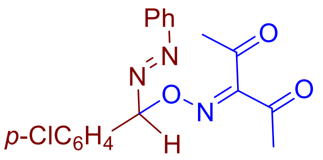

(*E*)-3-(((4-chlorophenyl)(phenyldiazenyl)methoxy)imino)pentane-2,4-dione, **3bb** was synthesized as yellow powder (58%, purified by column chromatography with DCM as eluent). Mp = 49–50 °C. **^{1}H NMR** (300.13 MHz, $CDCl_3$): δ = 7.84–7.69 (m, 2H), 7.57–7.42 (m, 5H), 7.42–7.35 (m, 2H), 6.45 (s, 1H), 2.45 (s, 3H), 2.35 (s, 3H) **^{13}C NMR** (75.47 MHz, $CDCl_3$): δ = 197.7, 194.3, 157.3, 151.4, 135.9, 132.9, 132.1, 129.3, 129.23, 129.19, 123.1, 106.5, 30.6, 26.0. **FT-IR** (thin layer): ν_{max} = 1725, 1697, 1488, 1413, 1363, 1296, 1091, 1049, 1019, 939, 821, 768, 691. **HR-MS** (ESI): *m/z* = 380.0770, cald. for $C_{18}H_{16}ClN_3O_3$+Na^+: 380.0772.

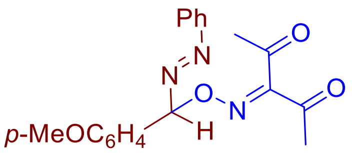

(*E*)-3-(((4-methoxyphenyl)(phenyldiazenyl)methoxy)imino)pentane-2,4-dione, **3bc** was synthesized as yellow solid (88%, purified by column chromatography with DCM as eluent). Mp = 69–70 °C **^{1}H NMR** (300 MHz, $CDCl_3$): δ = 7.81–7.69 (m, 2H), 7.51–7.38 (m, 5H), 6.98 – 6.89 (m, 2H), 6.44 (s, 1H), 3.81 (s, 3H), 2.45 (s, 3H), 2.35 (s, 3H). **^{13}C NMR** (76 MHz, CDCl3): δ = 198.0, 194.5, 160.8, 157.0, 151.5, 131.8, 129.3, 129.2, 126.6, 123.0, 114.4, 107.2, 55.4, 30.6, 25.9. **FT-IR** (thin layer): ν_{max} = 1725, 1692, 1515, 1360, 1300, 1253, 1027, 951. **HR-MS** (ESI): *m/z* = 376.1261 cald. For $C_{19}H_{19}N_3O_4$+Na^+ = 376.1268.

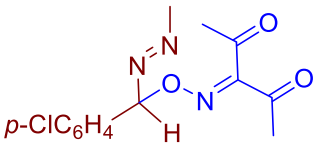

(*E*)-3-(((4-chlorophenyl)(methyldiazenyl)methoxy)imino)pentane-2,4-dione, **3bd** was synthesized as yellow oil (68%, purified by column chromatography with DCM as eluent). **^{1}H NMR** (300.13 MHz, DMSO-d_6): δ = 7.53 (d, *J* = 8.6 Hz, 2H), 7.47 (d, *J* = 8.6 Hz, 1H), 6.36 (s, 1H), 3.85 (s, 3H), 2.35 (s, 3H), 2.32 (s, 3H). **^{13}C NMR** (75.47 MHz, DMSO-d_6): δ = 198.2, 193.7, 156.9, 134.5, 133.1, 129.5, 128.9, 104.5, 57.0, 30.1, 25.6. **FT-IR** (thin layer): ν_{max} = 1727, 1693, 1493, 1363, 1298, 1090, 977, 950. **HR-MS** (ESI): *m/z* = 318.0611, cald. for $C_{13}H_{14}ClN_3O_3$+Na^+: 318.0616.

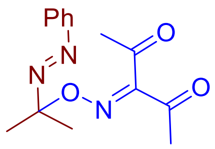

(*E*)-3-(((2-(phenyldiazenyl)propan-2-yl)oxy)imino)pentane-2,4-dione, **3ca** was synthesized as yellow oil (85%, purified by column chromatography with DCM as eluent). **^{1}H NMR** (300.13 MHz, $CDCl_3$): δ = 7.76–7.67 (m, 2H), 7.52 – 7.43 (m, 3H), 2.44 (s, 3H), 2.35 (s, 3H), 1.62 (s, 6H). **^{13}C NMR** (75.47 MHz, $CDCl_3$): δ = 198.9, 194.8, 156.3, 151.6, 131.3, 129.2, 122.6, 104.7, 30.6, 25.8, 23.5. **FT-IR** (thin layer): ν_{max} =1726, 1690, 1384, 1303, 1196, 1173, 1145, 1070, 963, 767, 691. **HR-MS** (ESI): *m/z* = 298.1160, calcd. for $C_{14}H_{17}N_3O_3$+Na^+: 298.1162.

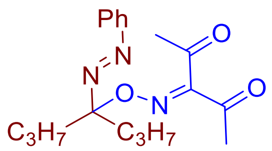

(*E*)-3-(((4-(phenyldiazenyl)heptan-4-yl)oxy)imino)pentane-2,4-dione, **3cb** was synthesized as yellow oil (73%, purified by column chromatography with DCM as eluent). **^{1}H NMR** (300.13 MHz, $CDCl_3$): δ = 7.75–7.63 (m, 2H), 7.54–7.41 (m, 3H), 2.44 (s, 3H), 2.34 (s, 3H), 2.15–1.91 (m, 4H), 1.55 – 1.19 (m, 4H), 0.90 (t, *J* = 7.3 Hz, 6H). **^{13}C NMR** (75.47 MHz, $CDCl_3$): δ = 199.0, 194.8, 156.2, 151.6, 131.2, 129.2, 122.5, 107.5, 37.2, 30.4, 25.8, 16.3, 14.6. **FT-IR** (thin layer): ν_{max} = 2964, 2934, 2875, 1726, 1690, 1363, 1303, 960, 768, 691. **HR-MS** (ESI): *m/z* = 354.1782, calcd. for $C_{18}H_{25}N_3O_3$+Na^+: 354.1788.

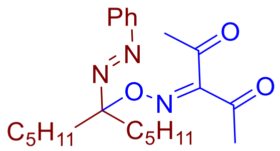

(*E*)-3-(((6-(phenyldiazenyl)undecan-6-yl)oxy)imino)pentane-2,4-dione, **3cc** was synthesized as yellow oil (70%, purified by column chromatography with DCM as eluent). **^{1}H NMR** (300.13 MHz, $CDCl_3$): δ = 7.73–7.64 (m, 2H), 7.53–7.42 (m, 3H), 2.44 (s, 3H), 2.34 (s, 3H), 2.13–1.93 (m, 4H), 1.49–1.18 (m, 12H), 0.86 (t, *J* = 6.8 Hz, 6H). **^{13}C NMR** (75.47 MHz, $CDCl_3$): δ = 198.9, 194.8, 156.3, 151.7, 131.1, 129.2, 122.5, 107.6, 34.8, 32.2, 30.4, 25.8, 22.5, 22.4, 14.1. **FT-IR** (thin layer): ν_{max} = 2957, 2932, 2870, 1727, 1692, 1363, 1301, 960, 767. **HR-MS** (ESI): *m/z* = 410.2402, calcd. for $C_{22}H_{33}N_3O_3$+Na^+: 410.2414.

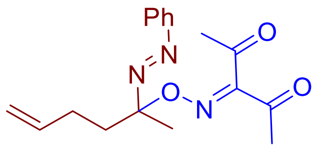

(E)-3-(((2-(phenyldiazenyl)hex-5-en-2-yl)oxy)imino)pentane-2,4-dione, **3cd** was synthesized as slightly yellow viscous gum (79%, purified by column chromatography with PE/EA

= 10/1 as eluent). ^{1}**H NMR** (300.13 MHz, $CDCl_3$): δ = 7.76–7.66 (m, 2H), 7.54–7.44 (m, 3H), 5.93–5.62 (m, 1H), 5.21–4.77 (m, 2H), 2.45 (s, 3H), 2.35 (s, 3H), 2.27 – 1.98 (m, 4H), 1.63 (s, 3H). ^{13}**C NMR** (75.47 MHz, $CDCl_3$): δ = 198.9, 194.8, 156.4, 151.6, 137.7, 131.4, 129.2, 122.6, 115.1, 106.0, 36.4, 30.5, 27.4, 25.9, 21.4. **FT-IR** (thin layer): ν_{max} = 1726, 1690, 1420, 1367, 1303, 982, 960, 826. **HR-MS** (ESI): *m/z* = 338.1475, calcd. for $C_{17}H_{21}N_3O_3$+Na^+: 338.1475.

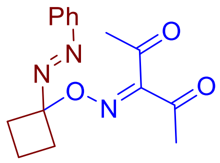

(E)-3-((1-(phenyldiazenyl)cyclobutoxy)imino)pentane-2,4-dione, **3ce** was synthesized as slightly yellow viscous gum (89%, purified by column chromatography with DCM as eluent). ^{1}**H NMR** (300.13 MHz, $CDCl_3$): δ = 7.81–7.70 (m, 2H), 7.56–7.42 (m, 3H), 2.69–2.50 (m, 4H), 2.46 (s, 3H), 2.35 (s, 3H), 2.09–1.84 (m, 2H).^{13}**C NMR** (75.47 MHz, $CDCl_3$): δ = 198.7, 194.7, 157.3, 151.7, 131.4, 129.2, 122.8, 105.2, 31.9, 30.7, 25.9, 12.0. **FT-IR** (thin layer): ν_{max} = 1727, 1690, 1364, 1304, 1251, 1143, 954, 768, 690. **HR-MS** (ESI): *m/z* = 310.1163, calcd. for $C_{15}H_{17}N_3O_3$+Na^+: 310.1162.

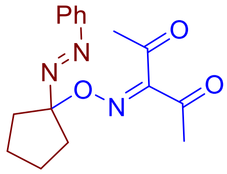

(E)-3-(((1-(phenyldiazenyl)cyclopentyl)oxy)imino)pentane-2,4-dione, **3cf** was synthesized as slightly yellow viscous gum (82%, purified by column chromatography with DCM as eluent). ^{1}**H NMR** (300.13 MHz, $CDCl_3$): δ = 7.78–7.63 (m, 2H), 7.55–7.40 (m, 3H), 2.44 (s, 3H), 2.35 (s, 3H), 2.30–2.12 (m, 4H), 1.96–1.81 (m, 4H). ^{13}**C NMR** (75.47 MHz, $CDCl_3$): δ = 198.8, 194.8, 156.8, 151.7, 131.2, 129.2, 122.6, 115.6, 36.4, 30.5, 25.9, 24.8. **FT-IR** (thin layer): ν_{max} = 2959, 1725, 1685, 1363, 1302, 1188, 959, 766, 690. **HR-MS** (ESI): *m/z* = 340.1059, calcd. for $C_{16}H_{19}N_3O_3$+K^+: 340.1058.

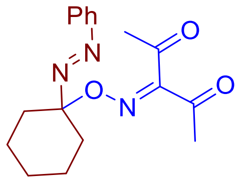

(E)-3-(((1-(phenyldiazenyl)cyclohexyl)oxy)imino)pentane-2,4-dione, **3cg** was synthesized as slightly yellow viscous gum (89%, purified by column chromatography with DCM as eluent). ^{1}**H NMR** (300.13 MHz, $CDCl_3$): δ = 7.74–7.64 (m, 2H), 7.55–7.39 (m, 3H), 2.46 (s, 3H), 2.45 (s, 3H), 2.19–2.06 (m, 2H), 1.92–1.68 (m, 5H), 1.67–1.46 (m, 2H), 1.45–1.27 (m, 1H). ^{13}**C NMR**

(75.47 MHz, $CDCl_3$): δ = 198.9, 194.8, 156.6, 151.7, 131.2, 129.2, 122.6, 105.2, 32.1, 30.6, 25.9, 25.0, 21.9. **FT-IR** (thin layer): ν_{max} = 2938, 2863, 1727, 1689, 1599, 1450, 1420, 1363, 1304, 1275, 1256, 1195, 1159, 1146, 1069, 1023, 983, 960, 928, 911, 766, 691. **HR-MS** (ESI): *m/z* = 316.1654, calcd. for $C_{17}H_{21}N_3O_3+H^+$: 316.1656.

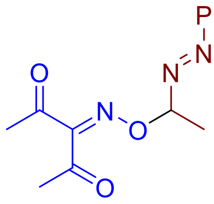

(*E*)-3-((1-(phenyldiazenyl)ethoxy)imino)pentane-2,4-dione, **3da** was synthesized as pale brown gum (74%, purified by column chromatography with DCM as eluent). ^{1}**H NMR** (300 MHz, $CDCl_3$): δ = 7.79–7.69 (m, 2H), 7.54–7.44 (m, 3H), 5.67 (q, *J* = 6.3 Hz, 1H), 2.45 (s, 3H), 2.34 (s, 3H), 1.59 (d, *J* = 6.3 Hz, 3H). ^{13}**C NMR** (75 MHz, $CDCl_3$) δ = 198.2, 194.5, 156.8, 151.5, 131.7, 129.3, 122.8, 103.5, 30.6, 25.9, 17.5. **FT-IR** (thin layer): ν_{max} = 1727, 1691, 1365, 1299, 1107, 1088, 1060, 965, 770, 691. **HR-MS** (ESI): *m/z* = 300.0733, calcd. for $C_{13}H_{15}N_3O_3+K^+$: 300.0745.

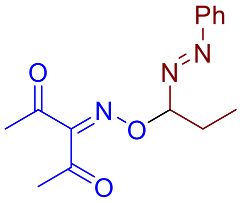

(*E*)-3-((1-(phenyldiazenyl)propoxy)imino)pentane-2,4-dione, **3db** was synthesized as pale yellow gum (59%, purified by column chromatography with DCM as eluent). ^{1}**H NMR** (300 MHz, $CDCl_3$): δ = 7.83–7.64 (m, 2H), 7.60–7.39 (m, 3H), 5.55–5.41 (m, 1H), 2.45 (s, 3H), 2.33 (s, 3H), 2.15–1.88 (m, 2H), 1.05 (t, *J* = 7.5 Hz, 3H). ^{13}**C NMR** (75 MHz, $CDCl_3$) δ = 198.2, 194.6, 156.9, 151.5, 131.6, 129.3, 122.8, 107.9, 30.6, 25.9, 25.3, 8.7. **FT-IR** (thin layer): ν_{max} = 1726, 1691, 1363, 1301, 1022, 988, 950, 769, 691. **HR-MS** (ESI): *m/z* = 298.1152, calcd. for $C_{14}H_{17}N_3O_3+Na^+$: 298.1162.

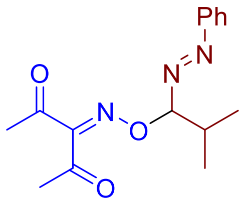

(*E*)-3-((2-methyl-1-(phenyldiazenyl)propoxy)imino)pentane-2,4-dione, **3dc** was synthesized as yellow gum (68%, purified by column chromatography with DCM as eluent). ^{1}**H NMR** (300 MHz, DMSO-d_6): δ = 7.77–7.68 (m, 2H), 7.62–7.49 (m, 3H), 5.42 (d, *J* = 5.5 Hz, 1H), 2.40 (s, 3H), 2.39–2.28 (m, 1H), 2.26 (s, 3H), 0.99 (t, *J* = 7.5 Hz, 6H). ^{13}**C NMR** (75 MHz, DMSO-

d_6): δ = 198.3, 193.7, 156.7, 150.9, 131.8, 129.4, 122.3, 109.1, 31.1, 30.0, 25.5, 17.5, 16.6. **FT-IR** (thin layer): ν_{max} = 2970, 1726, 1691, 1364, 1299, 1020, 998, 959, 769, 691. **HR-MS** (ESI): *m/z* = 290.1500, calcd. for $C_{15}H_{19}N_3O_3+H^+$: 290.1499.

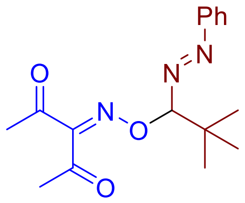

(*E*)-3-((2,2-dimethyl-1-(phenyldiazenyl)propoxy)imino)pentane-2,4-dione, **3dd** was synthesized as pale yellow gum (21%, purified by column chromatography with DCM as eluent). **^{1}H NMR** (300 MHz, $CDCl_3$): δ = 7.80–7.69 (m, 2H), 7.53–7.44 (m, 3H), 5.27 (s, 1H), 2.44 (s, 3H), 2.29 (s, 3H), 1.08 (s, 9H). **^{13}C NMR** (75 MHz, $CDCl_3$): δ = 198.1, 194.5, 156.8, 151.6, 131.6, 129.3, 122.9, 112.1, 36.0, 30.4, 25.7. **FT-IR** (thin layer): ν_{max} = 2973, 1727, 1693, 1365, 1300, 1021, 999, 959. **HR-MS** (ESI): *m/z* = 326.1474, calcd. for $C_{16}H_{21}N_3O_3+Na^+$: 326.1475.

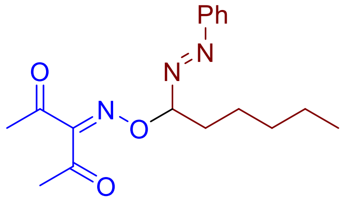

(*E*)-3-(((1-(phenyldiazenyl)hexyl)oxy)imino)pentane-2,4-dione, **3de** was synthesized as yellow gum (70%, purified by column chromatography with DCM as eluent). **^{1}H NMR** (300 MHz, $CDCl_3$): δ = 7.80–7.67 (m, 2H), 7.55–7.42 (m, 3H), 5.55 (dd, *J* = 7.7, 5.1 Hz, 1H), 2.45 (s, 3H), 2.33 (s, 3H), 2.11–1.76 (m, 2H), 1.55–1.40 (m, 2H), 1.40–1.23 (m, 4H), 0.89 (t, *J* = 6.9 Hz, 3H). **^{13}C NMR** (75 MHz, $CDCl_3$): δ = 198.2, 194.6, 156.8, 151.5, 131.6, 129.2, 122.8, 107.0, 31.8, 31.6, 30.6, 25.9, 23.9, 22.5, 14.1. **FT-IR** (thin layer): ν_{max} = 2956, 2931, 1727, 1691, 1363, 1299, 964. **HR-MS** (ESI): *m/z* = 318.1810, calcd. for $C_{17}H_{23}N_3O_3+H^+$: 318.1812.

X-ray single-crystal diffraction: Structure determination of compound 3ag

X-ray diffraction data were collected at 100K on a Bruker Quest D8 diffractometer equipped with a Photon-III area-detector (graphite monochromator, shutterless φ- and ω-scan technique), using Mo K_α-radiation (0.71073 Å). The intensity data were integrated by the SAINT program[13] and corrected for absorption and decay using SADABS.[14] The structure was solved by direct methods using SHELXT[15] and refined on F^2 using SHELXL-2018.[16] All non-hydrogen atoms were refined with anisotropic displacement parameters. Hydrogen atoms were placed in ideal calculated positions and refined as riding atoms with relative isotropic displacement parameters. The SHELXTL program suite[13] was used for molecular graphics.

Table S1. Crystal data and structure refinement for **3ag**.

Empirical formula	$C_{19}H_{18}BrN_3O_3$	
Formula weight	416.27	
Temperature	100(2) K	
Wavelength	0.71073 Å	
Crystal system	Triclinic	
Space group	P-1	
Unit cell dimensions	a = 8.5693(3) Å	α = 77.8358(8)°
	b = 8.6800(3) Å	β = 81.1246(9)°
	c = 13.7733(4) Å	γ = 69.2122(8)°
Volume	932.73(5) $Å^3$	
Z	2	
Density (calculated)	1.480 g/cm^3	
Absorption coefficient	2.227 mm^{-1}	
F(000)	424	
Crystal size	0.58 x 0.55 x 0.43 mm^3	
Theta range for data collection	2.546 to 37.807°.°	
Index ranges	-14<=h<=14, -14<=k<=14, -23<=l<=23	
Reflections collected	57161	
Independent reflections	9989 [R(int) = 0.0388]	
Observed reflections	8239	
Completeness to theta = 25.242°	99.9 %	
Absorption correction	Semi-empirical from equivalents	
Max. and min. transmission	0.2763 and 0.1390	
Refinement method	Full-matrix least-squares on F^2	
Data / restraints / parameters	9989 / 0 / 239	
Goodness-of-fit on F^2	1.026	
Final R indices [I>2sigma(I)]	R1 = 0.0322, wR2 = 0.0812	
R indices (all data)	R1 = 0.0439, wR2 = 0.0861	
Largest diff. peak and hole	1.168 and -1.302 e.$Å^{-3}$	

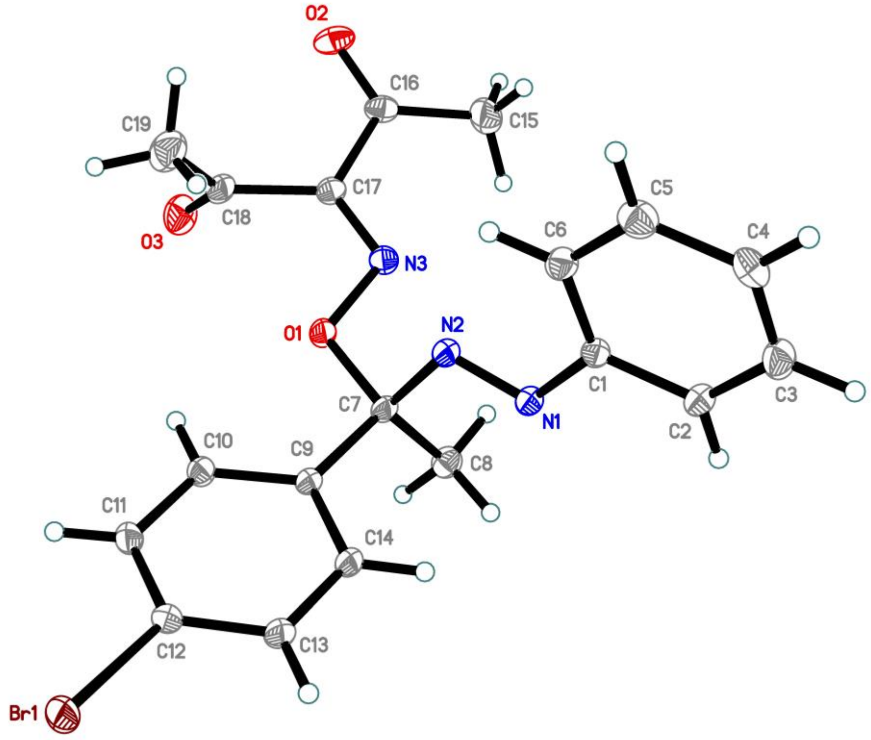

Figure S1. Crystal structure of compound **3ag**, showing the atomic numbering and 50% probability displacement ellipsoids

Compound **3ag** crystallizes in monoclinic space group P-1 (Fig. S1).

Computational details

DFT calculations were conducted by B97-3c composite method[17] including D3 dispersion correction[18,19] as implemented in Orca 5.0.4 program[20]. Main conformers of diacetyliminoxyl **1** and hydrazone **2ca** were considered in all calculations. Presented results correspond to 218.15 K and 1 atm. Cartesian coordinates and energy values of optimized structures of **1a**, **1**, **2ca**, **A**, **B**, and transition states for path I and path II are given below. Optimized geometries were visualized by Avogadro 1.2 program.[21]

Diacetyl oxime 1(a)

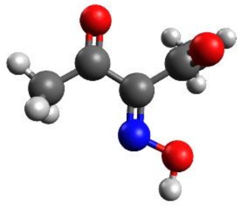

Unpaired electrons: 0

Imaginary frequencies: no

Final Gibbs free energy ... -474.83198681 Eh

Total Enthalpy ... -474.78529015 Eh

Electronic energy ... -474.91396710 Eh

N	0.06970225033778	1.54562566196768	0.00854359883869
C	0.08841033853849	0.26382198676505	0.07371521376140
C	-1.15107648533480	-0.59782234957868	0.22487853008821
C	1.40196017603439	-0.42333735426638	0.03322902618181
O	-1.54265829649431	-0.86755404828874	1.34410855371014
O	1.40954528713797	-1.64891879358009	0.06475775394007
O	-1.20870418302714	2.06009556558835	0.04595806704607
H	-1.07082430823139	3.01644889783864	-0.01859299086395
C	-1.79598390678403	-1.05316711770259	-1.03488608550523
H	-2.69013432158179	-1.63226785517786	-0.82657438779557
H	-2.03918075734542	-0.19077263723676	-1.65639698571686
H	-1.08467615232901	-1.65537516851252	-1.60210325386446
C	2.64933690106215	0.39673910326443	-0.03599672505283
H	2.61027263562725	1.09594619155812	-0.86862938540732
H	2.75198013657635	0.99236558635661	0.87075641067229
H	3.50731168581351	-0.25923066899526	-0.13816534003246

Diacetyliminoxyl radical (1)

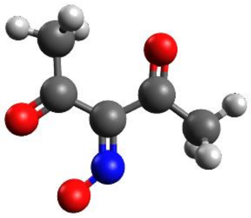

Unpaired electrons: 1

Imaginary frequencies: no

Final Gibbs free energy ... -474.22068873 Eh

Total Enthalpy ... -474.17480563 Eh

Electronic energy ... -474.29128868 Eh

C	-1.33538414773864	-1.96184929957175	-0.00010017151065
C	-1.27946371058432	-0.46700782514113	-0.00040563769729
H	-2.37450916379125	-2.27239775783076	0.00049472164576

H	-0.81859334007738	-2.35823706627575	-0.87070509570917
H	-0.81768566092729	-2.35795354049976	0.87010043278926
C	0.04990145680954	0.22072226254471	-0.00041456707655
C	1.38326434561685	-0.42221970562381	-0.00060624263912
C	2.58086993574630	0.48363325680431	0.00010539993198
H	2.57346739547275	1.13127684194830	-0.87556596752056
H	3.48231145114325	-0.11952463580711	0.00046637524965
H	2.57262527054406	1.13105670448193	0.87593869695890
N	0.03632679688412	1.52809581527337	-0.00024512776745
O	-0.89676757675779	2.30058401146669	-0.00011685457034
O	-2.29609797732566	0.20998647163861	0.00059004640418
O	1.49821192498545	-1.64031853340783	-0.00010100848861

1-phenyl-2-(propan-2-ylidene)hydrazine (hydrazone 2ca)

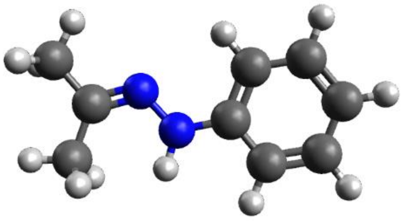

Unpaired electrons: 0

Imaginary frequencies: no

Final Gibbs free energy ... -459.29164258 Eh

Total Enthalpy ... -459.24325792 Eh

Electronic energy ... -459.44600868 Eh

C	-2.68828473691068	-0.10798480311287	0.02771241309834
C	-3.15896750595697	1.31340080568908	0.00973857270202
H	-2.80479835013175	1.83964560149747	-0.87979305768191
H	-4.24298035489196	1.36556371948182	0.01771621721063
H	-2.79269322723717	1.86764647378725	0.87737274418745
C	0.84341944945057	0.20126289738187	0.00059373435521
C	1.76992448646290	1.25330467736635	0.01876207241468
C	1.31610657137754	-1.11631705582259	0.00025776409773
C	3.12821220921590	0.98988995939636	0.03525792802819
H	1.41403427492504	2.27641467402569	0.02005453646898

C	2.68041616682092	-1.36154042942581	0.01746875855271
H	0.61200986279974	-1.93365766507553	-0.01265148788478
C	3.59952948891468	-0.31905815019590	0.03475568779620
H	3.82503601980865	1.81786918352351	0.04967967676972
H	3.02738675709240	-2.38696238873642	0.01716708495444
H	4.66172093115328	-0.52102194782030	0.04805596986220
N	-1.44831891686306	-0.45898915543771	0.01231611269370
N	-0.50225727949582	0.50549952080282	-0.02496421741167
H	-0.76299821766264	1.48070371931762	0.01709855475445
C	-3.70944952532095	-1.19255080486344	0.06475426018305
H	-4.34260629361971	-1.09932941111583	0.94978551909342
H	-4.37462058201747	-1.12940871641359	-0.79929750923365
H	-3.23994122791342	-2.17221270424985	0.07306866498886

Hydrazyl radical (A)

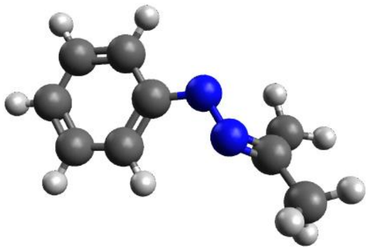

Unpaired electrons: 1

Imaginary frequencies: no

Final Gibbs free energy ... -458.67006756 Eh

Total Enthalpy ... -458.62140860 Eh

Electronic energy ... -458.81114779 Eh

C	-1.72338616027636	2.71428469319100	0.22077940605414
C	-0.72622767139289	2.50766868189767	1.31216198697860
H	0.18104509298792	3.07704571522331	1.12605366397245
H	-1.13648232724174	2.84718908902932	2.26504759995647
H	-0.48897000170441	1.45108853103101	1.41968230462667
C	-3.75822670237235	0.18554628823212	1.02464620133219
C	-3.79792950046681	-1.16724211828324	1.42769582834566
C	-4.87414530822482	1.00703747685982	1.30363120159041

C	-4.89900688259140	-1.67020028425693	2.08858582276639
H	-2.94322464269852	-1.79360779123958	1.21106621686552
C	-5.96986247227073	0.48672231167988	1.96151612192368
H	-4.85948632092238	2.04162015133829	0.99353684068354
C	-5.99314213313639	-0.84951975888042	2.35983027948697
H	-4.91333287945769	-2.70781209614253	2.39425892196645
H	-6.82025745869417	1.12327433347172	2.16831933408262
H	-6.85684141493623	-1.24730204289183	2.87473683366425
N	-2.62294760358929	1.84380547466124	-0.07175586597223
N	-2.61585690500284	0.60863336080034	0.40834074110731
C	-1.70251713840699	3.99535279174785	-0.53550333929676
H	-1.78744327673710	4.84304979731567	0.14613016940992
H	-0.74953087045499	4.11028041889706	-1.05539058684360
H	-2.50974399140980	4.04016445531821	-1.26042722870066

Diacetyliminoxyl radical 1 adduct to hydrazone 2ca (B)

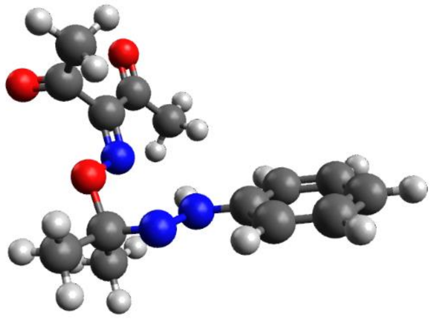

Unpaired electrons: 1

Imaginary frequencies: no

Final Gibbs free energy ... -933.48547166 Eh

Total Enthalpy ... -933.41400148 Eh

Electronic energy ... -933.73578724 Eh

C	0.20208540057188	2.66697044318437	-1.44286809710738
H	-0.55294441937583	3.44936971465738	-1.44903454981695
H	1.13073696934283	3.07566803474246	-1.83307730219112
H	-0.12727525428895	1.87073852170414	-2.10292020364739
C	-2.45537056683475	0.00740354934420	0.38428907799657
C	-2.92719595691344	-1.08197545518958	-0.35908881243144

C	-3.14486128634428	0.41848900361807	1.53092881806197
C	-4.07128530401349	-1.74646687842773	0.04126119929755
H	-2.39309940117444	-1.39777631928556	-1.24581304649687
C	-4.28810438693986	-0.25959205196204	1.91642942925229
H	-2.77707133953158	1.25682765826169	2.10156177027238
C	-4.76099670377059	-1.34175827100304	1.18068594930122
H	-4.42721214666704	-2.58696353356826	-0.53898653117118
H	-4.81722357579462	0.06212412304624	2.80355867195593
H	-5.65533379714678	-1.86379029194511	1.49135821454483
N	-0.76376536018175	1.65658128708482	0.61878690122883
N	-1.31633689450645	0.65677545700822	-0.05201259323035
H	-0.83362752640751	0.25687190408940	-0.85244504024837
C	0.93388726634611	3.30464593773993	0.87419986002085
H	1.85760273274683	3.70950263374563	0.46981695888761
H	0.19756121201662	4.10191674506906	0.92944978109626
H	1.11444343105022	2.93045485208165	1.87884128955564
C	0.40533129577487	2.20034105891560	-0.01224958859179
O	1.52979315458969	1.19513271083804	0.03121839293010
N	1.32899146152188	0.15593448636255	-0.82560201624782
C	2.21033664143136	-0.77547762341932	-0.70491952953750
C	3.37523484582571	-0.69832815114747	0.26062970332883
C	2.08813309077490	-1.97170540927001	-1.55975140115902
C	3.13011641122726	-1.23771914571563	1.62487801780537
O	4.42594263980457	-0.21449578250983	-0.11632447000970
C	0.94892045439329	-2.05372011365357	-2.52685141339844
O	2.92280986455075	-2.86453552613147	-1.43722592606142
H	4.00023323554734	-1.10216977055046	2.25958505161548
H	2.26049408539129	-0.74627864659645	2.06245002308525
H	2.88449978798086	-2.29812335426272	1.54876371048518
H	-0.00164151365853	-2.00535008457258	-1.99772413117195
H	1.01205019185409	-2.98474881101989	-3.08011804610397
H	0.96922878080754	-1.21118930126280	-3.21599308209943

Transition state for hydrogen atom abstraction from hydrazone 2ca by diacetyliminoxyl radical 1 (Path I)

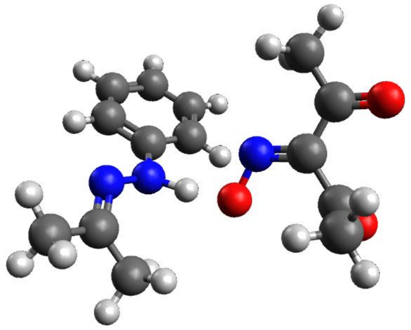

Unpaired electrons: 1

Imaginary frequencies: 1

Final Gibbs free energy ... -933.49075506 Eh

Total Enthalpy ... -933.41654700 Eh

Electronic energy ... -933.73278728 Eh

C	-2.67860679090951	-1.89648384316536	0.54948187300355
C	-2.21349691934370	-2.69406541384970	-0.61630877431048
H	-1.23401112093057	-3.12099090918240	-0.39076607843662
H	-2.88926453235783	-3.52519973779008	-0.79976550850007
H	-2.11596148367138	-2.09540955521574	-1.51662080451967
C	-1.84233979986778	1.35247254848249	-0.36616905182471
C	-0.89416093642782	1.99661365797425	-1.19094765247673
C	-2.89161455605118	2.09852871890858	0.20894655011364
C	-0.99581000643264	3.35085267846665	-1.42110394078054
H	-0.09703893464611	1.41927658179076	-1.63372013123499
C	-2.97551135014645	3.44992975529813	-0.03783327808182
H	-3.61056883023651	1.60290197412827	0.84180799133073
C	-2.03279527196658	4.08532682673897	-0.84930885363436
H	-0.26700398066986	3.84213868113130	-2.05000461453565
H	-3.77753397875648	4.02412622600595	0.40463997080790
H	-2.10784219008135	5.14799821418774	-1.03246808129819
N	-2.51251558185589	-0.61514819621273	0.69330444415441
N	-1.71422625673441	0.00685602376814	-0.19308017247196
C	-3.39967819943933	-2.60226185348514	1.63482633175767
H	-4.32654122283430	-3.03016502277996	1.24882676727505

H	-2.80094284232157	-3.44251639167538	1.98972906711072
H	-3.62541066195303	-1.94055397903985	2.46422746017884
O	0.58855737635927	-0.98181742929971	-0.52099404878882
N	1.36916130546165	-0.12792640383936	0.10693363272955
C	2.64715001430066	-0.38134769325150	0.08139044962258
C	3.20450362011337	-1.58447810306832	-0.64066550444963
C	3.56497584941896	0.51627341661213	0.76573881338546
C	3.25688354888892	-2.85107057610939	0.14497393136953
O	3.57379235165927	-1.49027918813163	-1.79889123430014
C	3.01965548097088	1.74449013515227	1.43872539190041
O	4.77311854177684	0.25397068444044	0.77841290055731
H	3.61323038951389	-3.67809719678589	-0.46202061043530
H	3.91563355439710	-2.70764394248505	1.00296149827240
H	2.26648439203522	-3.07242523129194	0.54353793737449
H	2.55714061488931	2.40509338235350	0.70664188127719
H	3.82883878371615	2.26756818221565	1.93872860745157
H	2.24721958844201	1.48357761405276	2.15911789502286
H	-0.70115596430925	-0.46753663504886	-0.42493705461618

Transition state for diacetyliminoxyl radical 1 addition to hydrazone 2ca (Path II)

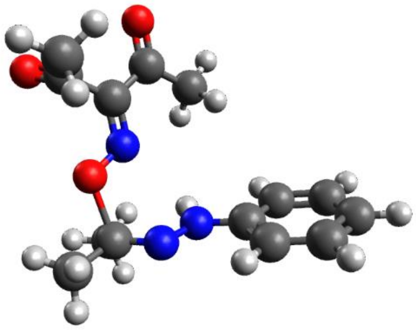

Unpaired electrons: 1

Imaginary frequencies: 1

Final Gibbs free energy ... -933.48201377 Eh
Total Enthalpy ... -933.41053113 Eh
Electronic energy ... -933.73092950 Eh

C	0.27503802918850	2.72875138211409	-1.44429719853980

H	-0.57098327533227	3.37744680671123	-1.67204226740085
H	1.19138511223518	3.26243018180790	-1.67430515241035
H	0.22494528516479	1.86199991786022	-2.09922380573659
C	-2.54166763596767	0.12409290859729	0.14934655162066
C	-3.12931993714930	-0.73935017695603	-0.78484566037081
C	-3.05023429246225	0.19890325120213	1.45129888264606
C	-4.21048184080145	-1.51659297855103	-0.41644411877056
H	-2.73069905575516	-0.79247828402190	-1.78963826149161
C	-4.13408693506004	-0.58581782014549	1.80032681981692
H	-2.59115625534702	0.86303510570720	2.16640291152016
C	-4.72103089405013	-1.44584835997663	0.87645096392150
H	-4.65806166975304	-2.18193994437398	-1.14184954057883
H	-4.52559299716710	-0.52741319242802	2.80689424994056
H	-5.56732957675994	-2.05526102704758	1.16116868753842
N	-0.82406713412918	1.70951837542265	0.55579014107598
N	-1.46482702690720	0.88421082737468	-0.25713626351273
H	-1.11904272292404	0.72038374995890	-1.19574966621820
C	0.78132822972775	3.41551701216008	0.94455218615434
H	1.80070114046036	3.67702608800313	0.67433045712908
H	0.17182637684762	4.31589598019298	0.86732565355112
H	0.75461075718273	3.06825439477512	1.97324515170304
C	0.24090071758986	2.37393643751057	0.01637416488474

O	1.63957837305631	1.20190322979606	0.10492115377978
N	1.31501003832916	0.11026236533061	-0.54777222875358
C	2.19218147934141	-0.84525150624459	-0.55280665570219
C	3.54097590086083	-0.71526891607733	0.11831695631920
C	1.85935501177464	-2.09713378089655	-1.23225224973420
C	3.59664646490207	-1.10785944849598	1.55393815715428
O	4.49420838576509	-0.29789094283756	-0.51517947115887
C	0.51248646164902	-2.23678876185190	-1.87882440969830
O	2.68479419848239	-3.01233865993344	-1.23355081930947
H	4.58707516542079	-0.94498289679690	1.96776319468071
H	2.85418295042570	-0.53864429842055	2.11469010569789
H	3.31979105937952	-2.15901845532337	1.64789049440119
H	-0.26642593815953	-2.23632565090172	-1.11701009461966
H	0.47370575181046	-3.17054428653096	-2.43037770976349
H	0.30338499413126	-1.40136221871340	-2.54291145376550

References

(1) Andleeb, H.; Tehseen, Y.; Ali Shah, S. J.; Khan, I.; Iqbal, J.; Hameed, S. Identification of Novel Pyrazole–Rhodanine Hybrid Scaffolds as Potent Inhibitors of Aldose Reductase: Design, Synthesis, Biological Evaluation and Molecular Docking Analysis. *RSC Adv.* **2016**, *6* (81), 77688–77700. https://doi.org/10.1039/C6RA14531K.

(2) Chen, Z.; Li, H.; Dong, W.; Miao, M.; Ren, H. I_2 -Catalyzed Oxidative Coupling Reactions of Hydrazones and Amines and the Application in the Synthesis of 1,3,5-Trisubstituted 1,2,4-Triazoles. *Org. Lett.* **2016**, *18* (6), 1334–1337. https://doi.org/10.1021/acs.orglett.6b00277.

(3) Harej, M.; Dolenc, D. Autoxidation of Hydrazones. Some New Insights. *J. Org. Chem.* **2007**, *72* (19), 7214–7221. https://doi.org/10.1021/jo071091m.

(4) Qian, H.; Nguyen, H. D.; Lv, L.; Chen, S.; Li, Z. Chemo-, Stereo- and Regioselective Fluoroallylation/Annulation of Hydrazones with *Gem* -Difluorocyclopropanes via Tunable Palladium/NHC Catalysis. *Angew Chem Int Ed* **2023**, *62* (23), e202303271. https://doi.org/10.1002/anie.202303271.

(5) Su, Y.-M.; Hou, Y.; Yin, F.; Xu, Y.-M.; Li, Y.; Zheng, X.; Wang, X.-S. Visible Light-Mediated C–H Difluoromethylation of Electron-Rich Heteroarenes. *Org. Lett.* **2014**, *16* (11), 2958–2961. https://doi.org/10.1021/ol501094z.

(6) Yang, X.-L.; Peng, X.-X.; Chen, F.; Han, B. TEMPO-Mediated Aza-Diels–Alder Reaction: Synthesis of Tetrahydropyridazines Using Ketohydrazones and Olefins. *Org. Lett.* **2016**, *18* (9), 2070–2073. https://doi.org/10.1021/acs.orglett.6b00702.

(7) Zhang, G.; Miao, J.; Zhao, Y.; Ge, H. Copper-Catalyzed Aerobic Dehydrogenative Cyclization of N-Methyl-N-Phenylhydrazones: Synthesis of Cinnolines. *Angew. Chem. Int. Ed.* **2012**, *51* (33), 8318–8321. https://doi.org/10.1002/anie.201204339.

(8) Kašpar, M.; Hamplová, V.; Novotná, V.; Pacherová, O. The Effect of the Alkyl Chain Length on the Mesomorphic Properties of New Lactic Acid Derivatives. *Liquid Crystals* **2014**, *41* (8), 1179–1187. https://doi.org/10.1080/02678292.2014.910315.

(9) Katagiri, T.; Ota, S.; Ohira, T.; Yamao, T.; Hotta, S. Synthesis of Thiophene/Phenylene Co-Oligomers. V. Functionalization at Molecular Terminals toward Optoelectronic Device Applications. *Journal of Heterocyclic Chemistry* **2007**, *44* (4), 853–862. https://doi.org/10.1002/jhet.5570440417.

(10) Trofimov, B. A.; Schmidt, E. Yu.; Zorina, N. V.; Ivanova, E. V.; Ushakov, I. A. Transition-Metal-Free Superbase-Promoted Stereoselective α-Vinylation of Ketones with Arylacetylenes: A General Strategy for Synthesis of β,γ-Unsaturated Ketones. *J. Org. Chem.* **2012**, *77* (16), 6880–6886. https://doi.org/10.1021/jo301005p.

(11) Pünner, F.; Sohtome, Y.; Sodeoka, M. Solvent-Dependent Copper-Catalyzed Synthesis of Pyrazoles under Aerobic Conditions. *Chem. Commun.* **2016**, *52* (98), 14093–14096. https://doi.org/10.1039/C6CC06935E.

(12) Krylov, I. B.; Paveliev, S. A.; Shelimov, B. N.; Lokshin, B. V.; Garbuzova, I. A.; Tafeenko, V. A.; Chernyshev, V. V.; Budnikov, A. S.; Nikishin, G. I.; Terent'ev, A. O. Selective Cross-Dehydrogenative C–O Coupling of N-Hydroxy Compounds with Pyrazolones. Introduction of the Diacetyliminoxyl Radical into the Practice of Organic Synthesis. *Org. Chem. Front.* **2017**, *4* (10), 1947–1957. https://doi.org/10.1039/C7QO00447H.

(13) Bruker. APEX-III. Bruker AXS Inc., Madison, Wisconsin, USA, 2019.

(14) Krause, L.; Herbst-Irmer, R.; Sheldrick, G. M.; Stalke, D. Comparison of Silver and Molybdenum Microfocus X-Ray Sources for Single-Crystal Structure Determination. *J Appl Crystallogr* **2015**, *48* (1), 3–10. https://doi.org/10.1107/S1600576714022985.

(15) Sheldrick, G. M. *SHELXT* – Integrated Space-Group and Crystal-Structure Determination. *Acta Crystallogr A Found Adv* **2015**, *71* (1), 3–8. https://doi.org/10.1107/S2053273314026370.

(16) Sheldrick, G. M. Crystal Structure Refinement with *SHELXL*. *Acta Crystallogr C Struct Chem* **2015**, *71* (1), 3–8. https://doi.org/10.1107/S2053229614024218.

(17) Brandenburg, J. G.; Bannwarth, C.; Hansen, A.; Grimme, S. B97-3c: A Revised Low-Cost Variant of the B97-D Density Functional Method. *The Journal of Chemical Physics* **2018**, *148* (6), 064104. https://doi.org/10.1063/1.5012601.

(18) Grimme, S.; Ehrlich, S.; Goerigk, L. Effect of the Damping Function in Dispersion Corrected Density Functional Theory. *J Comput Chem* **2011**, *32* (7), 1456–1465. https://doi.org/10.1002/jcc.21759.

(19) Grimme, S.; Antony, J.; Ehrlich, S.; Krieg, H. A Consistent and Accurate *Ab Initio* Parametrization of Density Functional Dispersion Correction (DFT-D) for the 94 Elements H-Pu. *The Journal of Chemical Physics* **2010**, *132* (15), 154104. https://doi.org/10.1063/1.3382344.

(20) Neese, F. Software Update: The ORCA Program System—Version 5.0. *WIREs Comput Mol Sci* **2022**, *12* (5), e1606. https://doi.org/10.1002/wcms.1606.

(21) Hanwell, M. D.; Curtis, D. E.; Lonie, D. C.; Vandermeersch, T.; Zurek, E.; Hutchison, G. R. Avogadro: An Advanced Semantic Chemical Editor, Visualization, and Analysis Platform. *J Cheminform* **2012**, *4* (1), 17. https://doi.org/10.1186/1758-2946-4-17.

The ^{1}H and ^{13}C spectra of synthesized compounds

^{1}H NMR (300.13 MHz, $CDCl_3$)

(*E*)-3-((1-phenyl-1-(phenyldiazenyl)ethoxy)imino)pentane-2,4-dione, 3aa

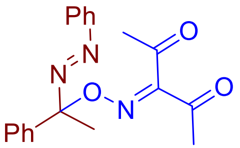

Title	i6169.{1H}.1.fid
Solvent	CDCl3
Temperature	300.0
Pulse Sequence	zg
Number of Scans	1
Receiver Gain	203.0
Relaxation Delay	0.0050
Pulse Width	9.0000
Acquisition Time	1.3518
Spectrometer Frequency	300.13
Spectral Width	6009.6
Lowest Frequency	-609.7
Nucleus	1H
Acquired Size	8124
Spectral Size	65536

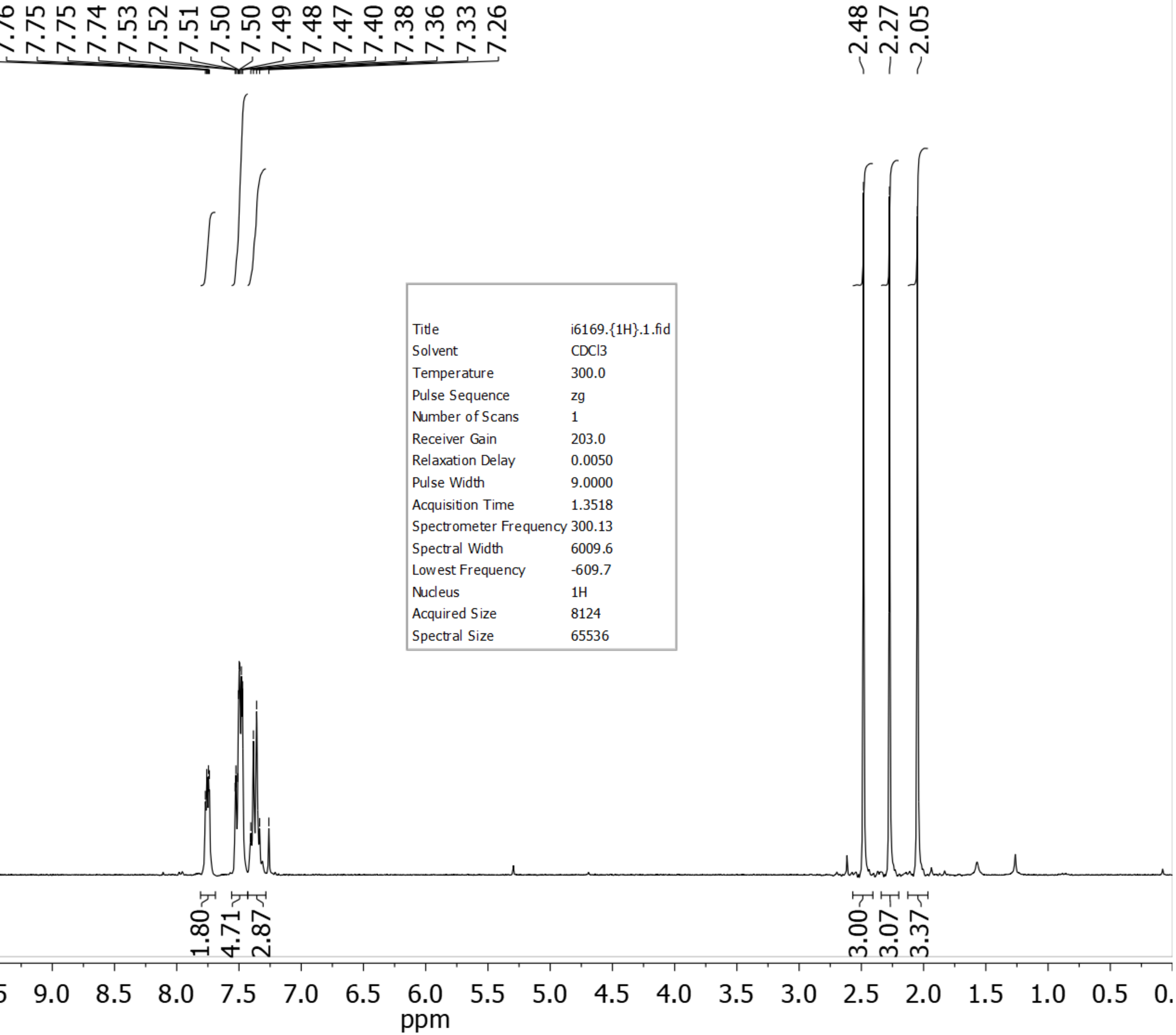

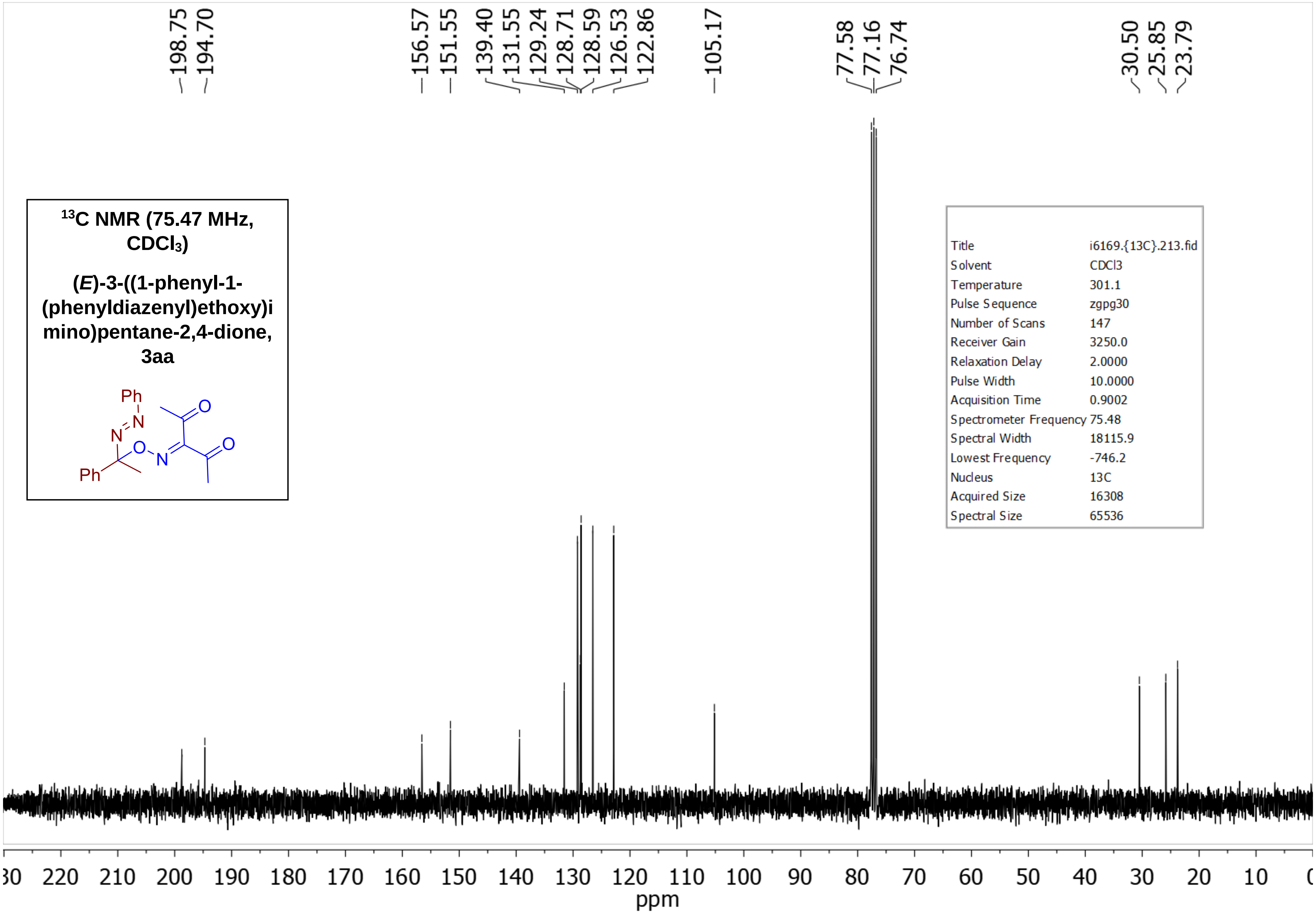
198.75
194.70
156.57
151.55
139.40
131.55
129.24
128.71
128.59
126.53
122.86
105.17
77.58
77.16
76.74
30.50
25.85
23.79
^{13}C NMR (75.47 MHz, $CDCl_3$)
(*E*)-3-((1-phenyl-1-(phenyldiazenyl)ethoxy)imino)pentane-2,4-dione, 3aa
Ph
N
N
O
N
O
O
Ph
Title i6169.{13C}.213.fid
Solvent CDCl3
Temperature 301.1
Pulse Sequence zgpg30
Number of Scans 147
Receiver Gain 3250.0
Relaxation Delay 2.0000
Pulse Width 10.0000
Acquisition Time 0.9002
Spectrometer Frequency 75.48
Spectral Width 18115.9
Lowest Frequency -746.2
Nucleus 13C
Acquired Size 16308
Spectral Size 65536
220
210
200
190
180
170
160
150
140
130
120
110
100
90
80
70
60
50
40
30
20
10
ppm

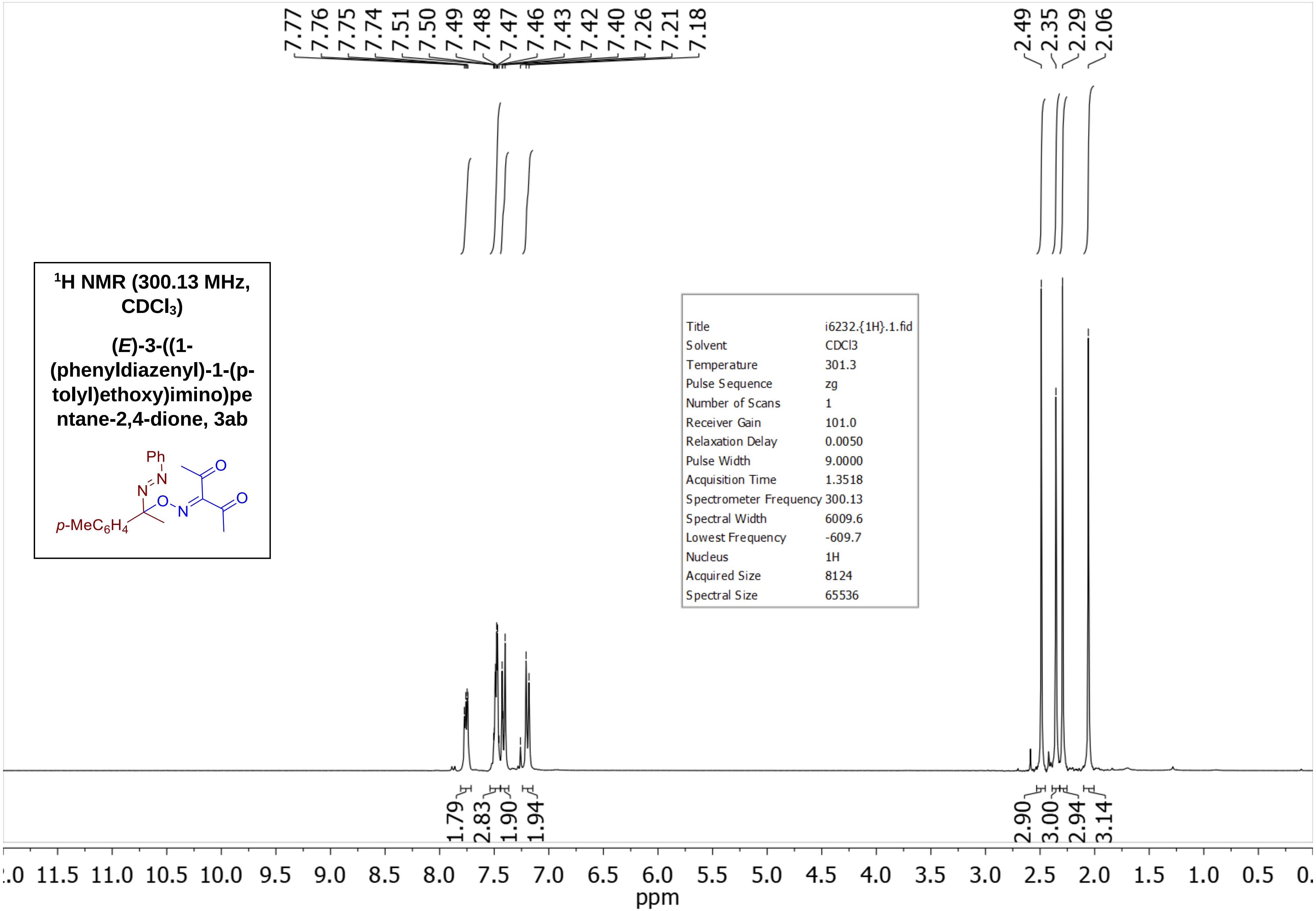

7.77
7.76
7.75
7.74
7.51
7.50
7.49
7.48
7.47
7.46
7.43
7.42
7.40
7.26
7.21
7.18
2.49
2.35
2.29
2.06
1H NMR (300.13 MHz, CDCl3)
(E)-3-((1-(phenyldiazenyl)-1-(p-tolyl)ethoxy)imino)pentane-2,4-dione, 3ab
Ph
N
N
O
N
O
O
p-MeC6H4
Title i6232.{1H}.1.fid
Solvent CDCl3
Temperature 301.3
Pulse Sequence zg
Number of Scans 1
Receiver Gain 101.0
Relaxation Delay 0.0050
Pulse Width 9.0000
Acquisition Time 1.3518
Spectrometer Frequency 300.13
Spectral Width 6009.6
Lowest Frequency -609.7
Nucleus 1H
Acquired Size 8124
Spectral Size 65536
1.79
2.83
1.90
1.94
2.90
3.00
2.94
3.14
11.5 11.0 10.5 10.0 9.5 9.0 8.5 8.0 7.5 7.0 6.5 6.0 5.5 5.0 4.5 4.0 3.5 3.0 2.5 2.0 1.5 1.0 0.5
ppm

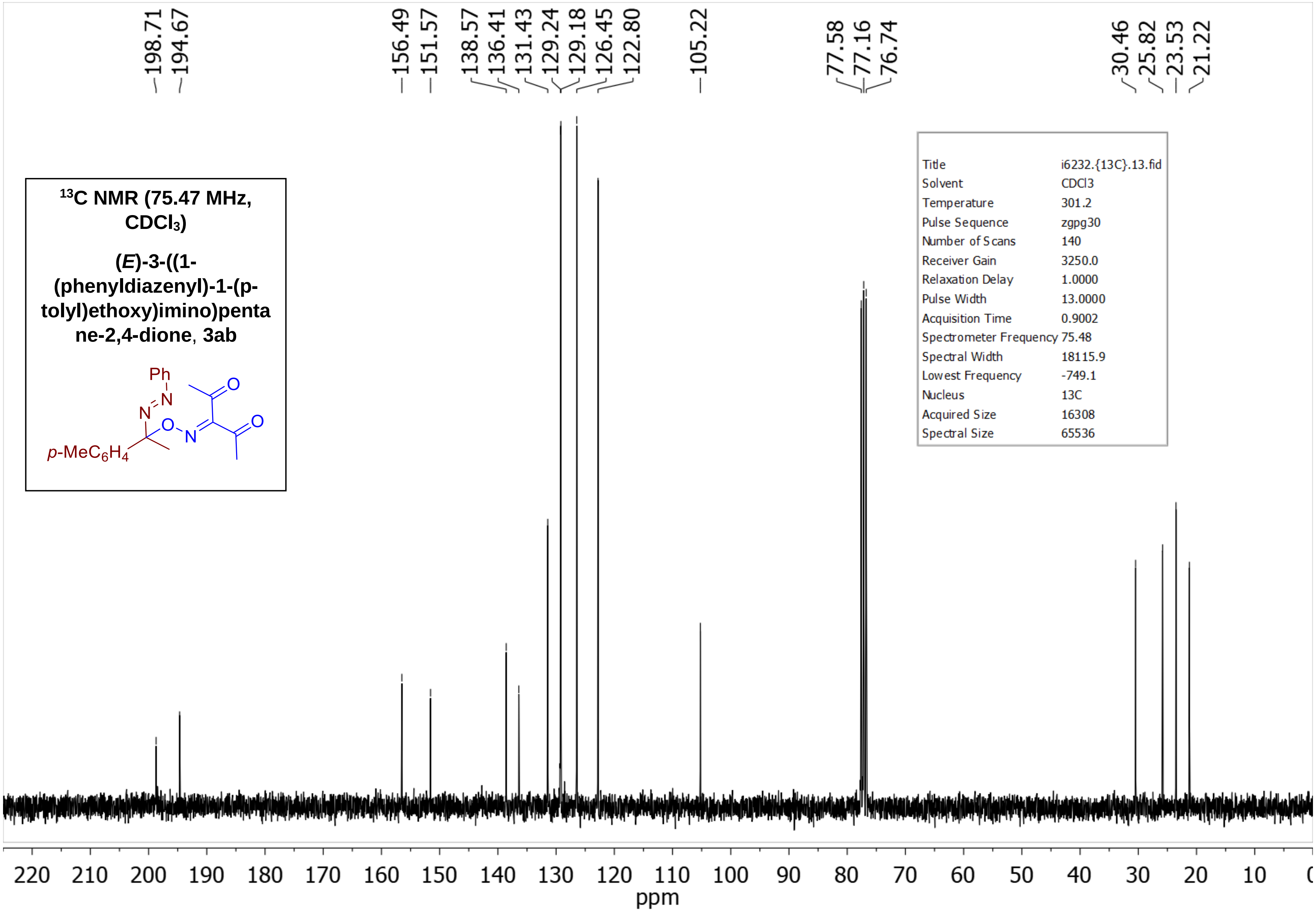

198.71
194.67
156.49
151.57
138.57
136.41
131.43
129.24
129.18
126.45
122.80
105.22
77.58
77.16
76.74
30.46
25.82
23.53
21.22
13C NMR (75.47 MHz, CDCl3)
(E)-3-((1-(phenyldiazenyl)-1-(p-tolyl)ethoxy)imino)pentane-2,4-dione, 3ab
Ph
N
N
O
N
O
O
p-MeC6H4
Title i6232.{13C}.13.fid
Solvent CDCl3
Temperature 301.2
Pulse Sequence zgpg30
Number of Scans 140
Receiver Gain 3250.0
Relaxation Delay 1.0000
Pulse Width 13.0000
Acquisition Time 0.9002
Spectrometer Frequency 75.48
Spectral Width 18115.9
Lowest Frequency -749.1
Nucleus 13C
Acquired Size 16308
Spectral Size 65536
220 210 200 190 180 170 160 150 140 130 120 110 100 90 80 70 60 50 40 30 20 10
ppm

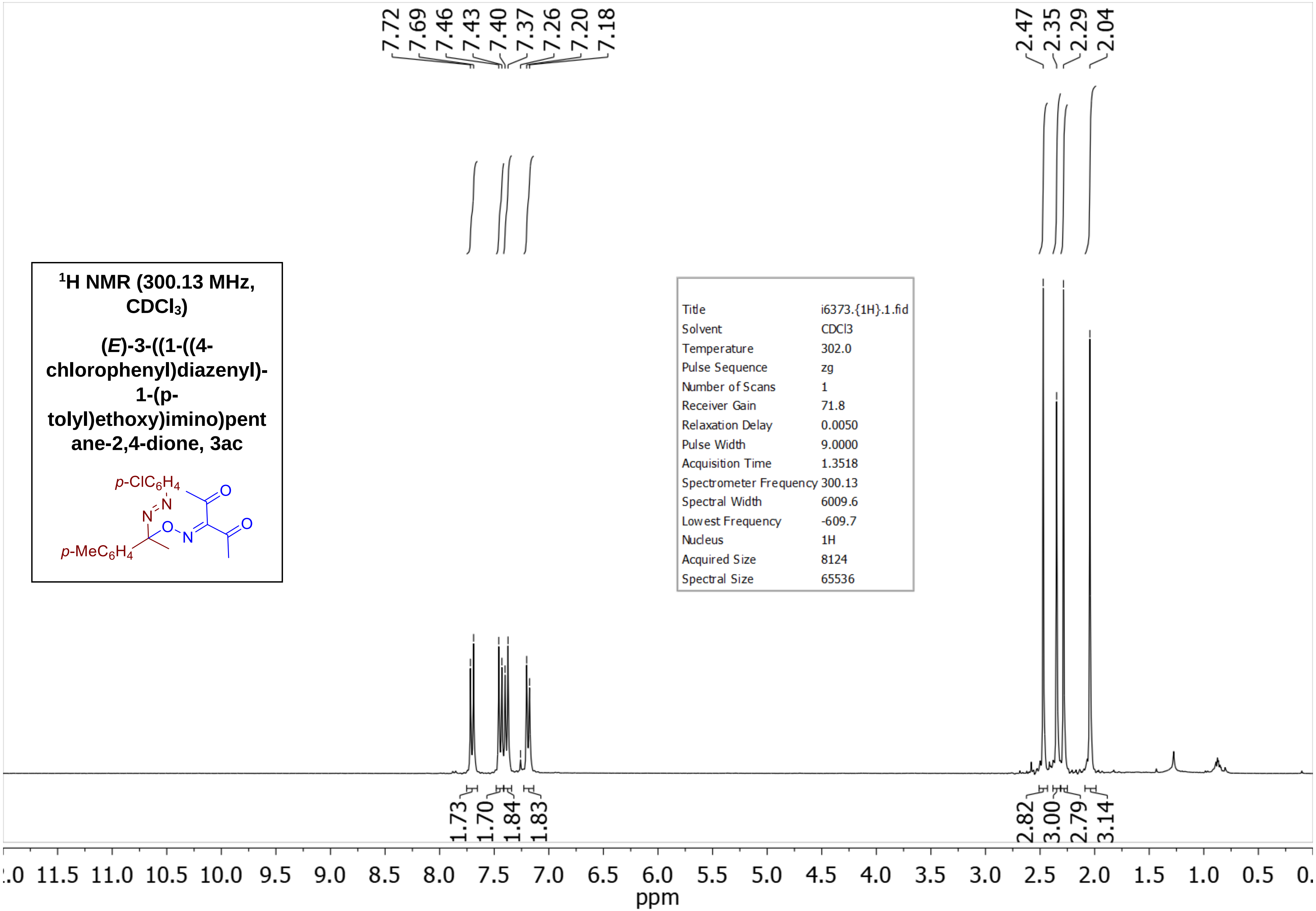
7.72
7.69
7.46
7.43
7.40
7.37
7.26
7.20
7.18
2.47
2.35
2.29
2.04
1H NMR (300.13 MHz, CDCl3)
(E)-3-((1-((4-chlorophenyl)diazenyl)-1-(p-tolyl)ethoxy)imino)pentane-2,4-dione, 3ac
p-ClC6H4
N
N
O
N
O
O
p-MeC6H4
Title i6373.{1H}.1.fid
Solvent CDCl3
Temperature 302.0
Pulse Sequence zg
Number of Scans 1
Receiver Gain 71.8
Relaxation Delay 0.0050
Pulse Width 9.0000
Acquisition Time 1.3518
Spectrometer Frequency 300.13
Spectral Width 6009.6
Lowest Frequency -609.7
Nucleus 1H
Acquired Size 8124
Spectral Size 65536
1.73
1.70
1.84
1.83
2.82
3.00
2.79
3.14
11.5 11.0 10.5 10.0 9.5 9.0 8.5 8.0 7.5 7.0 6.5 6.0 5.5 5.0 4.5 4.0 3.5 3.0 2.5 2.0 1.5 1.0 0.5
ppm

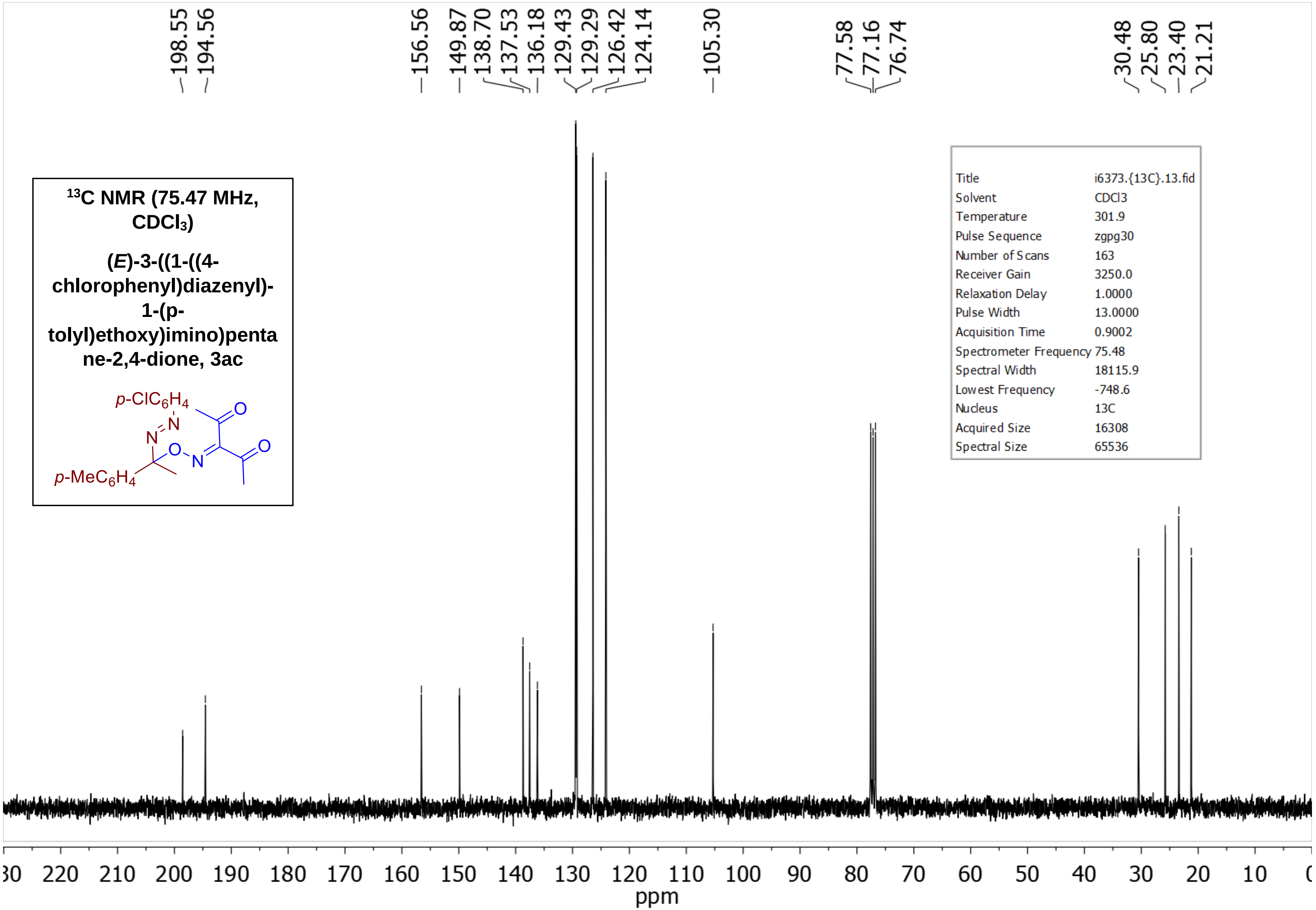
198.55
194.56
156.56
149.87
138.70
137.53
136.18
129.43
129.29
126.42
124.14
105.30
77.58
77.16
76.74
30.48
25.80
23.40
21.21
13C NMR (75.47 MHz, CDCl3)
(E)-3-((1-((4-chlorophenyl)diazenyl)-1-(p-tolyl)ethoxy)imino)pentane-2,4-dione, 3ac
p-ClC6H4
N
N
O
N
O
O
p-MeC6H4
Title i6373.{13C}.13.fid
Solvent CDCl3
Temperature 301.9
Pulse Sequence zgpg30
Number of Scans 163
Receiver Gain 3250.0
Relaxation Delay 1.0000
Pulse Width 13.0000
Acquisition Time 0.9002
Spectrometer Frequency 75.48
Spectral Width 18115.9
Lowest Frequency -748.6
Nucleus 13C
Acquired Size 16308
Spectral Size 65536
220 210 200 190 180 170 160 150 140 130 120 110 100 90 80 70 60 50 40 30 20 10
ppm

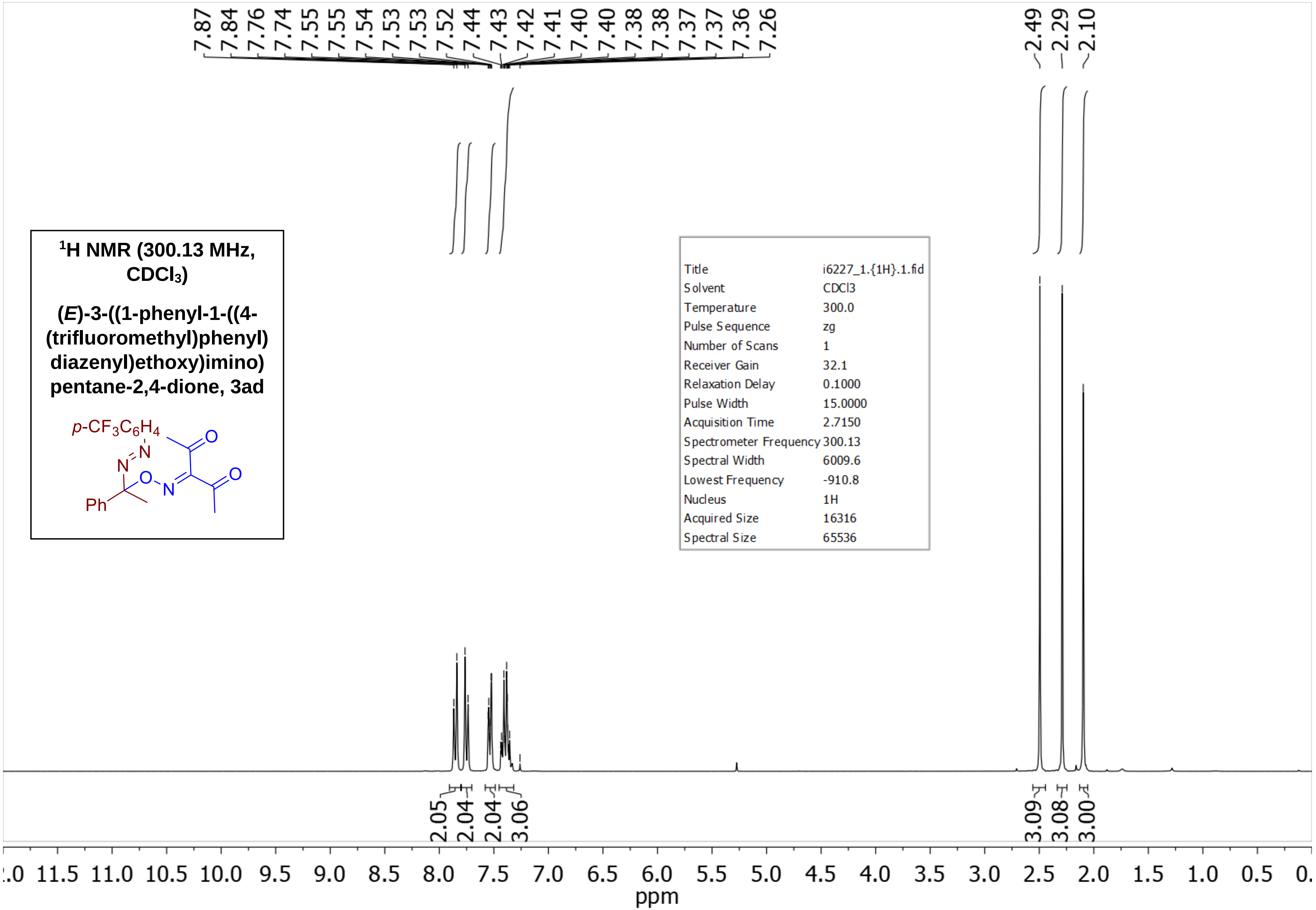

7.87 7.84 7.76 7.74 7.55 7.55 7.54 7.53 7.53 7.52 7.44 7.43 7.42 7.41 7.40 7.40 7.38 7.38 7.37 7.37 7.36 7.26
2.49 2.29 2.10
1H NMR (300.13 MHz, CDCl3)
(E)-3-((1-phenyl-1-((4-(trifluoromethyl)phenyl)diazenyl)ethoxy)imino)pentane-2,4-dione, 3ad
p-CF3C6H4
N
N
O
N
O
O
Ph
Title i6227_1.{1H}.1.fid
Solvent CDCl3
Temperature 300.0
Pulse Sequence zg
Number of Scans 1
Receiver Gain 32.1
Relaxation Delay 0.1000
Pulse Width 15.0000
Acquisition Time 2.7150
Spectrometer Frequency 300.13
Spectral Width 6009.6
Lowest Frequency -910.8
Nucleus 1H
Acquired Size 16316
Spectral Size 65536
2.05 2.04 2.04 3.06
3.09 3.08 3.00
11.5 11.0 10.5 10.0 9.5 9.0 8.5 8.0 7.5 7.0 6.5 6.0 5.5 5.0 4.5 4.0 3.5 3.0 2.5 2.0 1.5 1.0 0.5
ppm

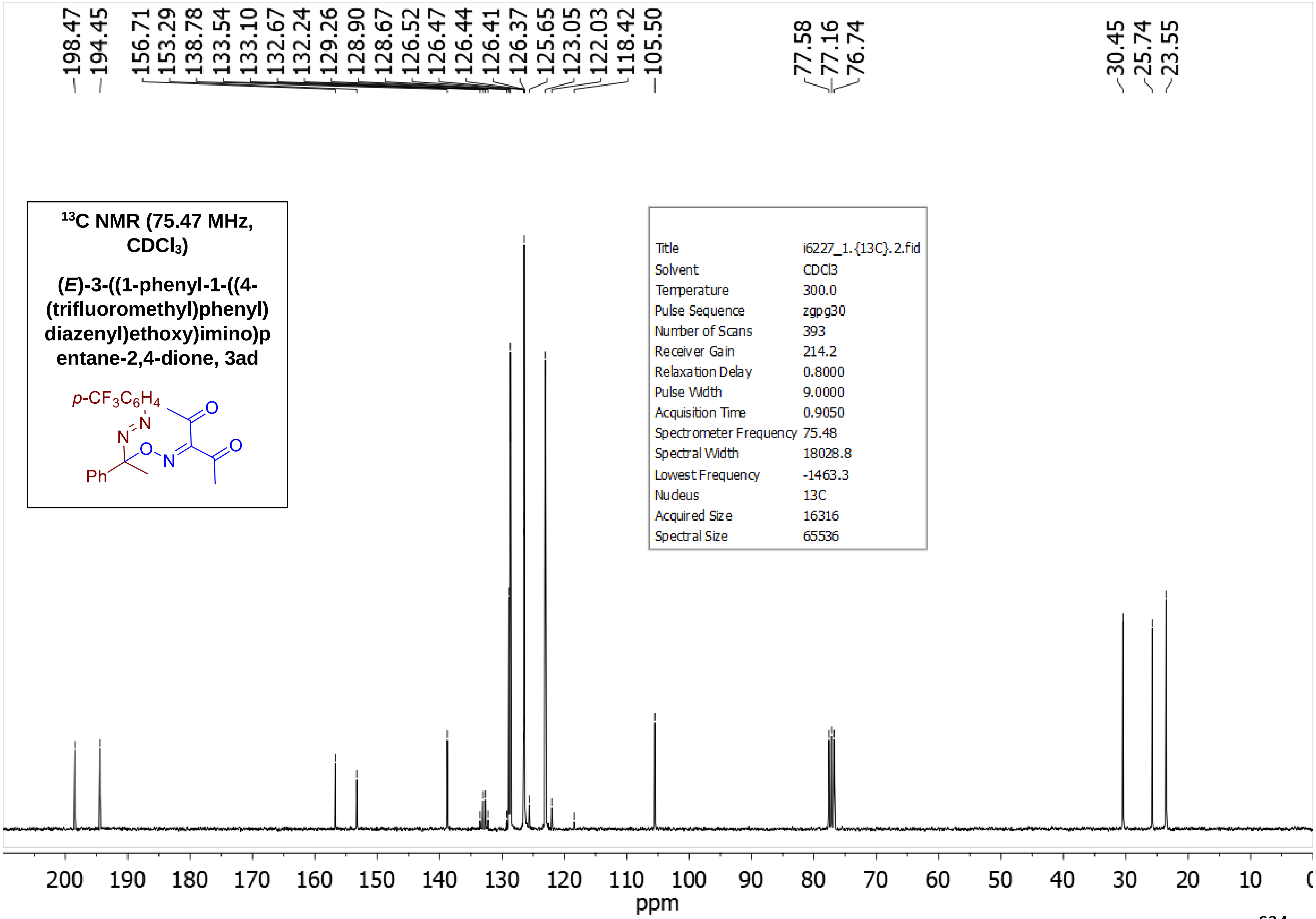
198.47
194.45
156.71
153.29
138.78
133.54
133.10
132.67
132.24
129.26
128.90
128.67
126.52
126.47
126.44
126.41
126.37
125.65
123.05
122.03
118.42
105.50
77.58
77.16
76.74
30.45
25.74
23.55
^{13}C NMR (75.47 MHz, CDCl$_3$)
(*E*)-3-((1-phenyl-1-((4-(trifluoromethyl)phenyl)diazenyl)ethoxy)imino)pentane-2,4-dione, 3ad
p-CF3C6H4
N
N
O
N
O
O
Ph
Title i6227_1.{13C}.2.fid
Solvent CDCl3
Temperature 300.0
Pulse Sequence zgpg30
Number of Scans 393
Receiver Gain 214.2
Relaxation Delay 0.8000
Pulse Width 9.0000
Acquisition Time 0.9050
Spectrometer Frequency 75.48
Spectral Width 18028.8
Lowest Frequency -1463.3
Nucleus 13C
Acquired Size 16316
Spectral Size 65536
200 190 180 170 160 150 140 130 120 110 100 90 80 70 60 50 40 30 20 10
ppm

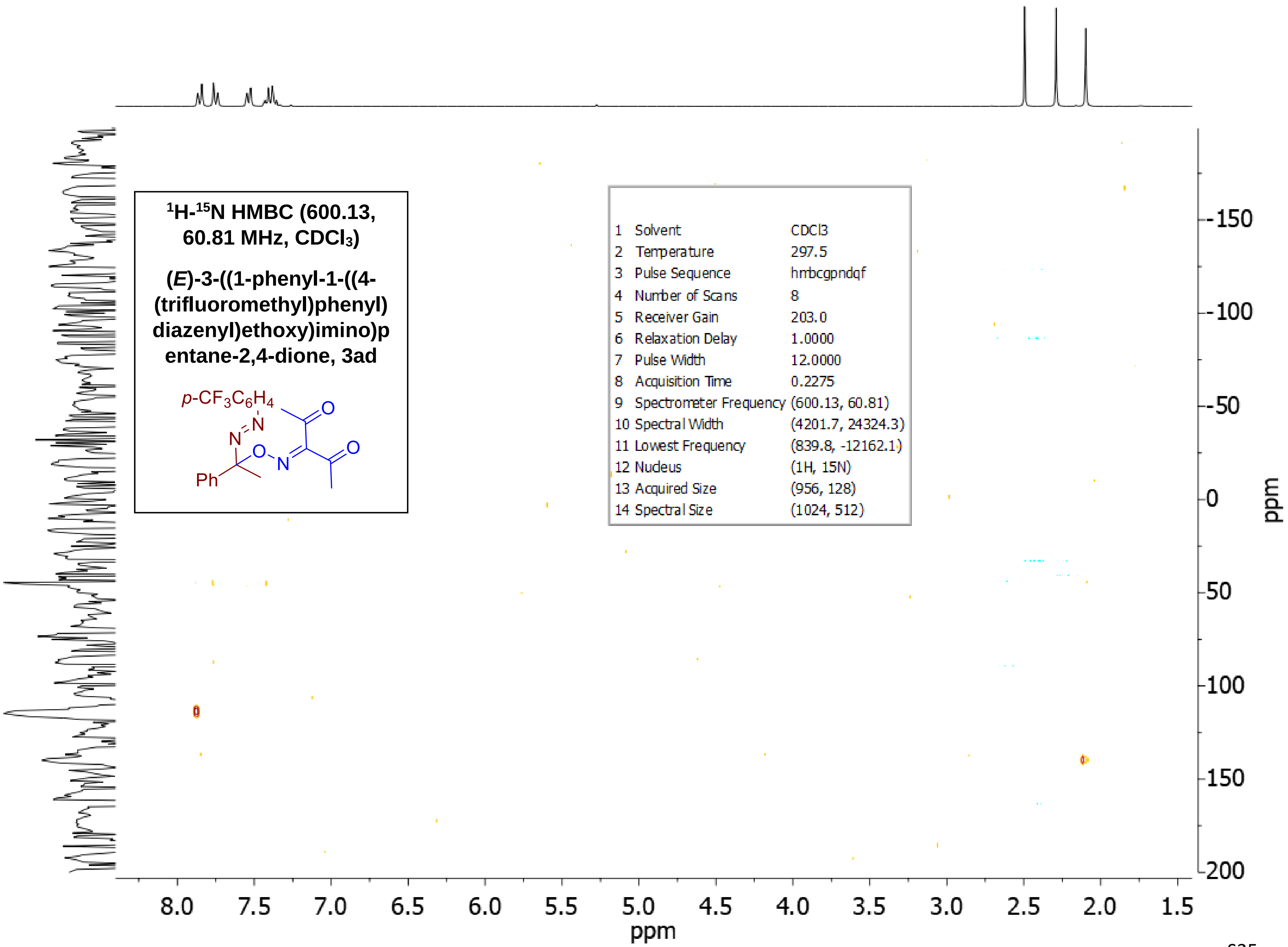
^{1}H-^{15}N HMBC (600.13, 60.81 MHz, CDCl$_3$)
(*E*)-3-((1-phenyl-1-((4-(trifluoromethyl)phenyl)diazenyl)ethoxy)imino)pentane-2,4-dione, 3ad
p-CF$_3$C$_6$H$_4$
N
N
O
N
O
O
Ph
1 Solvent CDCl3
2 Temperature 297.5
3 Pulse Sequence hmbcgpndqf
4 Number of Scans 8
5 Receiver Gain 203.0
6 Relaxation Delay 1.0000
7 Pulse Width 12.0000
8 Acquisition Time 0.2275
9 Spectrometer Frequency (600.13, 60.81)
10 Spectral Width (4201.7, 24324.3)
11 Lowest Frequency (839.8, -12162.1)
12 Nucleus (1H, 15N)
13 Acquired Size (956, 128)
14 Spectral Size (1024, 512)
-150
-100
-50
0
50
100
150
200
ppm
8.0
7.5
7.0
6.5
6.0
5.5
5.0
4.5
4.0
3.5
3.0
2.5
2.0
1.5
ppm

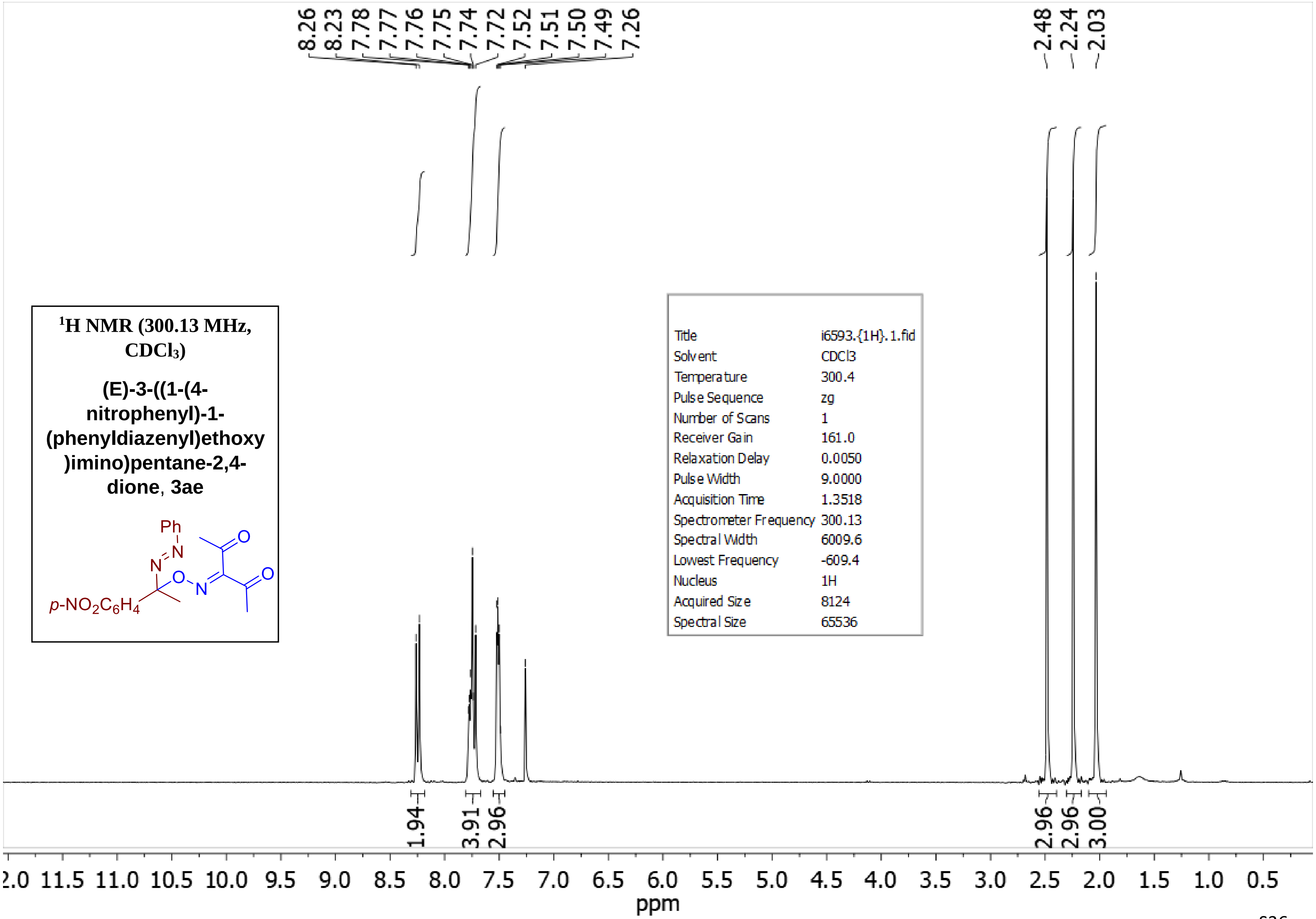

8.26
8.23
7.78
7.77
7.76
7.75
7.74
7.72
7.52
7.51
7.50
7.49
7.26
2.48
2.24
2.03
1H NMR (300.13 MHz, CDCl3)
(E)-3-((1-(4-nitrophenyl)-1-(phenyldiazenyl)ethoxy)imino)pentane-2,4-dione, 3ae
Ph
N
N
O
N
O
O
p-NO2C6H4
Title i6593.{1H}.1.fid
Solvent CDCl3
Temperature 300.4
Pulse Sequence zg
Number of Scans 1
Receiver Gain 161.0
Relaxation Delay 0.0050
Pulse Width 9.0000
Acquisition Time 1.3518
Spectrometer Frequency 300.13
Spectral Width 6009.6
Lowest Frequency -609.4
Nucleus 1H
Acquired Size 8124
Spectral Size 65536
1.94
3.91
2.96
2.96
2.96
3.00
12.0 11.5 11.0 10.5 10.0 9.5 9.0 8.5 8.0 7.5 7.0 6.5 6.0 5.5 5.0 4.5 4.0 3.5 3.0 2.5 2.0 1.5 1.0 0.5
ppm

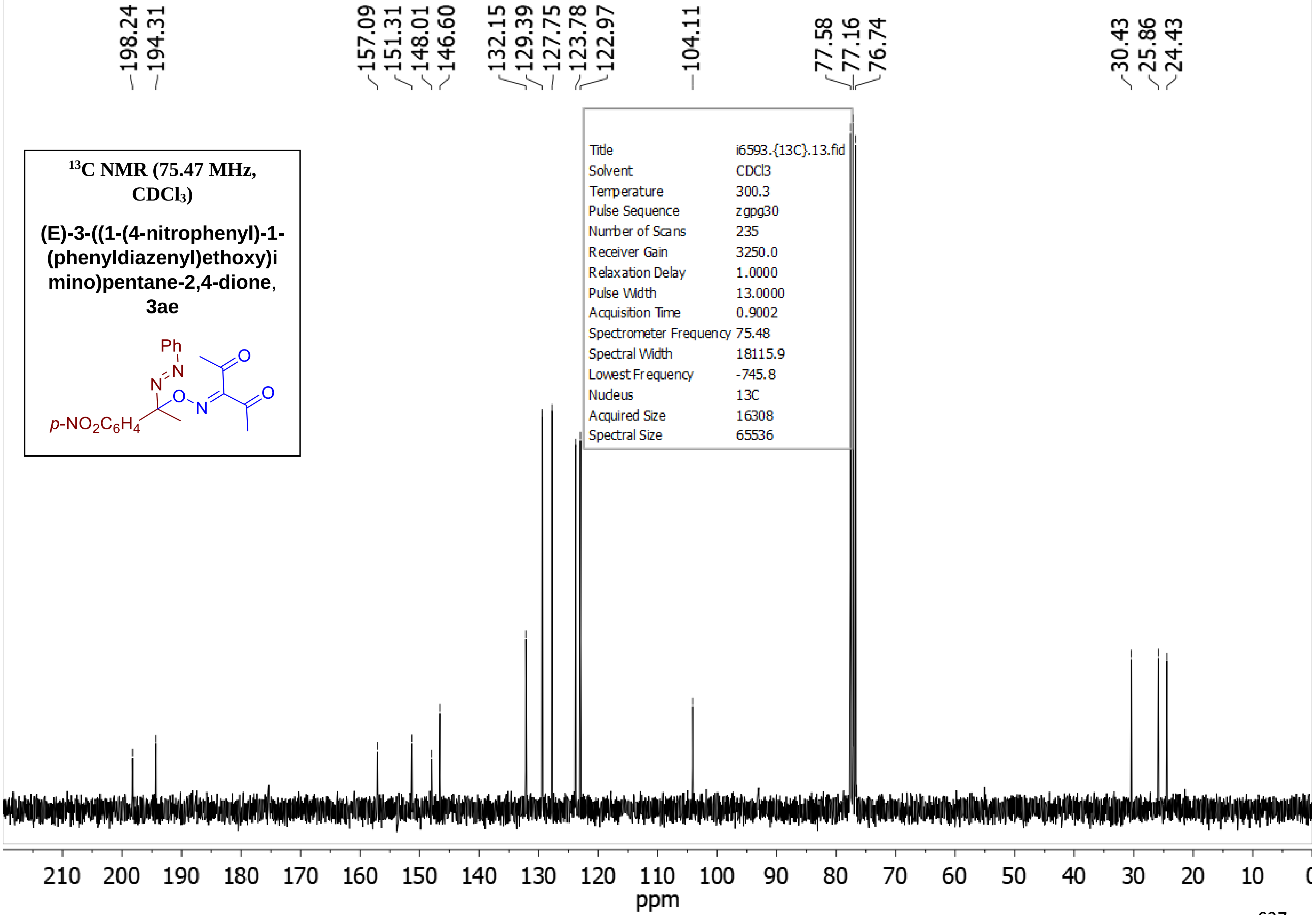
198.24
194.31
157.09
151.31
148.01
146.60
132.15
129.39
127.75
123.78
122.97
104.11
77.58
77.16
76.74
30.43
25.86
24.43
^{13}C NMR (75.47 MHz, $CDCl_3$)
(E)-3-((1-(4-nitrophenyl)-1-(phenyldiazenyl)ethoxy)imino)pentane-2,4-dione, 3ae
Ph
N
N
O
N
O
O
p-$NO_2C_6H_4$
Title i6593.{13C}.13.fid
Solvent CDCl3
Temperature 300.3
Pulse Sequence zgpg30
Number of Scans 235
Receiver Gain 3250.0
Relaxation Delay 1.0000
Pulse Width 13.0000
Acquisition Time 0.9002
Spectrometer Frequency 75.48
Spectral Width 18115.9
Lowest Frequency -745.8
Nucleus 13C
Acquired Size 16308
Spectral Size 65536
210 200 190 180 170 160 150 140 130 120 110 100 90 80 70 60 50 40 30 20 10 0
ppm

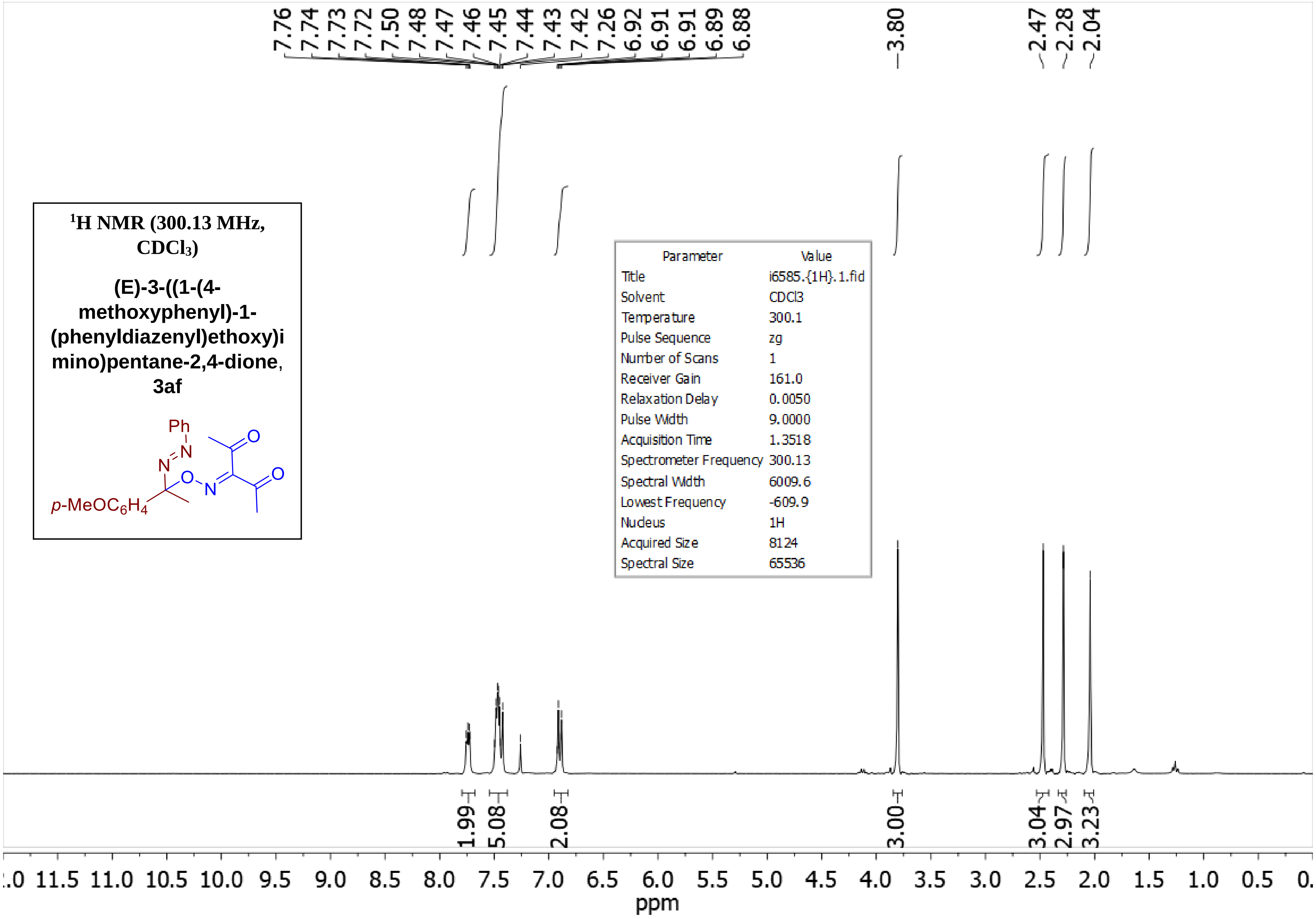

7.76 7.74 7.73 7.72 7.50 7.48 7.47 7.46 7.45 7.44 7.43 7.42 7.26 6.92 6.91 6.91 6.89 6.88
3.80
2.47 2.28 2.04
1H NMR (300.13 MHz, CDCl3)
(E)-3-((1-(4-methoxyphenyl)-1-(phenyldiazenyl)ethoxy)imino)pentane-2,4-dione, 3af
Ph
N
N
O
N
O
O
p-MeOC6H4
Parameter Value
Title i6585.{1H}.1.fid
Solvent CDCl3
Temperature 300.1
Pulse Sequence zg
Number of Scans 1
Receiver Gain 161.0
Relaxation Delay 0.0050
Pulse Width 9.0000
Acquisition Time 1.3518
Spectrometer Frequency 300.13
Spectral Width 6009.6
Lowest Frequency -609.9
Nucleus 1H
Acquired Size 8124
Spectral Size 65536
1.99 5.08 2.08 3.00 3.04 2.97 3.23
11.5 11.0 10.5 10.0 9.5 9.0 8.5 8.0 7.5 7.0 6.5 6.0 5.5 5.0 4.5 4.0 3.5 3.0 2.5 2.0 1.5 1.0 0.5
ppm

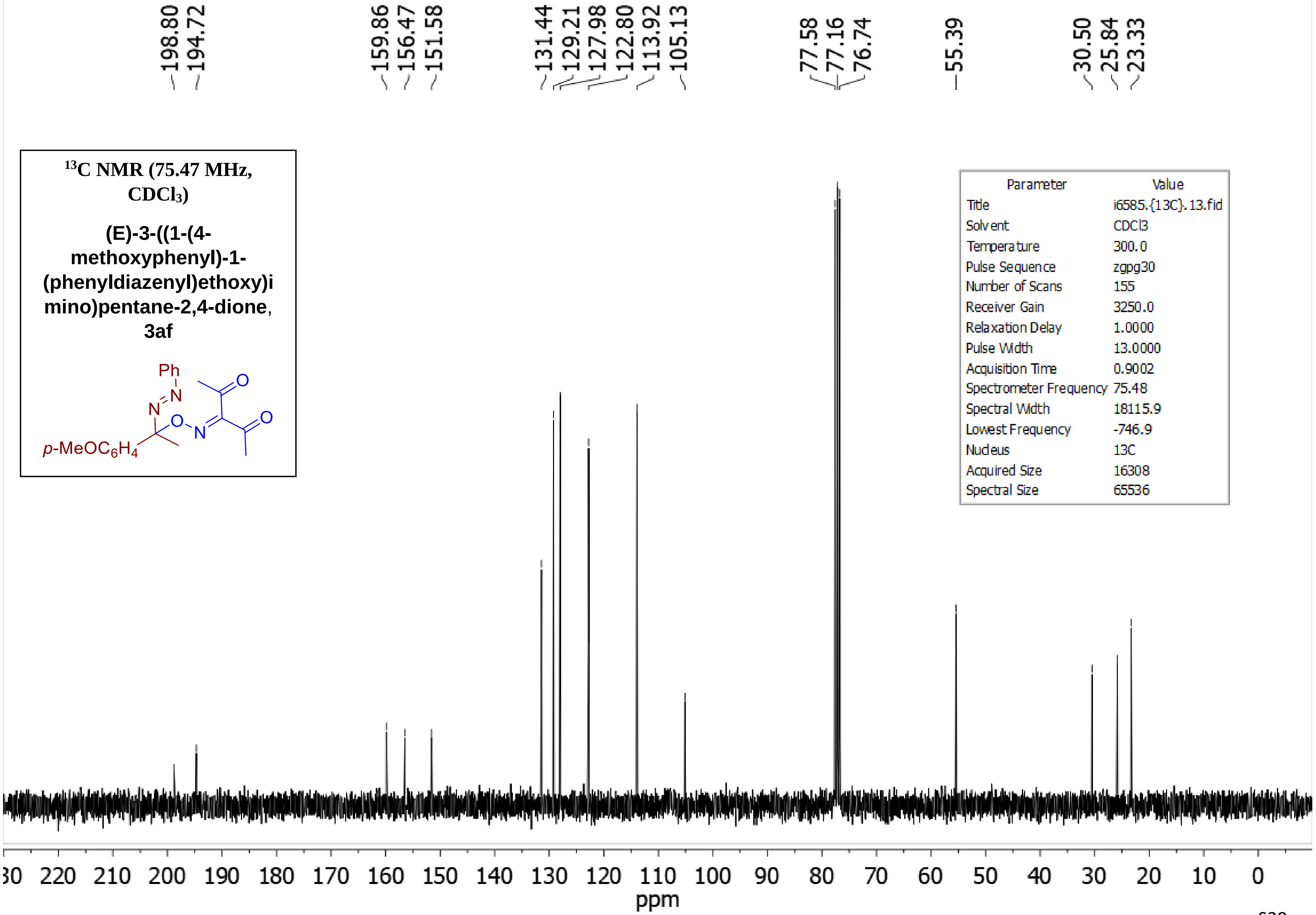

^{13}C NMR (75.47 MHz, $CDCl_3$)

(E)-3-((1-(4-methoxyphenyl)-1-(phenyldiazenyl)ethoxy)imino)pentane-2,4-dione, 3af

Parameter	Value
Title	i6585.{13C}.13.fid
Solvent	CDCl3
Temperature	300.0
Pulse Sequence	zgpg30
Number of Scans	155
Receiver Gain	3250.0
Relaxation Delay	1.0000
Pulse Width	13.0000
Acquisition Time	0.9002
Spectrometer Frequency	75.48
Spectral Width	18115.9
Lowest Frequency	-746.9
Nucleus	13C
Acquired Size	16308
Spectral Size	65536

^{1}H NMR (300.13 MHz, $CDCl_3$)

(E)-3-((1-(4-bromophenyl)-1-(phenyldiazenyl)ethoxy)imino)pentane-2,4-dione, 3ag

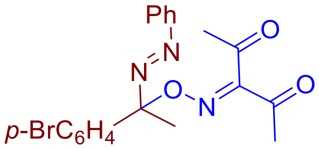

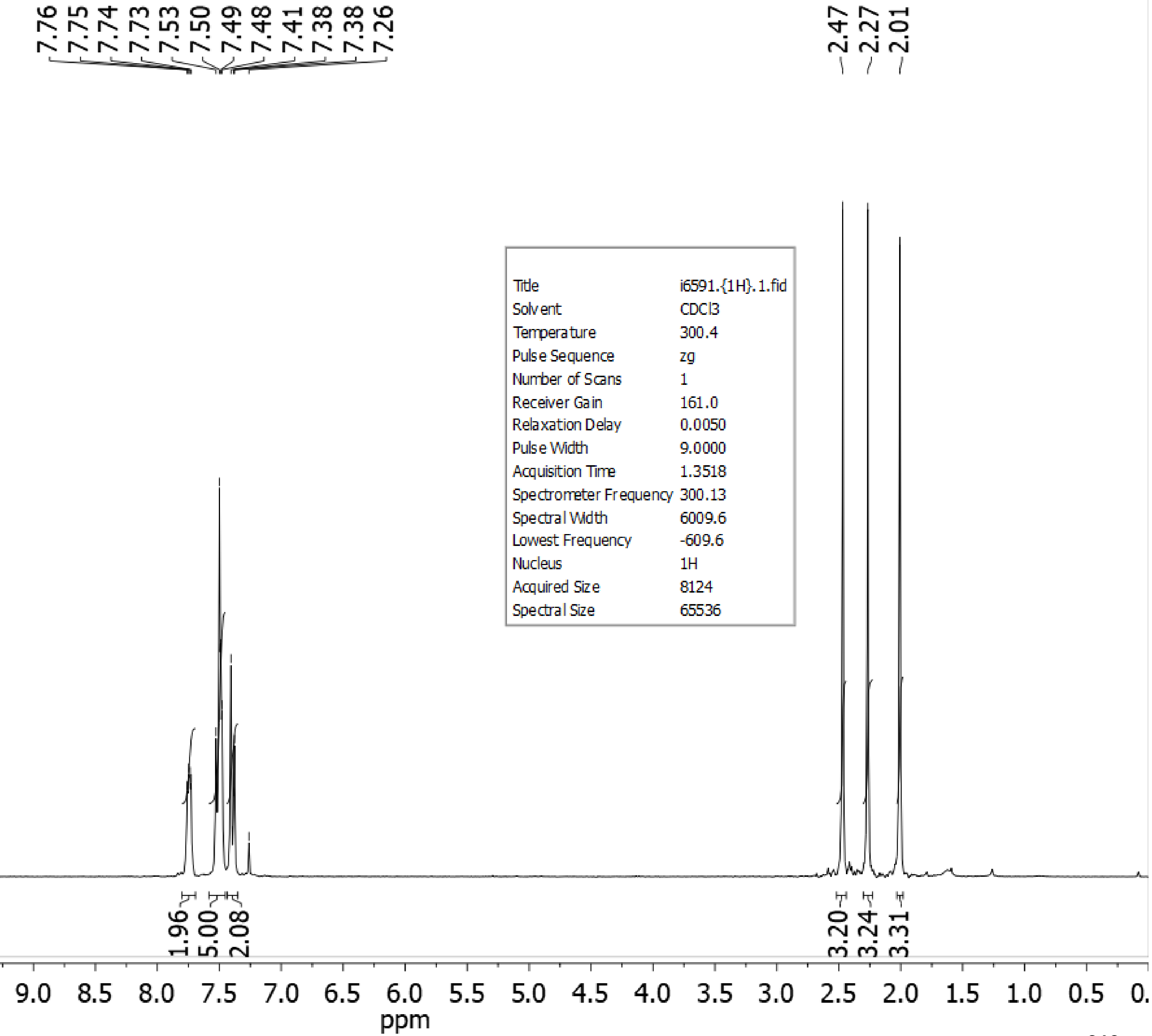

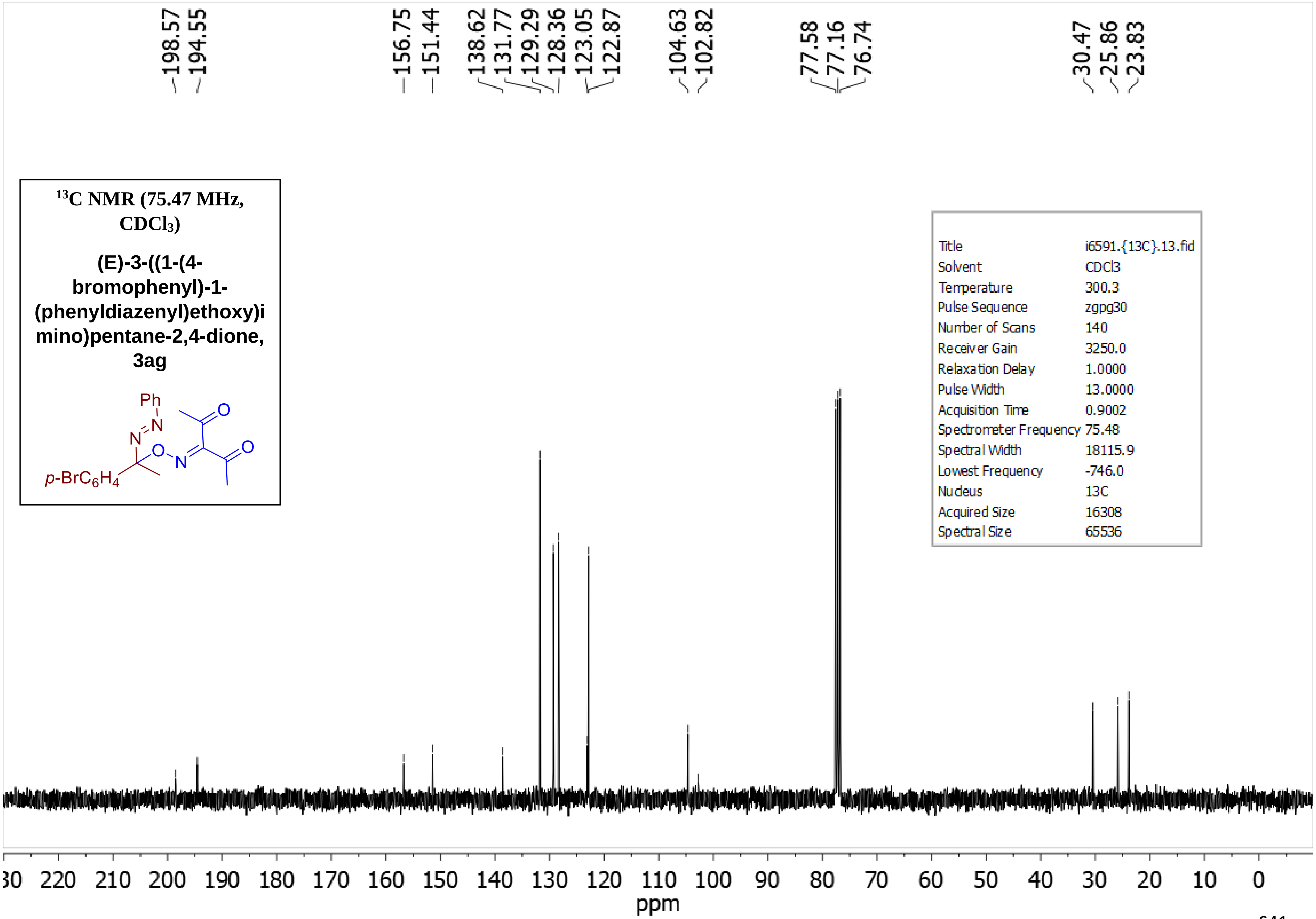
198.57
194.55
156.75
151.44
138.62
131.77
129.29
128.36
123.05
122.87
104.63
102.82
77.58
77.16
76.74
30.47
25.86
23.83
^{13}C NMR (75.47 MHz, $CDCl_3$)
(E)-3-((1-(4-bromophenyl)-1-(phenyldiazenyl)ethoxy)imino)pentane-2,4-dione, 3ag
Ph
N
N
O
N
O
O
p-BrC_6H_4
Title i6591.{13C}.13.fid
Solvent CDCl3
Temperature 300.3
Pulse Sequence zgpg30
Number of Scans 140
Receiver Gain 3250.0
Relaxation Delay 1.0000
Pulse Width 13.0000
Acquisition Time 0.9002
Spectrometer Frequency 75.48
Spectral Width 18115.9
Lowest Frequency -746.0
Nucleus 13C
Acquired Size 16308
Spectral Size 65536
30 220 210 200 190 180 170 160 150 140 130 120 110 100 90 80 70 60 50 40 30 20 10 0
ppm

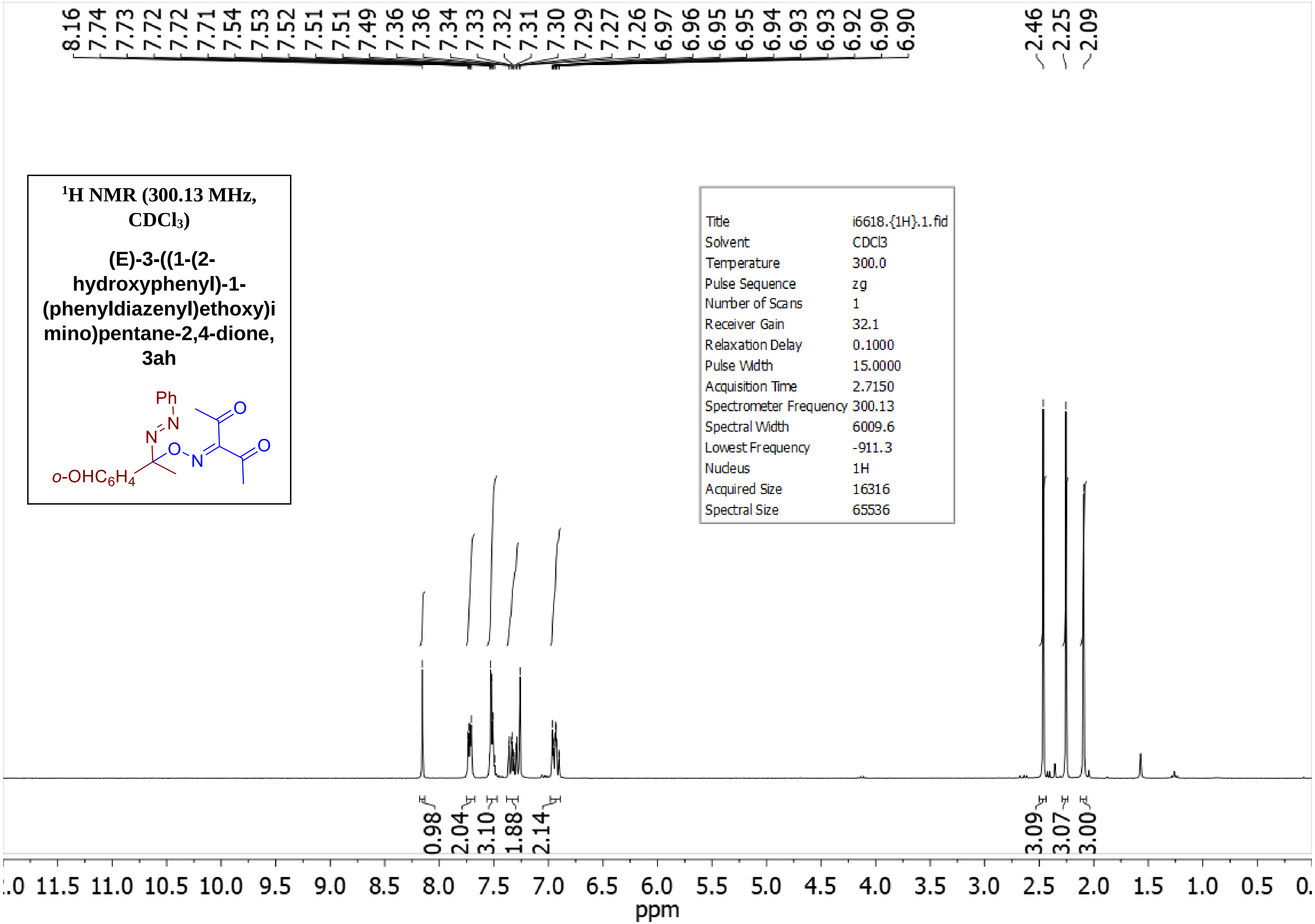

^{1}H NMR (300.13 MHz, CDCl3)
(E)-3-((1-(2-hydroxyphenyl)-1-(phenyldiazenyl)ethoxy)imino)pentane-2,4-dione, 3ah
Ph
N
N
O
N
O
O
o-OHC6H4
8.16 7.74 7.73 7.72 7.72 7.71 7.54 7.53 7.52 7.51 7.51 7.49 7.36 7.36 7.34 7.33 7.32 7.31 7.30 7.29 7.27 7.26 6.97 6.96 6.95 6.95 6.94 6.93 6.93 6.92 6.90 6.90
2.46 2.25 2.09
Title i6618.{1H}.1.fid
Solvent CDCl3
Temperature 300.0
Pulse Sequence zg
Number of Scans 1
Receiver Gain 32.1
Relaxation Delay 0.1000
Pulse Width 15.0000
Acquisition Time 2.7150
Spectrometer Frequency 300.13
Spectral Width 6009.6
Lowest Frequency -911.3
Nucleus 1H
Acquired Size 16316
Spectral Size 65536
0.98 2.04 3.10 1.88 2.14
3.09 3.07 3.00
.0 11.5 11.0 10.5 10.0 9.5 9.0 8.5 8.0 7.5 7.0 6.5 6.0 5.5 5.0 4.5 4.0 3.5 3.0 2.5 2.0 1.5 1.0 0.5 0.
ppm

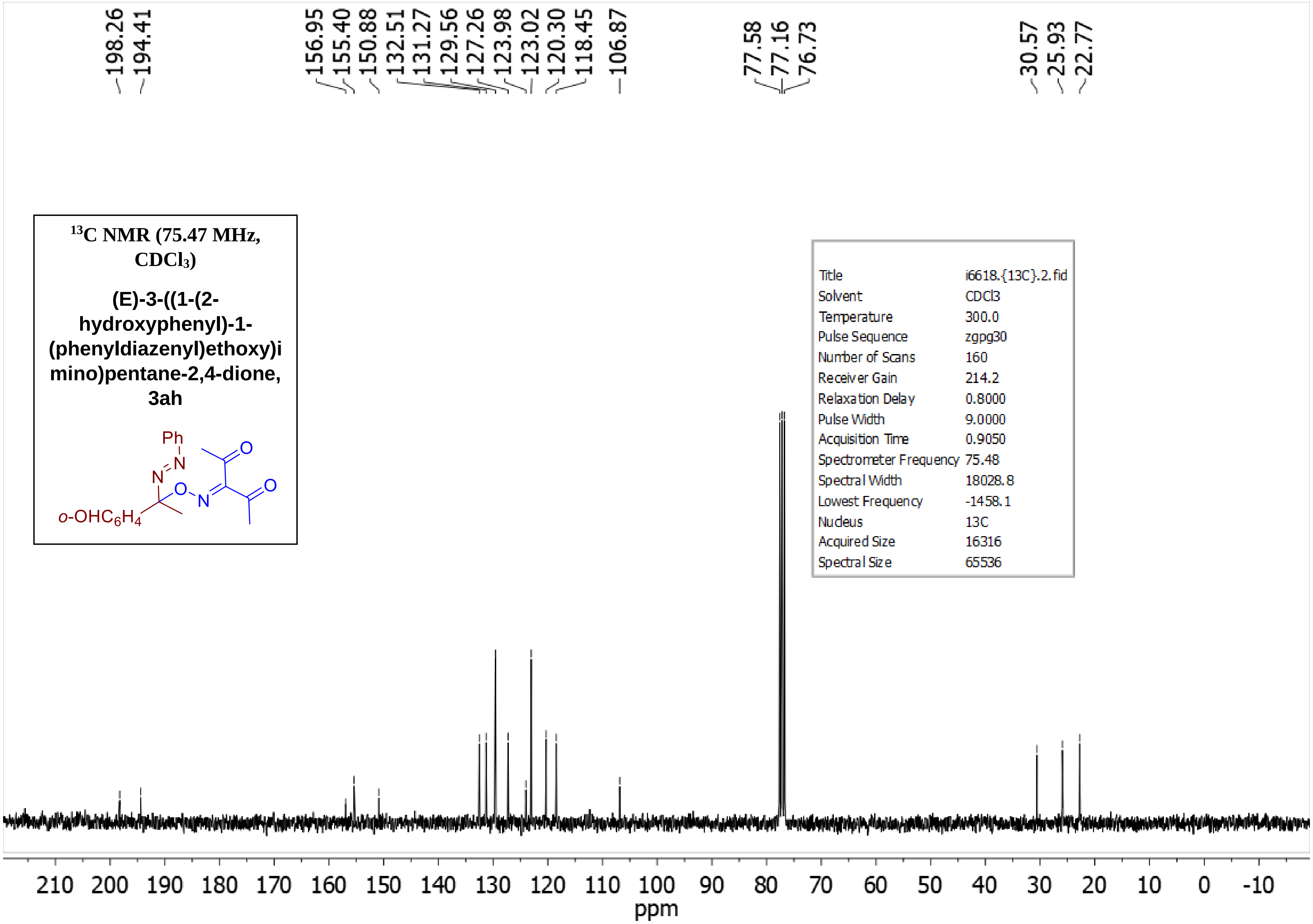
198.26
194.41
156.95
155.40
150.88
132.51
131.27
129.56
127.26
123.98
123.02
120.30
118.45
106.87
77.58
77.16
76.73
30.57
25.93
22.77
13C NMR (75.47 MHz, CDCl3)
(E)-3-((1-(2-hydroxyphenyl)-1-(phenyldiazenyl)ethoxy)imino)pentane-2,4-dione, 3ah
Ph
N
N
O
N
O
O
o-OHC6H4
Title i6618.{13C}.2.fid
Solvent CDCl3
Temperature 300.0
Pulse Sequence zgpg30
Number of Scans 160
Receiver Gain 214.2
Relaxation Delay 0.8000
Pulse Width 9.0000
Acquisition Time 0.9050
Spectrometer Frequency 75.48
Spectral Width 18028.8
Lowest Frequency -1458.1
Nucleus 13C
Acquired Size 16316
Spectral Size 65536
210 200 190 180 170 160 150 140 130 120 110 100 90 80 70 60 50 40 30 20 10 0 -10
ppm

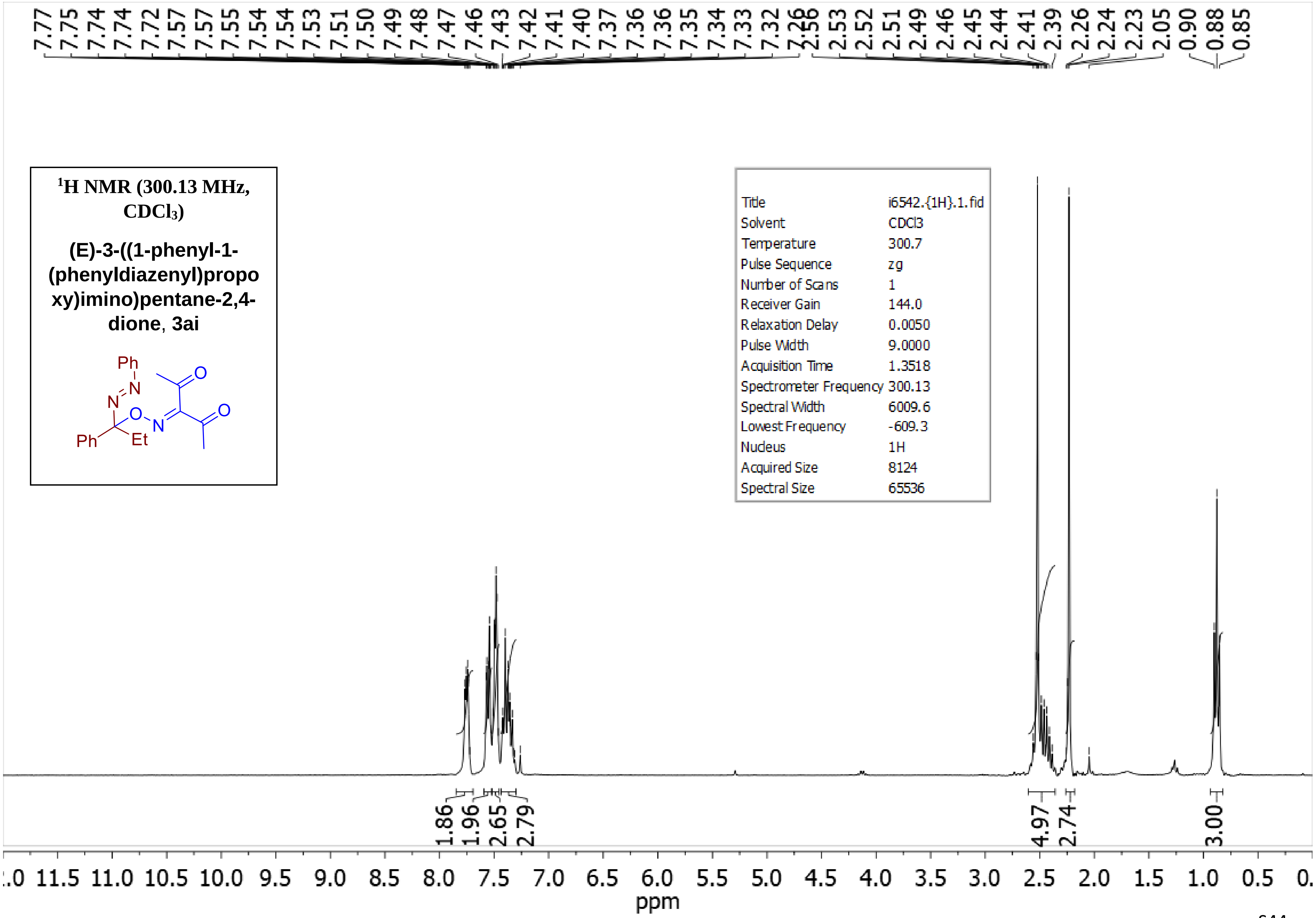

1H NMR (300.13 MHz, CDCl3)
(E)-3-((1-phenyl-1-(phenyldiazenyl)propoxy)imino)pentane-2,4-dione, 3ai
Ph
N
N
O
N
O
O
Ph
Et
Title i6542.{1H}.1.fid
Solvent CDCl3
Temperature 300.7
Pulse Sequence zg
Number of Scans 1
Receiver Gain 144.0
Relaxation Delay 0.0050
Pulse Width 9.0000
Acquisition Time 1.3518
Spectrometer Frequency 300.13
Spectral Width 6009.6
Lowest Frequency -609.3
Nucleus 1H
Acquired Size 8124
Spectral Size 65536
1.86
1.96
2.65
2.79
4.97
2.74
3.00
ppm

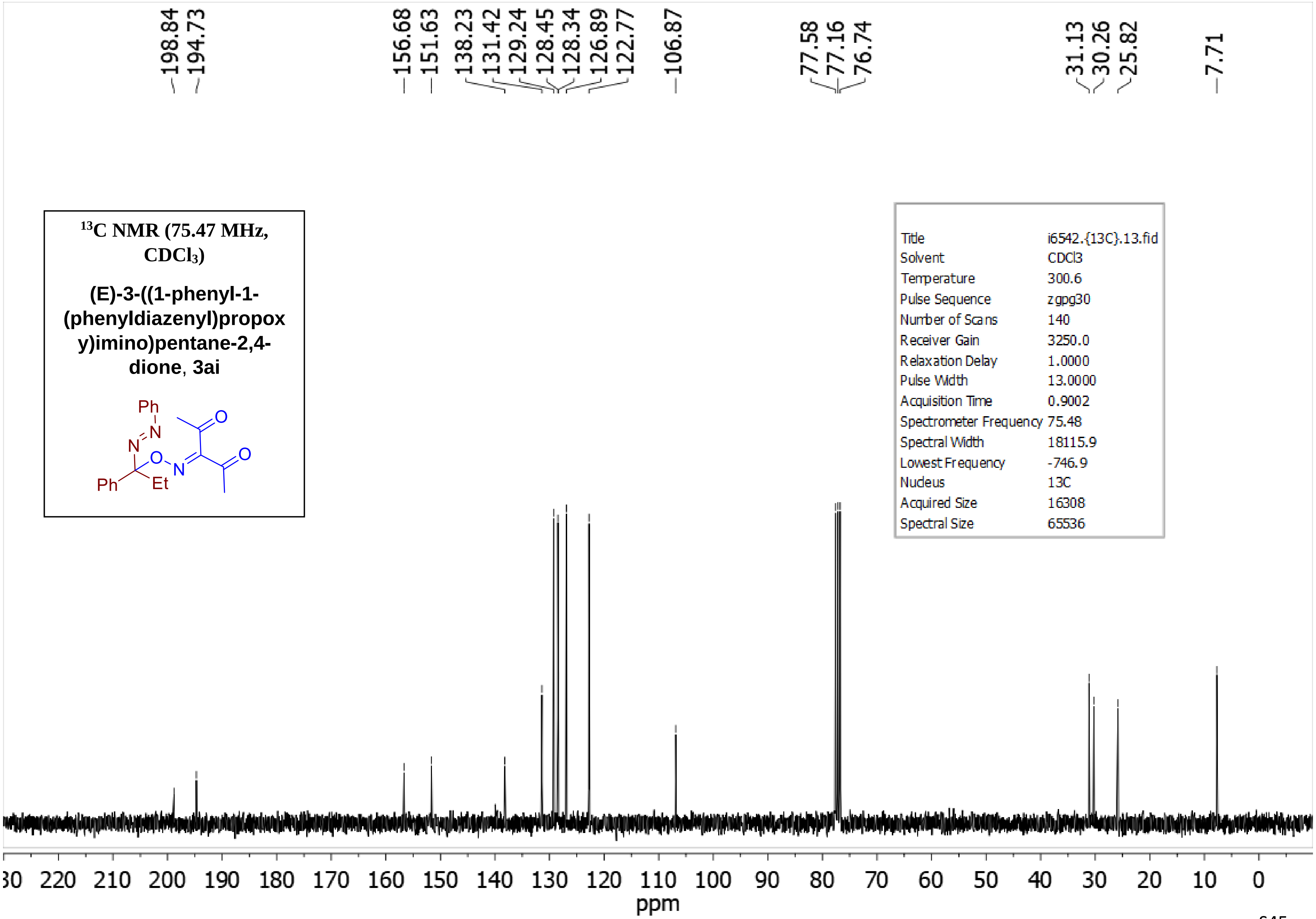

198.84
194.73
156.68
151.63
138.23
131.42
129.24
128.45
128.34
126.89
122.77
106.87
77.58
77.16
76.74
31.13
30.26
25.82
7.71
13C NMR (75.47 MHz, CDCl3)
(E)-3-((1-phenyl-1-(phenyldiazenyl)propoxy)imino)pentane-2,4-dione, 3ai
Ph
N
N
O
N
O
O
Ph
Et
Title i6542.{13C}.13.fid
Solvent CDCl3
Temperature 300.6
Pulse Sequence zgpg30
Number of Scans 140
Receiver Gain 3250.0
Relaxation Delay 1.0000
Pulse Width 13.0000
Acquisition Time 0.9002
Spectrometer Frequency 75.48
Spectral Width 18115.9
Lowest Frequency -746.9
Nucleus 13C
Acquired Size 16308
Spectral Size 65536
220 210 200 190 180 170 160 150 140 130 120 110 100 90 80 70 60 50 40 30 20 10 0
ppm

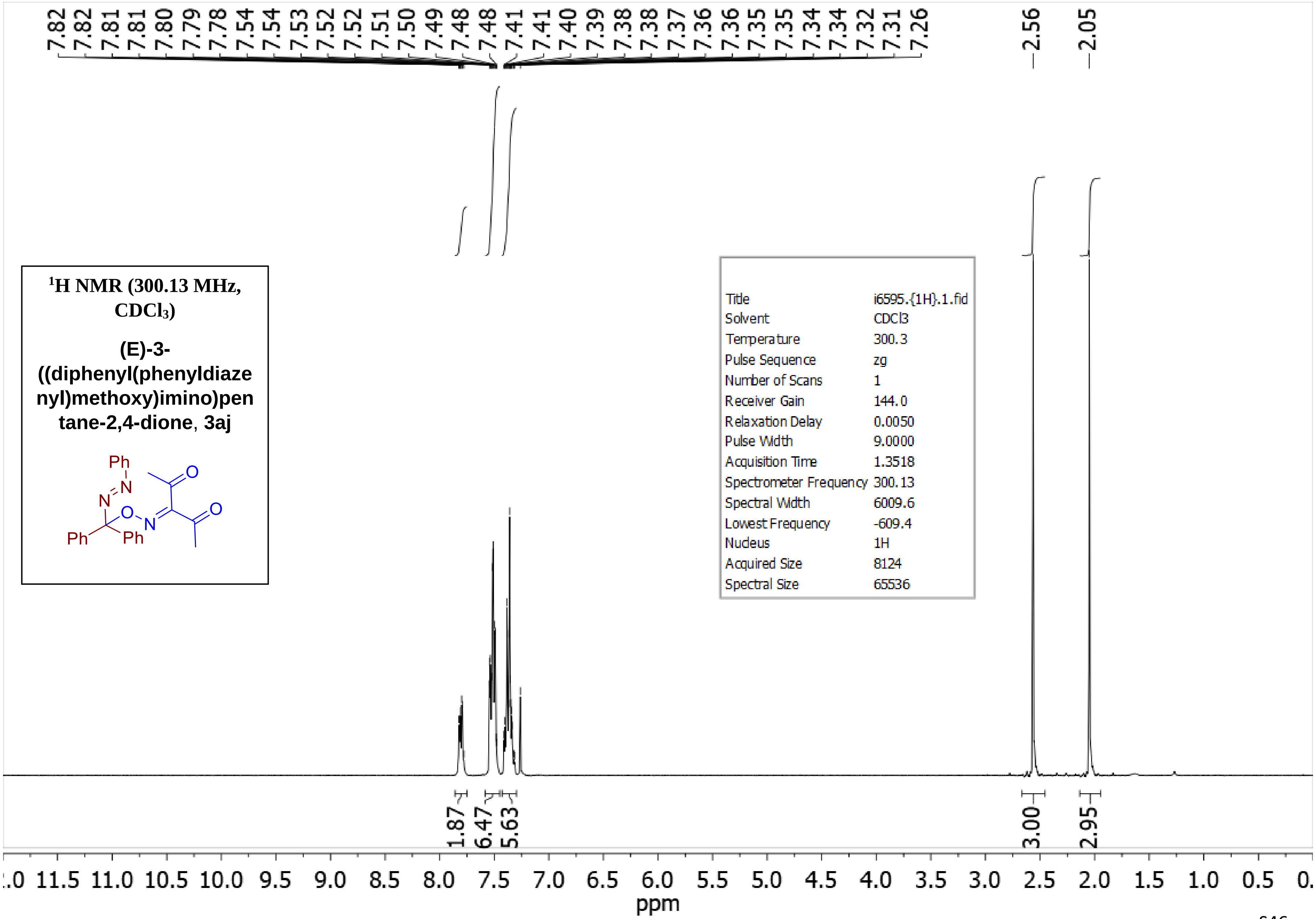
7.82 7.82 7.81 7.81 7.80 7.79 7.78 7.54 7.54 7.53 7.52 7.52 7.51 7.50 7.49 7.48 7.48 7.41 7.41 7.40 7.39 7.38 7.38 7.37 7.36 7.36 7.35 7.35 7.34 7.34 7.32 7.31 7.26
2.56
2.05
1H NMR (300.13 MHz, CDCl3)
(E)-3-((diphenyl(phenyldiazenyl)methoxy)imino)pentane-2,4-dione, 3aj
Ph
N
N
O
N
O
O
Ph
Ph
Title i6595.{1H}.1.fid
Solvent CDCl3
Temperature 300.3
Pulse Sequence zg
Number of Scans 1
Receiver Gain 144.0
Relaxation Delay 0.0050
Pulse Width 9.0000
Acquisition Time 1.3518
Spectrometer Frequency 300.13
Spectral Width 6009.6
Lowest Frequency -609.4
Nucleus 1H
Acquired Size 8124
Spectral Size 65536
1.87
6.47
5.63
3.00
2.95
11.5 11.0 10.5 10.0 9.5 9.0 8.5 8.0 7.5 7.0 6.5 6.0 5.5 5.0 4.5 4.0 3.5 3.0 2.5 2.0 1.5 1.0 0.5
ppm

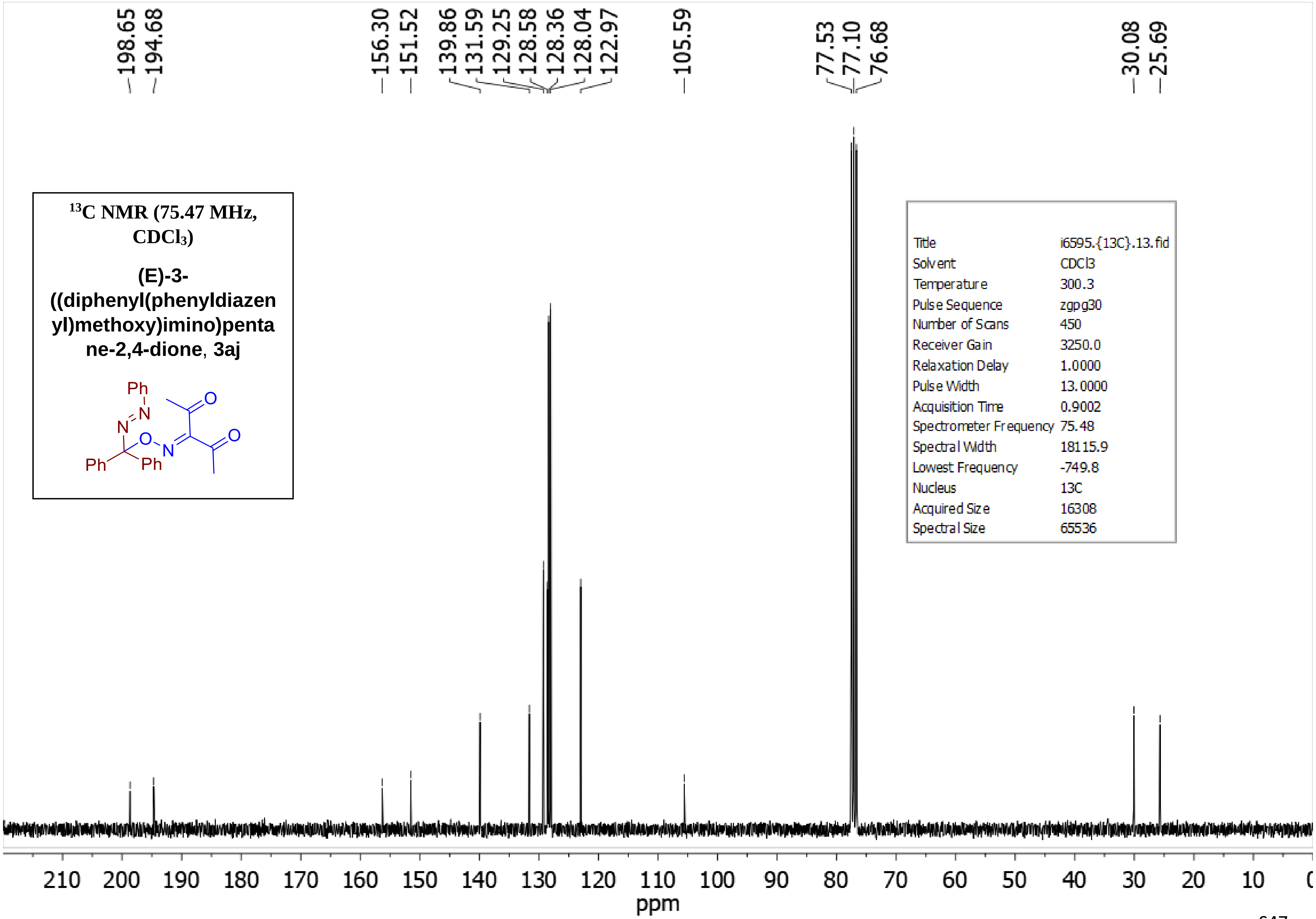
198.65
194.68
156.30
151.52
139.86
131.59
129.25
128.58
128.36
128.04
122.97
105.59
77.53
77.10
76.68
30.08
25.69
^{13}C NMR (75.47 MHz, $CDCl_3$)
(E)-3-((diphenyl(phenyldiazenyl)methoxy)imino)pentane-2,4-dione, 3aj
Ph
N
N
O
N
O
O
Ph
Ph
Title i6595.{13C}.13.fid
Solvent CDCl3
Temperature 300.3
Pulse Sequence zgpg30
Number of Scans 450
Receiver Gain 3250.0
Relaxation Delay 1.0000
Pulse Width 13.0000
Acquisition Time 0.9002
Spectrometer Frequency 75.48
Spectral Width 18115.9
Lowest Frequency -749.8
Nucleus 13C
Acquired Size 16308
Spectral Size 65536
210 200 190 180 170 160 150 140 130 120 110 100 90 80 70 60 50 40 30 20 10 0
ppm

^{1}H NMR (300.13 MHz, $CDCl_3$)

(E)-3-((1-phenyl-1-(pyridin-2-yldiazenyl)ethoxy)imino)pentane-2,4-dione

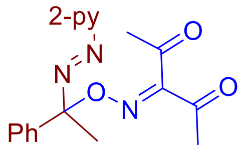

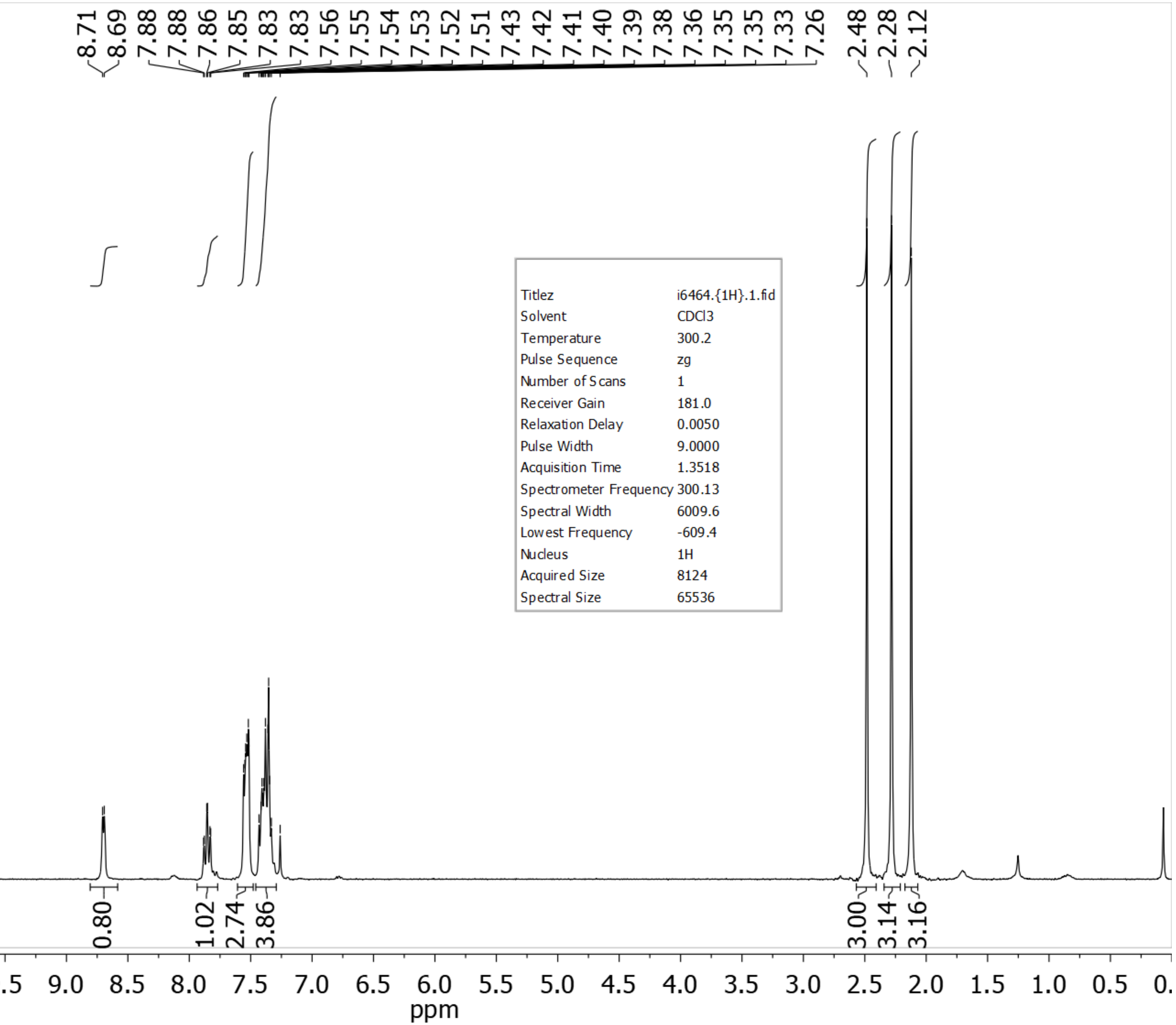

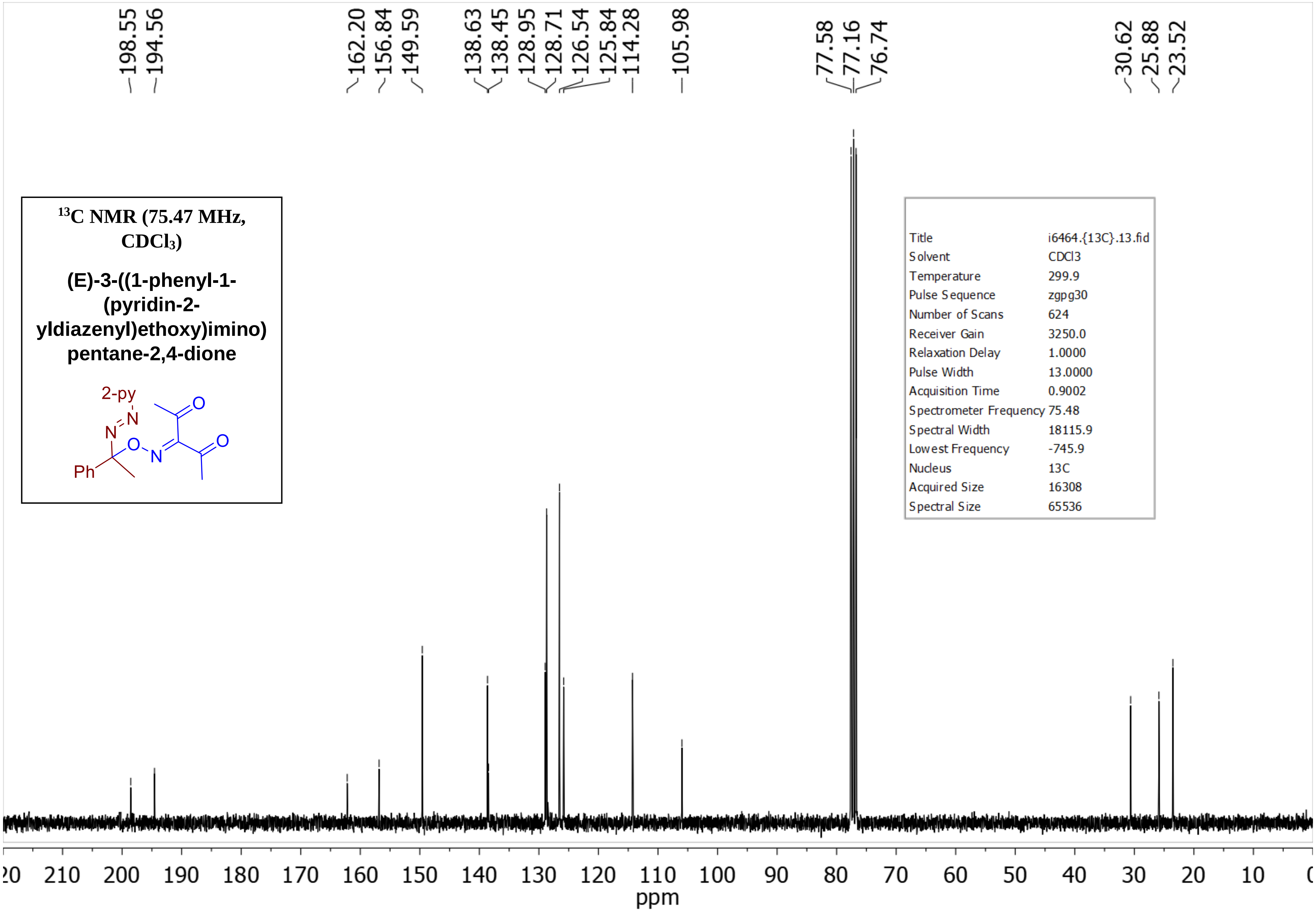

198.55
194.56
162.20
156.84
149.59
138.63
138.45
128.95
128.71
126.54
125.84
114.28
105.98
77.58
77.16
76.74
30.62
25.88
23.52
^{13}C NMR (75.47 MHz, CDCl$_3$)
(E)-3-((1-phenyl-1-(pyridin-2-yldiazenyl)ethoxy)imino)pentane-2,4-dione
2-py
N
N
O
N
O
O
Ph
Title i6464.{13C}.13.fid
Solvent CDCl3
Temperature 299.9
Pulse Sequence zgpg30
Number of Scans 624
Receiver Gain 3250.0
Relaxation Delay 1.0000
Pulse Width 13.0000
Acquisition Time 0.9002
Spectrometer Frequency 75.48
Spectral Width 18115.9
Lowest Frequency -745.9
Nucleus 13C
Acquired Size 16308
Spectral Size 65536
210 200 190 180 170 160 150 140 130 120 110 100 90 80 70 60 50 40 30 20 10
ppm

^{1}H NMR (300.13 MHz, $CDCl_3$)

3-((((Z)-1,4-diphenyl-1-((E)-phenyldiazenyl)but-3-en-1-yl)oxy)imino)pentane-2,4-dione, 3al

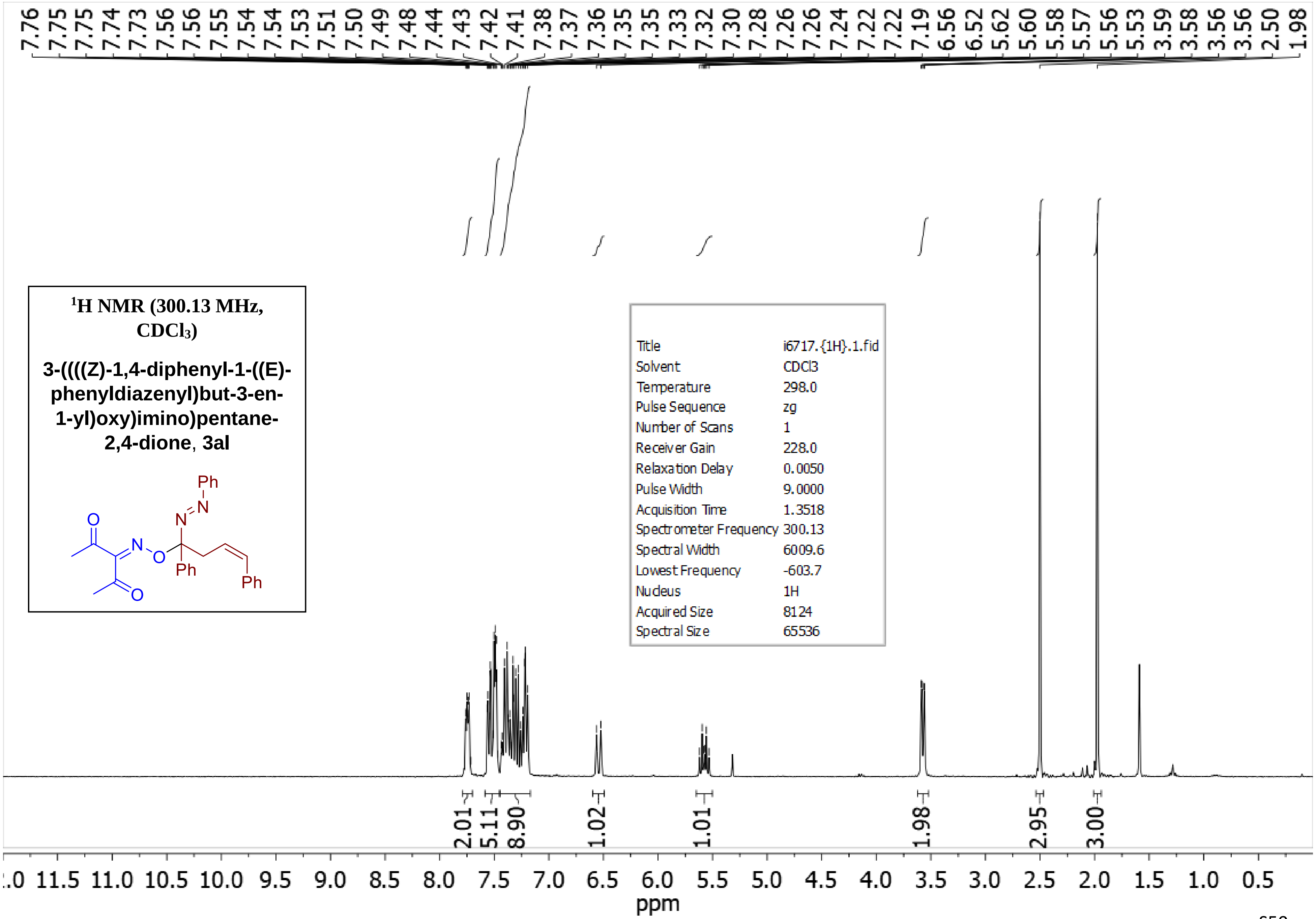

Title	i6717.{1H}.1.fid
Solvent	CDCl3
Temperature	298.0
Pulse Sequence	zg
Number of Scans	1
Receiver Gain	228.0
Relaxation Delay	0.0050
Pulse Width	9.0000
Acquisition Time	1.3518
Spectrometer Frequency	300.13
Spectral Width	6009.6
Lowest Frequency	-603.7
Nucleus	1H
Acquired Size	8124
Spectral Size	65536

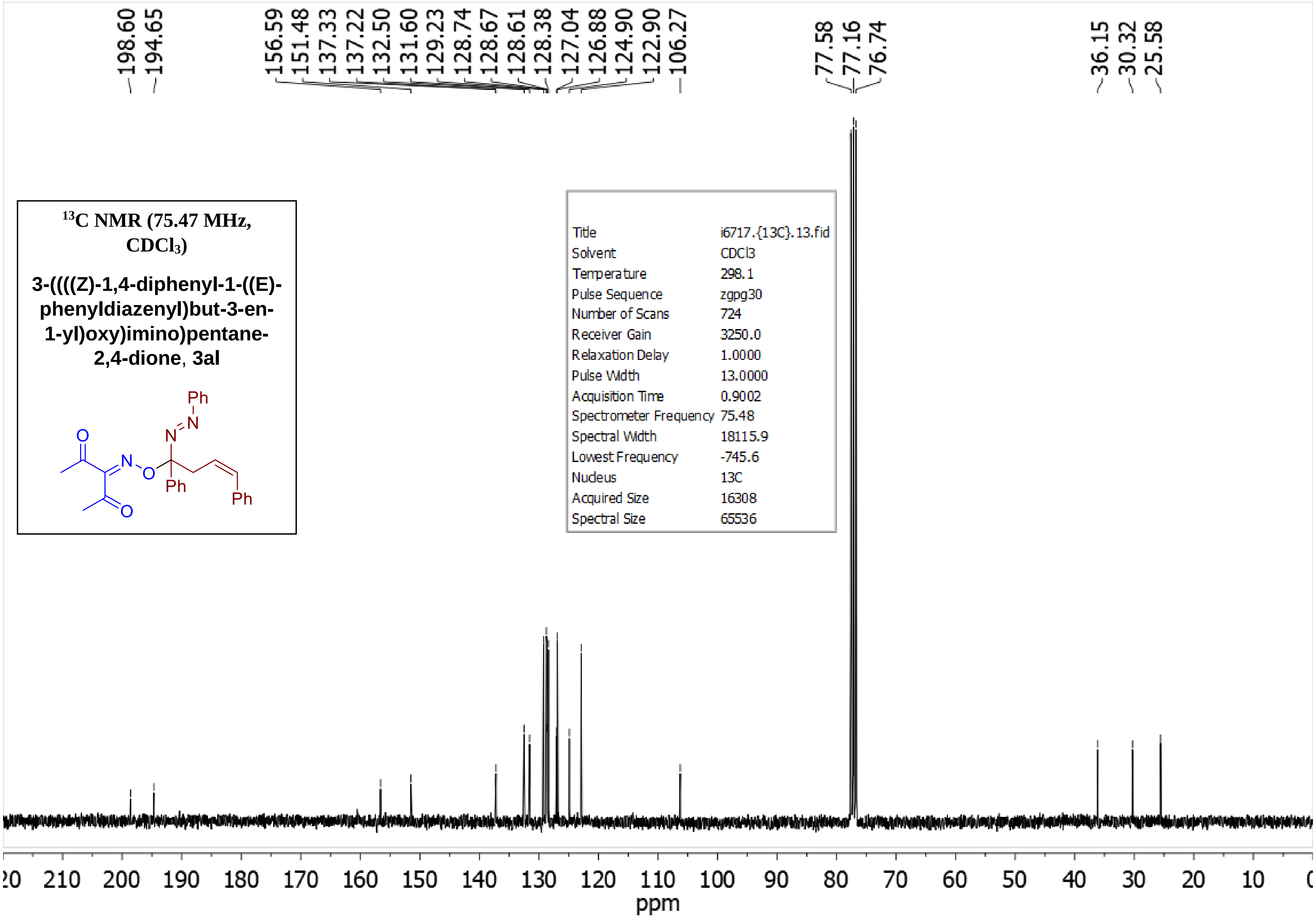
198.60
194.65
156.59
151.48
137.33
137.22
132.50
131.60
129.23
128.74
128.67
128.61
128.38
127.04
126.88
124.90
122.90
106.27
77.58
77.16
76.74
36.15
30.32
25.58
13C NMR (75.47 MHz, CDCl3)
3-((((Z)-1,4-diphenyl-1-((E)-phenyldiazenyl)but-3-en-1-yl)oxy)imino)pentane-2,4-dione, 3al
Title i6717.{13C}.13.fid
Solvent CDCl3
Temperature 298.1
Pulse Sequence zgpg30
Number of Scans 724
Receiver Gain 3250.0
Relaxation Delay 1.0000
Pulse Width 13.0000
Acquisition Time 0.9002
Spectrometer Frequency 75.48
Spectral Width 18115.9
Lowest Frequency -745.6
Nucleus 13C
Acquired Size 16308
Spectral Size 65536
ppm

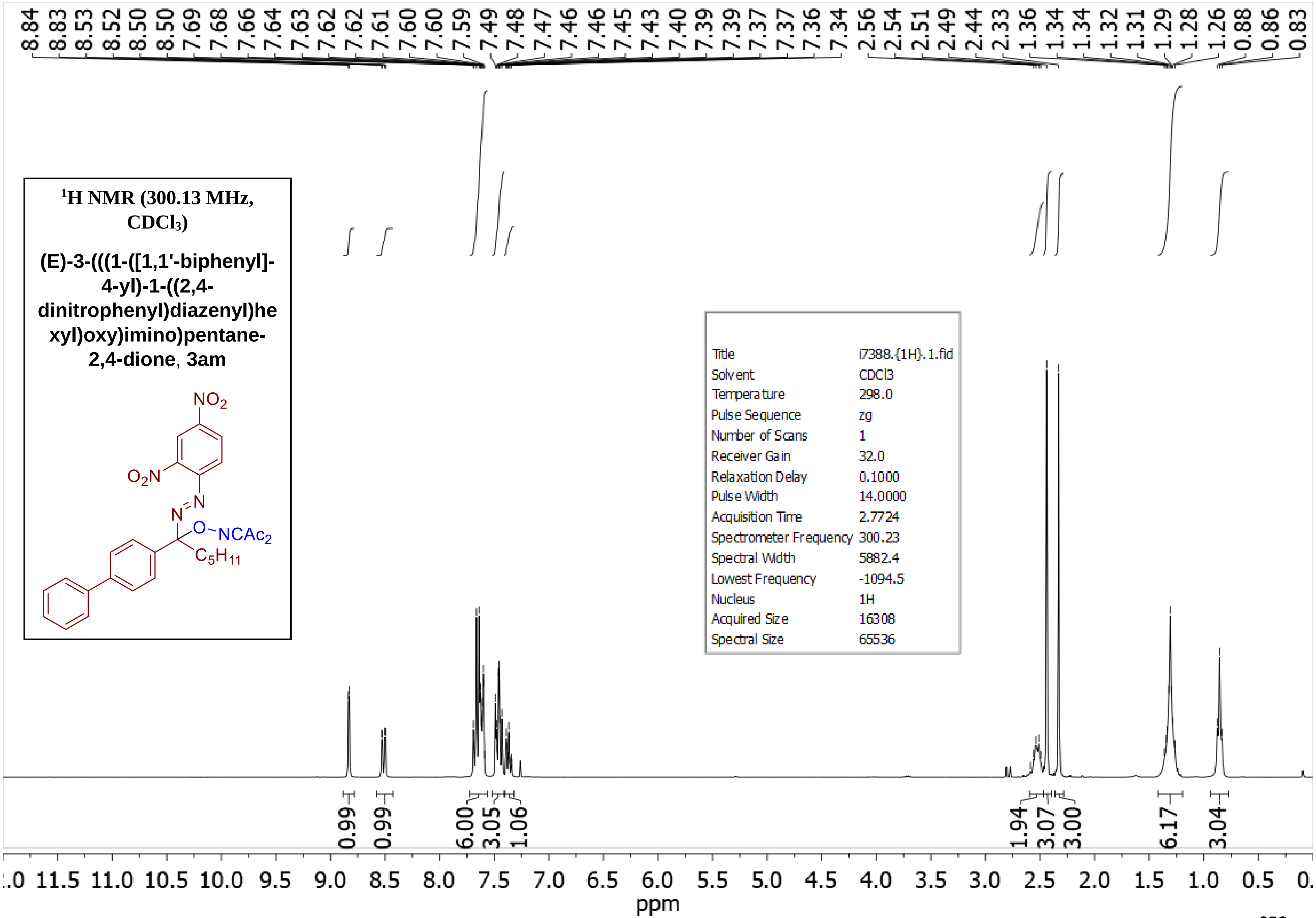
1H NMR (300.13 MHz, CDCl3)
(E)-3-(((1-([1,1'-biphenyl]-4-yl)-1-((2,4-dinitrophenyl)diazenyl)hexyl)oxy)imino)pentane-2,4-dione, 3am
NO2
O2N
N
N
O
NCAc2
C5H11
8.84 8.83 8.53 8.52 8.50 8.50 7.69 7.68 7.66 7.64 7.63 7.62 7.62 7.61 7.60 7.60 7.59 7.49 7.48 7.47 7.46 7.46 7.45 7.43 7.40 7.39 7.39 7.37 7.37 7.36 7.34 2.56 2.54 2.51 2.49 2.44 2.33 1.36 1.34 1.34 1.32 1.31 1.29 1.28 1.26 0.88 0.86 0.83
Title i7388.{1H}.1.fid
Solvent CDCl3
Temperature 298.0
Pulse Sequence zg
Number of Scans 1
Receiver Gain 32.0
Relaxation Delay 0.1000
Pulse Width 14.0000
Acquisition Time 2.7724
Spectrometer Frequency 300.23
Spectral Width 5882.4
Lowest Frequency -1094.5
Nucleus 1H
Acquired Size 16308
Spectral Size 65536
0.99 0.99 6.00 3.05 1.06 1.94 3.07 3.00 6.17 3.04
11.5 11.0 10.5 10.0 9.5 9.0 8.5 8.0 7.5 7.0 6.5 6.0 5.5 5.0 4.5 4.0 3.5 3.0 2.5 2.0 1.5 1.0 0.5
ppm

^{13}C NMR (75.47 MHz, $CDCl_3$)

(E)-3-(((1-([1,1'-biphenyl]-4-yl)-1-((2,4-dinitrophenyl)diazenyl)hexyl)oxy)imino)pentane-2,4-dione, 3am

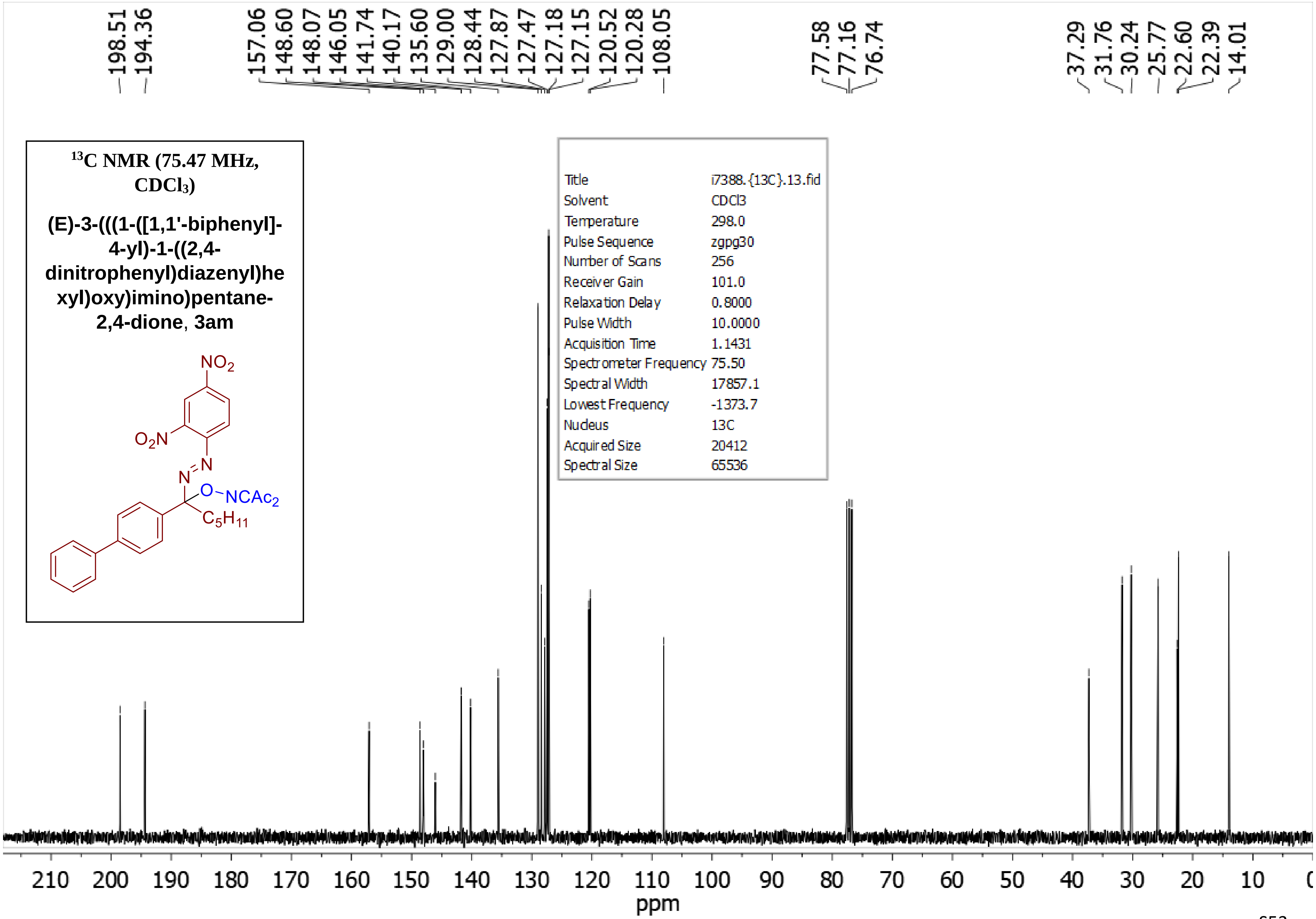

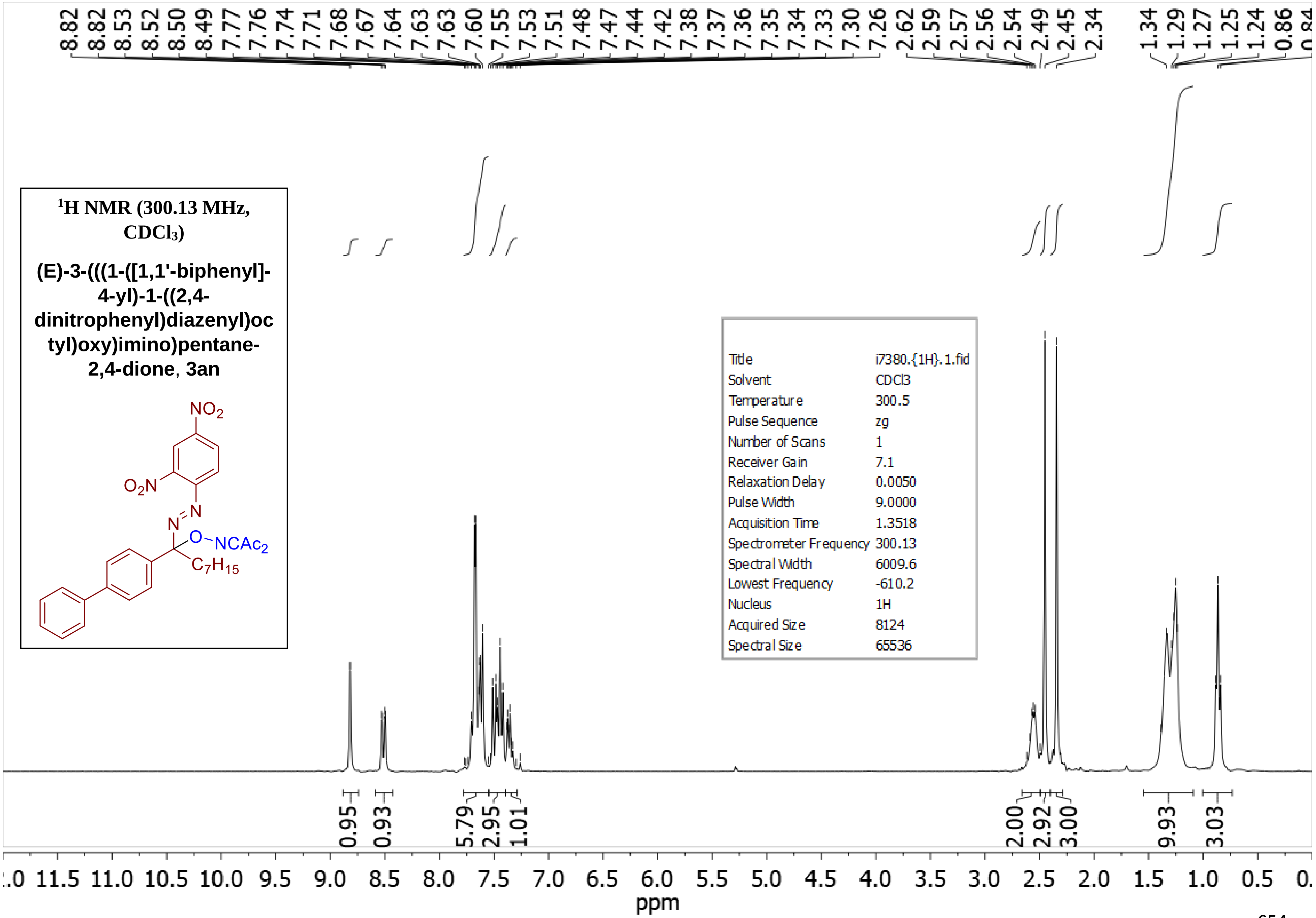

1H NMR (300.13 MHz, CDCl3)
(E)-3-(((1-([1,1'-biphenyl]-4-yl)-1-((2,4-dinitrophenyl)diazenyl)octyl)oxy)imino)pentane-2,4-dione, 3an
NO2
O2N
N=N
O
NCAc2
C7H15
Title i7380.{1H}.1.fid
Solvent CDCl3
Temperature 300.5
Pulse Sequence zg
Number of Scans 1
Receiver Gain 7.1
Relaxation Delay 0.0050
Pulse Width 9.0000
Acquisition Time 1.3518
Spectrometer Frequency 300.13
Spectral Width 6009.6
Lowest Frequency -610.2
Nucleus 1H
Acquired Size 8124
Spectral Size 65536
8.82 8.82 8.53 8.52 8.50 8.49 7.77 7.76 7.74 7.71 7.68 7.67 7.64 7.63 7.63 7.60 7.55 7.53 7.51 7.48 7.47 7.44 7.42 7.38 7.37 7.36 7.35 7.34 7.33 7.30 7.26 2.62 2.59 2.57 2.56 2.54 2.49 2.45 2.34 1.34 1.29 1.27 1.25 1.24 0.86
0.95 0.93 5.79 2.95 1.01 2.00 2.92 3.00 9.93 3.03
11.5 11.0 10.5 10.0 9.5 9.0 8.5 8.0 7.5 7.0 6.5 6.0 5.5 5.0 4.5 4.0 3.5 3.0 2.5 2.0 1.5 1.0 0.5
ppm

^{13}C NMR (75.47 MHz, $CDCl_3$)

(E)-3-(((1-([1,1'-biphenyl]-4-yl)-1-((2,4-dinitrophenyl)diazenyl)octyl)oxy)imino)pentane-2,4-dione, 3an

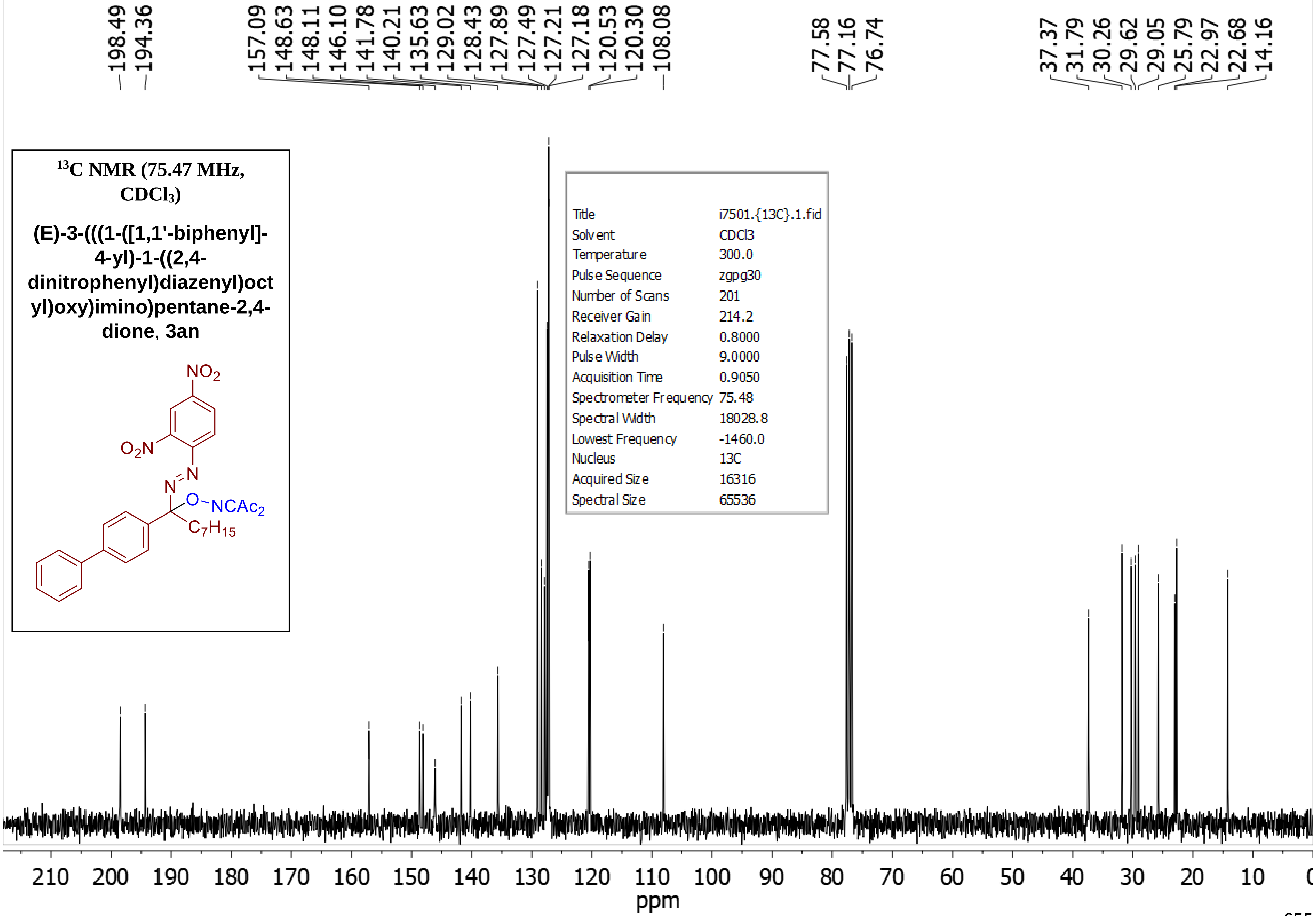

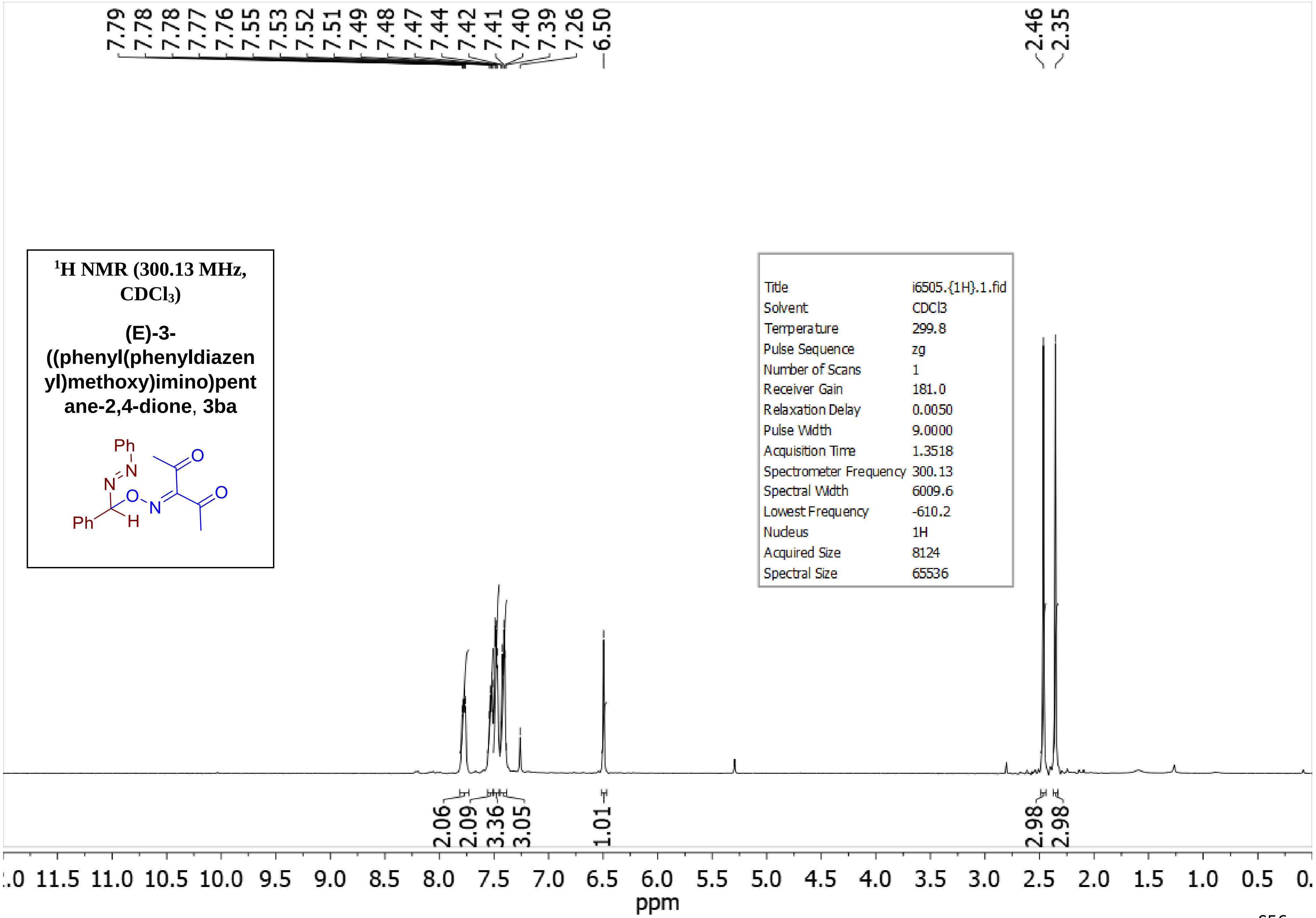

1H NMR (300.13 MHz, CDCl3)
(E)-3-((phenyl(phenyldiazenyl)methoxy)imino)pentane-2,4-dione, 3ba
Ph
N
N
O
N
H
Ph
O
O
7.79
7.78
7.78
7.77
7.76
7.55
7.53
7.52
7.51
7.49
7.48
7.47
7.44
7.42
7.41
7.40
7.39
7.26
6.50
2.46
2.35
Title i6505.{1H}.1.fid
Solvent CDCl3
Temperature 299.8
Pulse Sequence zg
Number of Scans 1
Receiver Gain 181.0
Relaxation Delay 0.0050
Pulse Width 9.0000
Acquisition Time 1.3518
Spectrometer Frequency 300.13
Spectral Width 6009.6
Lowest Frequency -610.2
Nucleus 1H
Acquired Size 8124
Spectral Size 65536
2.06
2.09
3.36
3.05
1.01
2.98
2.98
11.5 11.0 10.5 10.0 9.5 9.0 8.5 8.0 7.5 7.0 6.5 6.0 5.5 5.0 4.5 4.0 3.5 3.0 2.5 2.0 1.5 1.0 0.5
ppm

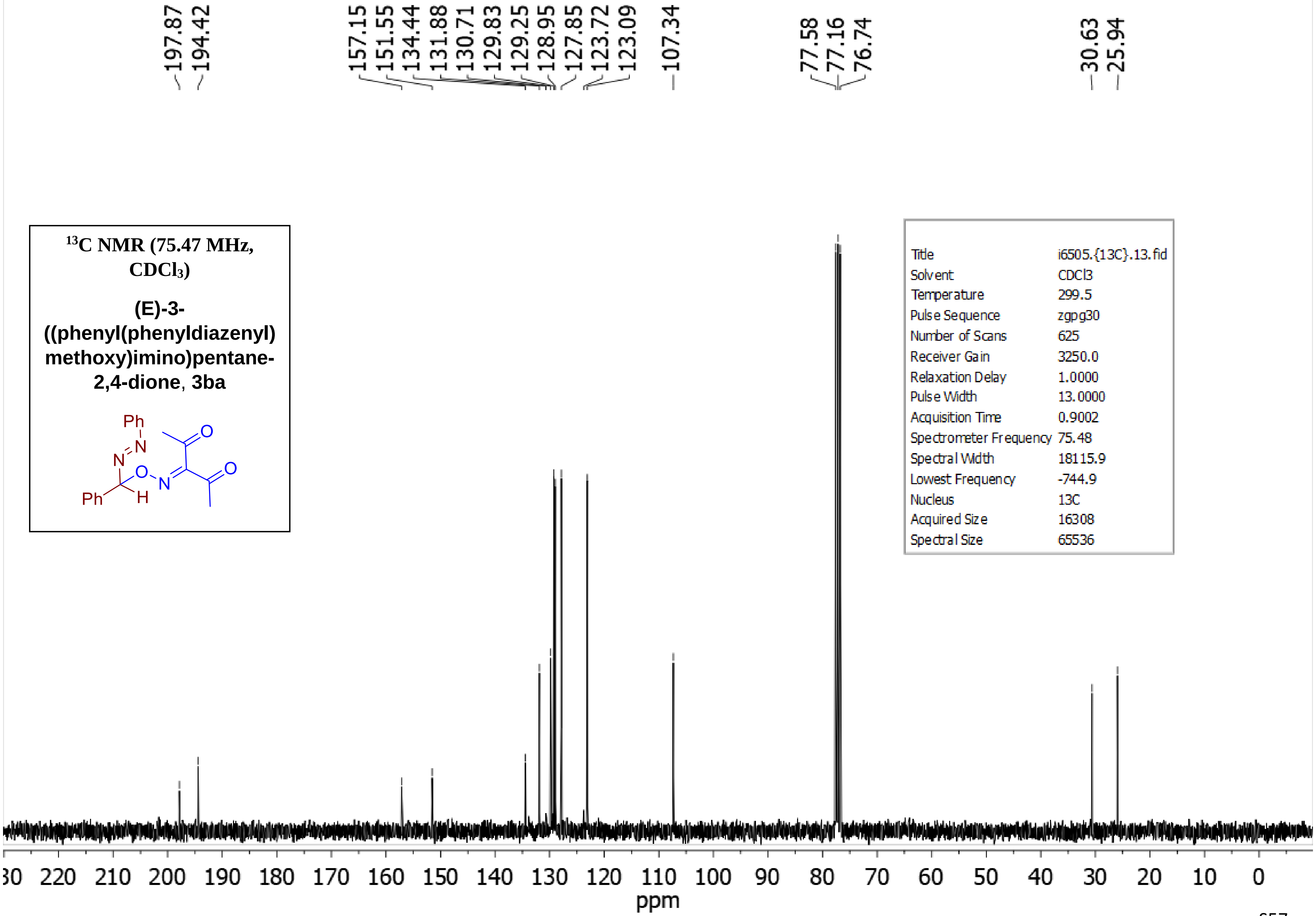

197.87
194.42
157.15
151.55
134.44
131.88
130.71
129.83
129.25
128.95
127.85
123.72
123.09
107.34
77.58
77.16
76.74
30.63
25.94
13C NMR (75.47 MHz, CDCl3)
(E)-3-((phenyl(phenyldiazenyl)methoxy)imino)pentane-2,4-dione, 3ba
Ph
N
N
O
N
O
O
Ph
H
Title i6505.{13C}.13.fid
Solvent CDCl3
Temperature 299.5
Pulse Sequence zgpg30
Number of Scans 625
Receiver Gain 3250.0
Relaxation Delay 1.0000
Pulse Width 13.0000
Acquisition Time 0.9002
Spectrometer Frequency 75.48
Spectral Width 18115.9
Lowest Frequency -744.9
Nucleus 13C
Acquired Size 16308
Spectral Size 65536
30 220 210 200 190 180 170 160 150 140 130 120 110 100 90 80 70 60 50 40 30 20 10 0
ppm

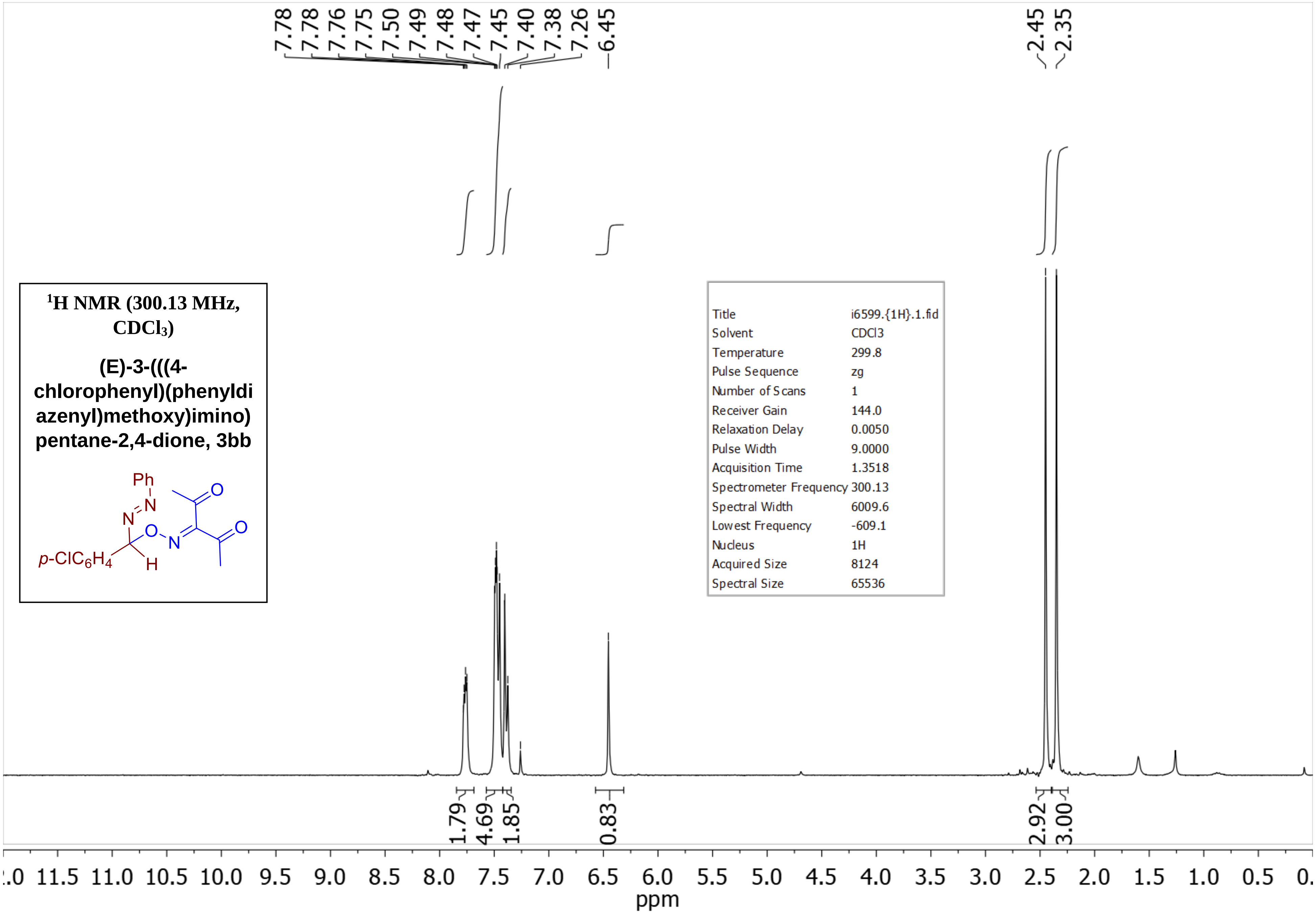

^{1}H NMR (300.13 MHz, $CDCl_3$)

(E)-3-(((4-chlorophenyl)(phenyldiazenyl)methoxy)imino)pentane-2,4-dione, 3bb

Title	i6599.{1H}.1.fid
Solvent	CDCl3
Temperature	299.8
Pulse Sequence	zg
Number of Scans	1
Receiver Gain	144.0
Relaxation Delay	0.0050
Pulse Width	9.0000
Acquisition Time	1.3518
Spectrometer Frequency	300.13
Spectral Width	6009.6
Lowest Frequency	-609.1
Nucleus	1H
Acquired Size	8124
Spectral Size	65536

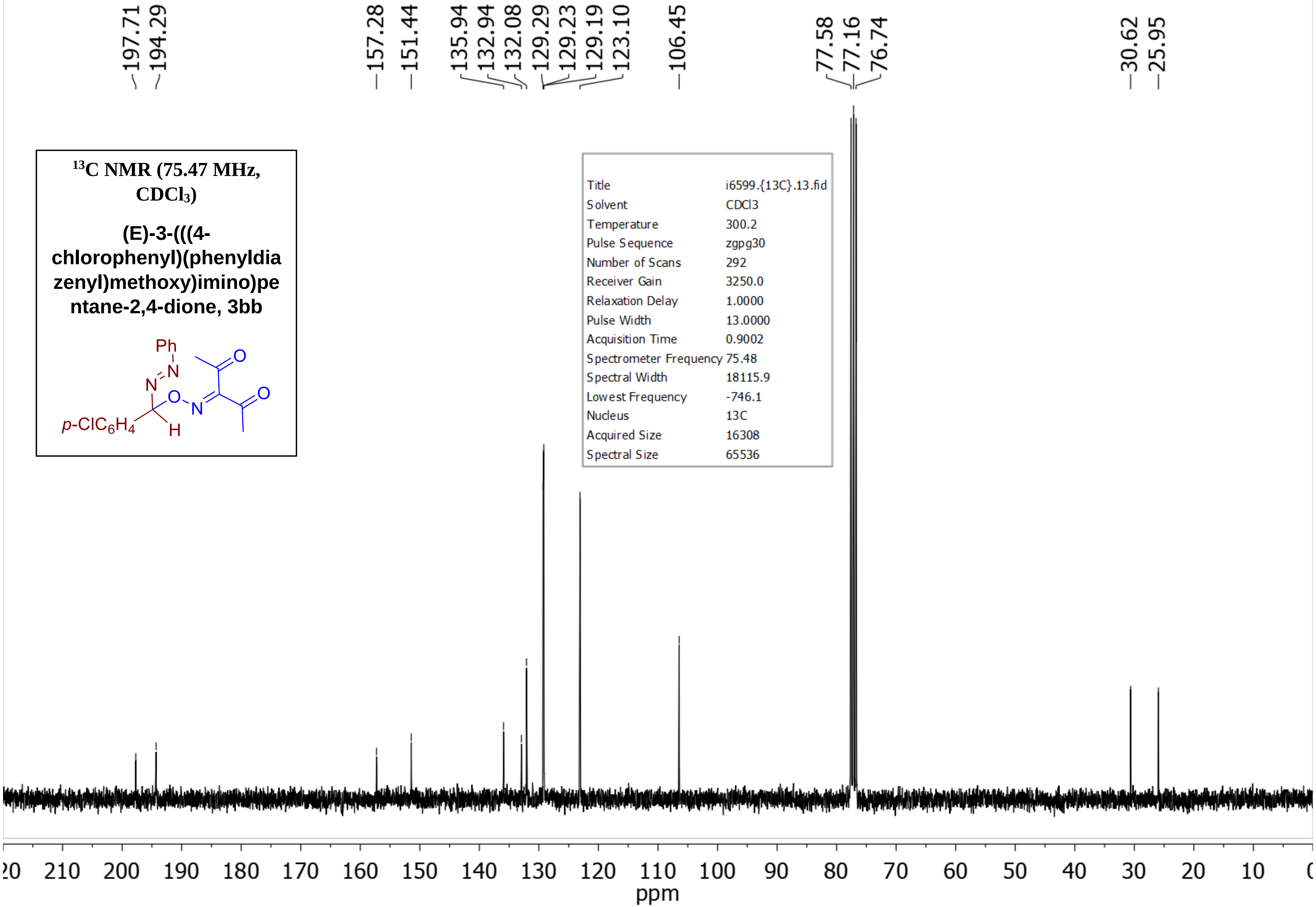

197.71
194.29
157.28
151.44
135.94
132.94
132.08
129.29
129.23
129.19
123.10
106.45
77.58
77.16
76.74
30.62
25.95
^{13}C NMR (75.47 MHz, $CDCl_3$)
(E)-3-(((4-chlorophenyl)(phenyldiazenyl)methoxy)imino)pentane-2,4-dione, 3bb
Title i6599.{13C}.13.fid
Solvent CDCl3
Temperature 300.2
Pulse Sequence zgpg30
Number of Scans 292
Receiver Gain 3250.0
Relaxation Delay 1.0000
Pulse Width 13.0000
Acquisition Time 0.9002
Spectrometer Frequency 75.48
Spectral Width 18115.9
Lowest Frequency -746.1
Nucleus 13C
Acquired Size 16308
Spectral Size 65536
210 200 190 180 170 160 150 140 130 120 110 100 90 80 70 60 50 40 30 20 10
ppm

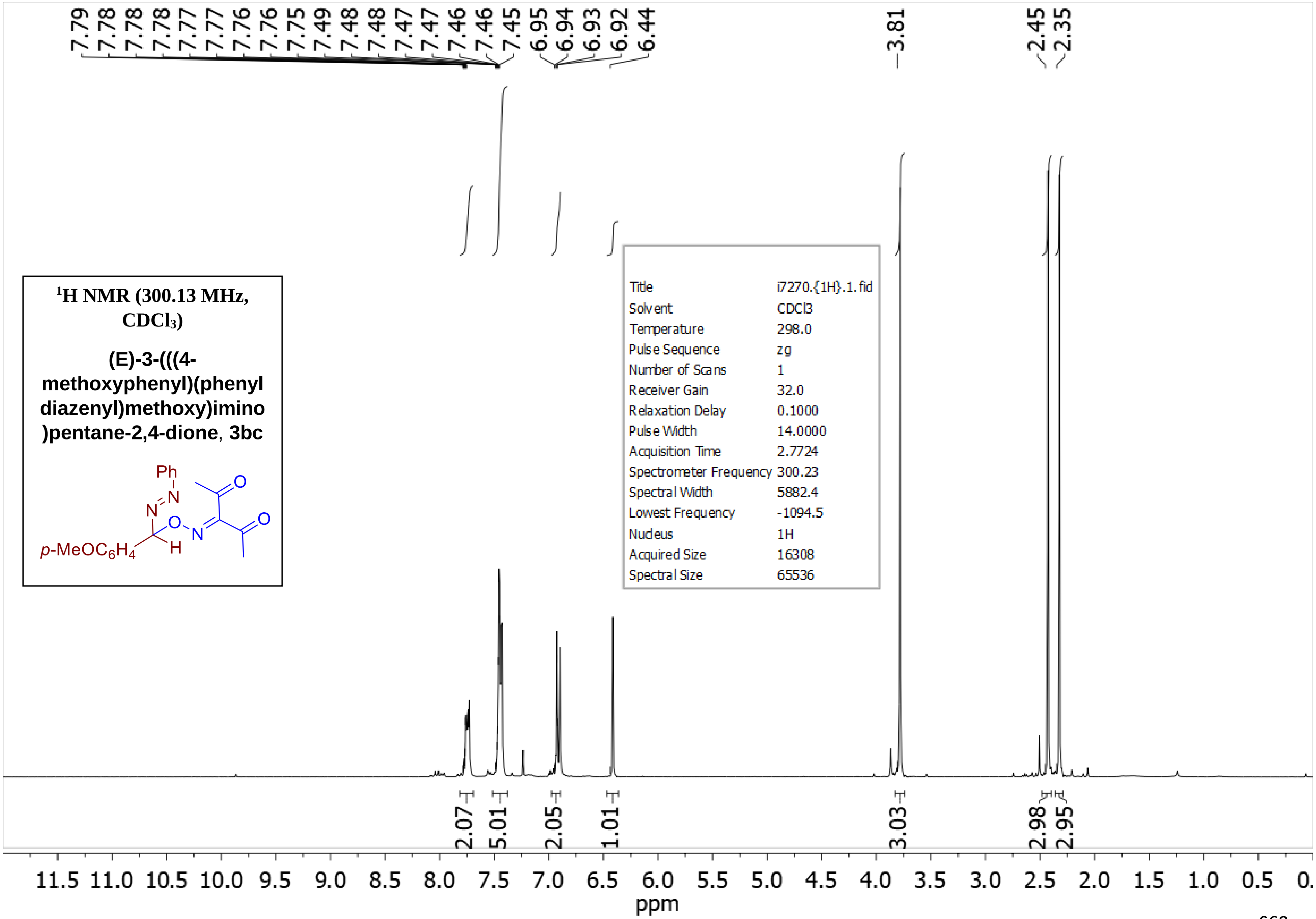

7.79 7.78 7.78 7.78 7.77 7.77 7.76 7.76 7.75 7.49 7.48 7.48 7.47 7.47 7.46 7.46 7.45 6.95 6.94 6.93 6.92 6.44
3.81
2.45 2.35
^{1}H NMR (300.13 MHz, $CDCl_3$)
(E)-3-(((4-methoxyphenyl)(phenyldiazenyl)methoxy)imino)pentane-2,4-dione, 3bc
Ph
N
N
O
N
O
O
H
p-MeOC$_6$H$_4$
Title i7270.{1H}.1.fid
Solvent CDCl3
Temperature 298.0
Pulse Sequence zg
Number of Scans 1
Receiver Gain 32.0
Relaxation Delay 0.1000
Pulse Width 14.0000
Acquisition Time 2.7724
Spectrometer Frequency 300.23
Spectral Width 5882.4
Lowest Frequency -1094.5
Nucleus 1H
Acquired Size 16308
Spectral Size 65536
2.07 5.01 2.05 1.01 3.03 2.98 2.95
11.5 11.0 10.5 10.0 9.5 9.0 8.5 8.0 7.5 7.0 6.5 6.0 5.5 5.0 4.5 4.0 3.5 3.0 2.5 2.0 1.5 1.0 0.5 0.
ppm

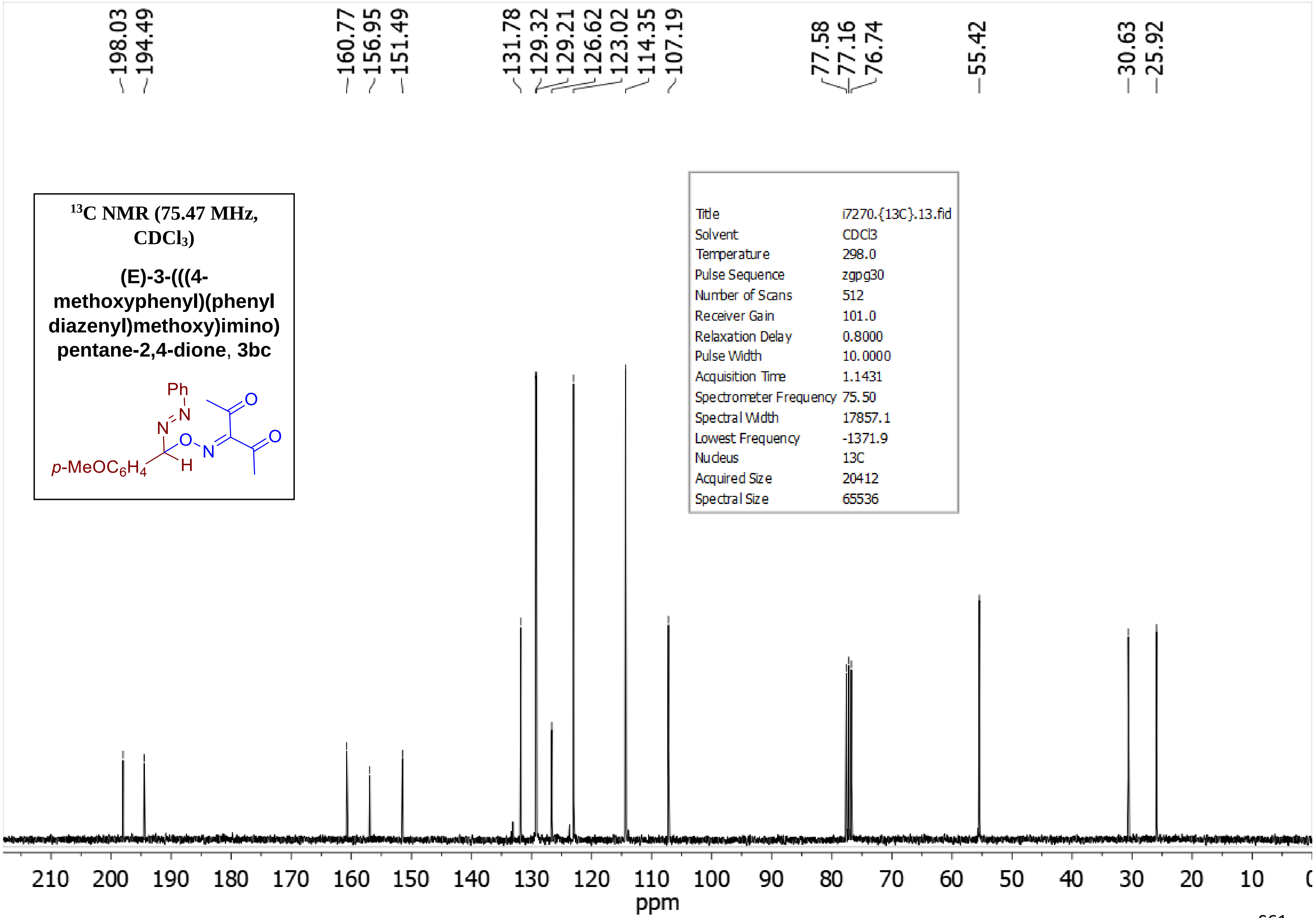

198.03
194.49
160.77
156.95
151.49
131.78
129.32
129.21
126.62
123.02
114.35
107.19
77.58
77.16
76.74
55.42
30.63
25.92
^{13}C NMR (75.47 MHz, $CDCl_3$)
(E)-3-(((4-methoxyphenyl)(phenyldiazenyl)methoxy)imino)pentane-2,4-dione, 3bc
Ph
N
N
O
N
O
O
H
p-MeOC$_6$H$_4$
Title i7270.{13C}.13.fid
Solvent CDCl3
Temperature 298.0
Pulse Sequence zgpg30
Number of Scans 512
Receiver Gain 101.0
Relaxation Delay 0.8000
Pulse Width 10.0000
Acquisition Time 1.1431
Spectrometer Frequency 75.50
Spectral Width 17857.1
Lowest Frequency -1371.9
Nucleus 13C
Acquired Size 20412
Spectral Size 65536
210 200 190 180 170 160 150 140 130 120 110 100 90 80 70 60 50 40 30 20 10 0
ppm

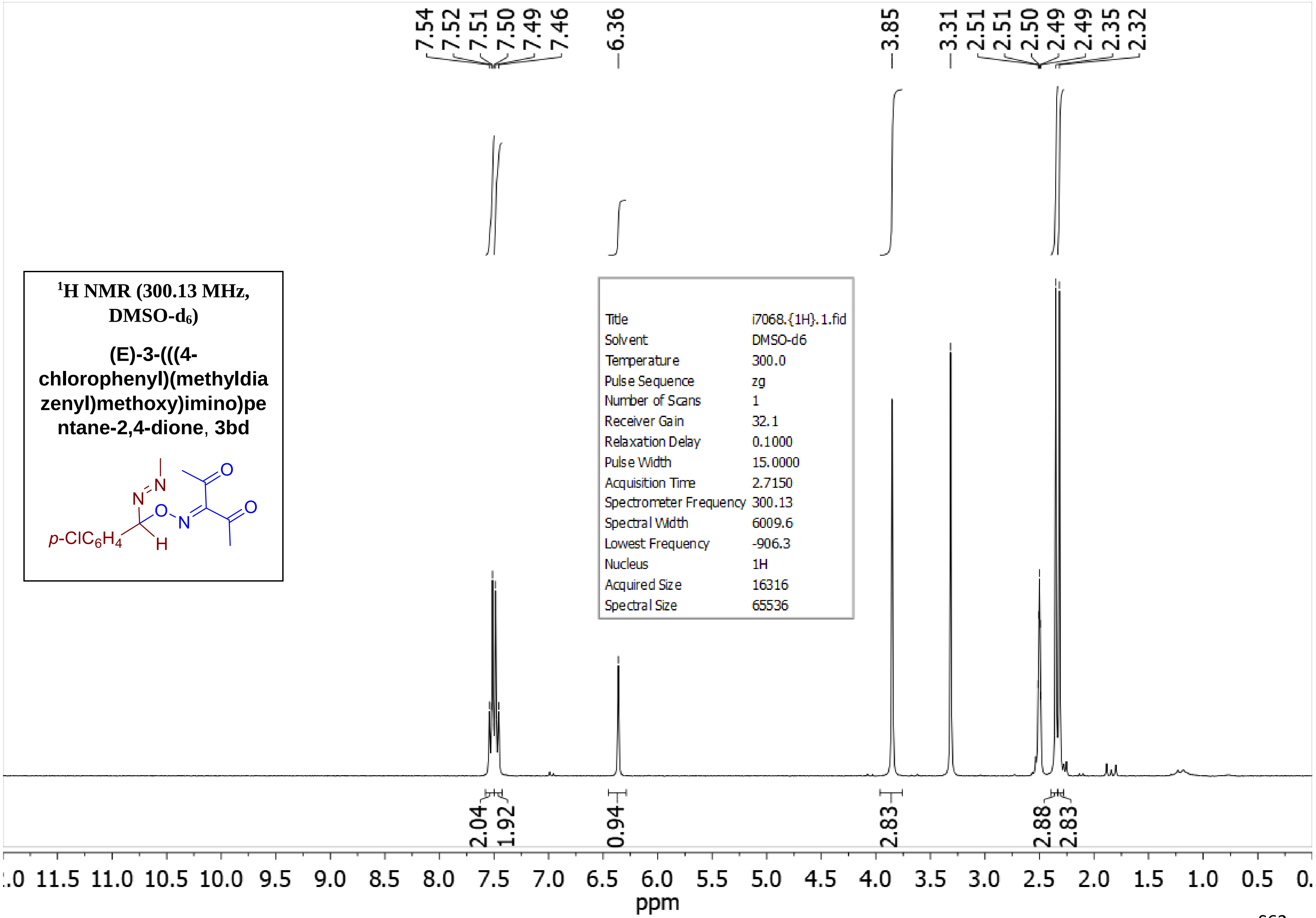

1H NMR (300.13 MHz, DMSO-d6)
(E)-3-(((4-chlorophenyl)(methyldiazenyl)methoxy)imino)pentane-2,4-dione, 3bd
p-ClC6H4
7.54 7.52 7.51 7.50 7.49 7.46
6.36
3.85
3.31 2.51 2.51 2.50 2.49 2.49 2.35 2.32
Title i7068.{1H}.1.fid
Solvent DMSO-d6
Temperature 300.0
Pulse Sequence zg
Number of Scans 1
Receiver Gain 32.1
Relaxation Delay 0.1000
Pulse Width 15.0000
Acquisition Time 2.7150
Spectrometer Frequency 300.13
Spectral Width 6009.6
Lowest Frequency -906.3
Nucleus 1H
Acquired Size 16316
Spectral Size 65536
2.04 1.92
0.94
2.83
2.88 2.83
11.5 11.0 10.5 10.0 9.5 9.0 8.5 8.0 7.5 7.0 6.5 6.0 5.5 5.0 4.5 4.0 3.5 3.0 2.5 2.0 1.5 1.0 0.5
ppm

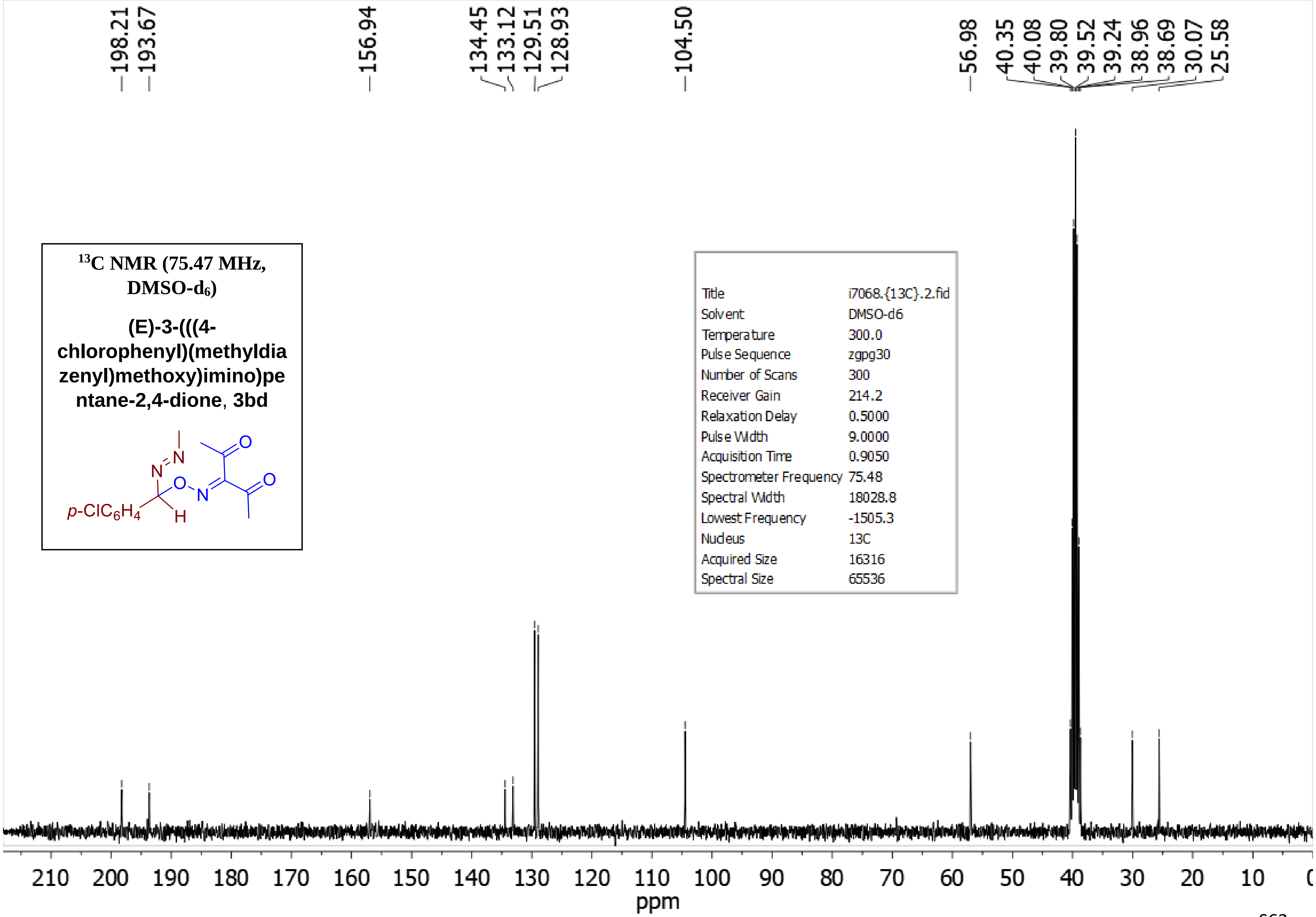
198.21
193.67
156.94
134.45
133.12
129.51
128.93
104.50
56.98
40.35
40.08
39.80
39.52
39.24
38.96
38.69
30.07
25.58
13C NMR (75.47 MHz, DMSO-d6)
(E)-3-(((4-chlorophenyl)(methyldiazenyl)methoxy)imino)pentane-2,4-dione, 3bd
N
N
O
N
O
O
p-ClC6H4
H
Title i7068.{13C}.2.fid
Solvent DMSO-d6
Temperature 300.0
Pulse Sequence zgpg30
Number of Scans 300
Receiver Gain 214.2
Relaxation Delay 0.5000
Pulse Width 9.0000
Acquisition Time 0.9050
Spectrometer Frequency 75.48
Spectral Width 18028.8
Lowest Frequency -1505.3
Nucleus 13C
Acquired Size 16316
Spectral Size 65536
210
200
190
180
170
160
150
140
130
120
110
100
90
80
70
60
50
40
30
20
10
ppm

^{1}H NMR (300.13 MHz, $CDCl_3$)

(E)-3-(((2-(phenyldiazenyl)propan-2-yl)oxy)imino)pentane-2,4-dione, 3ca

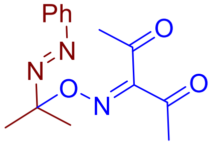

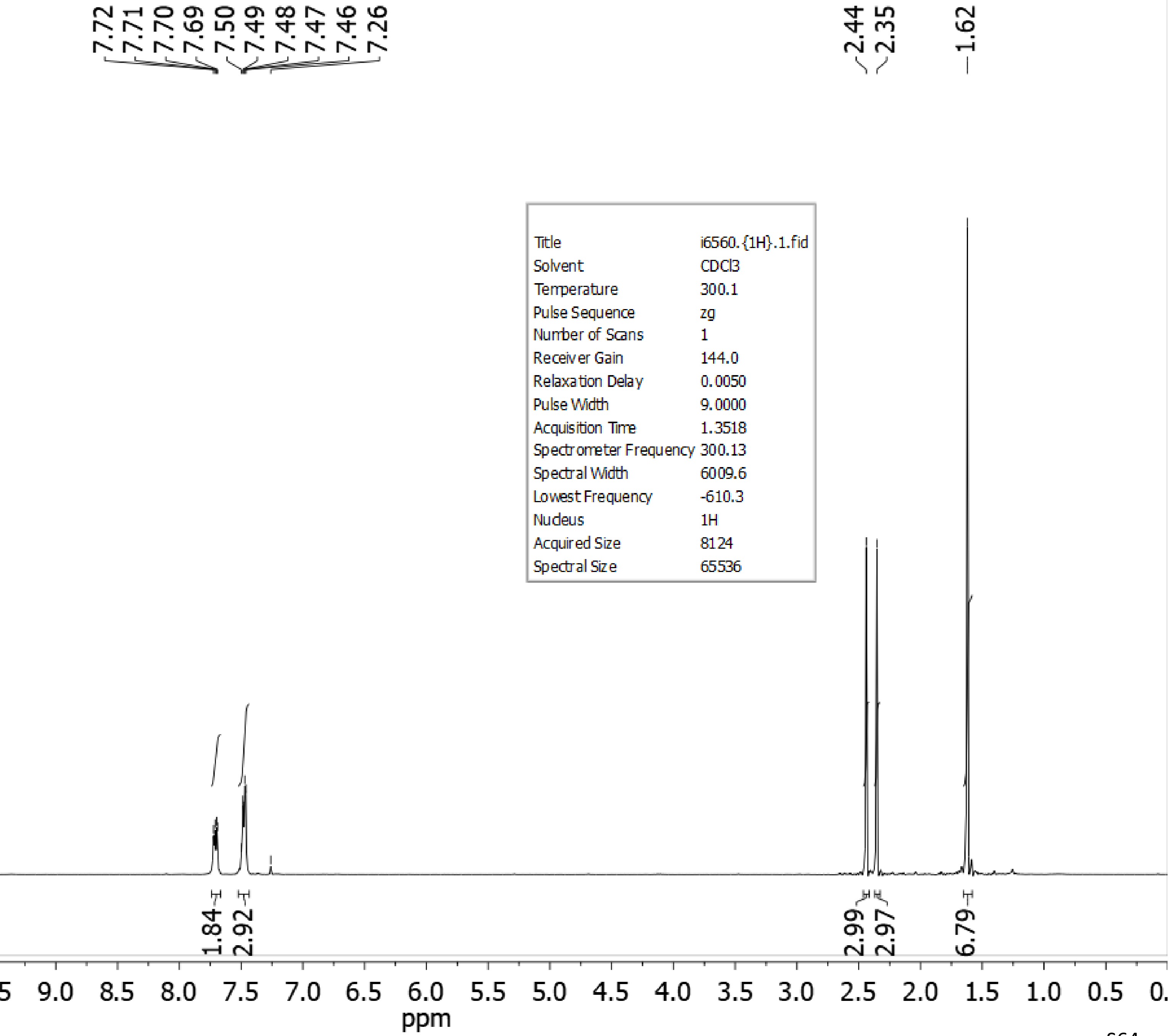

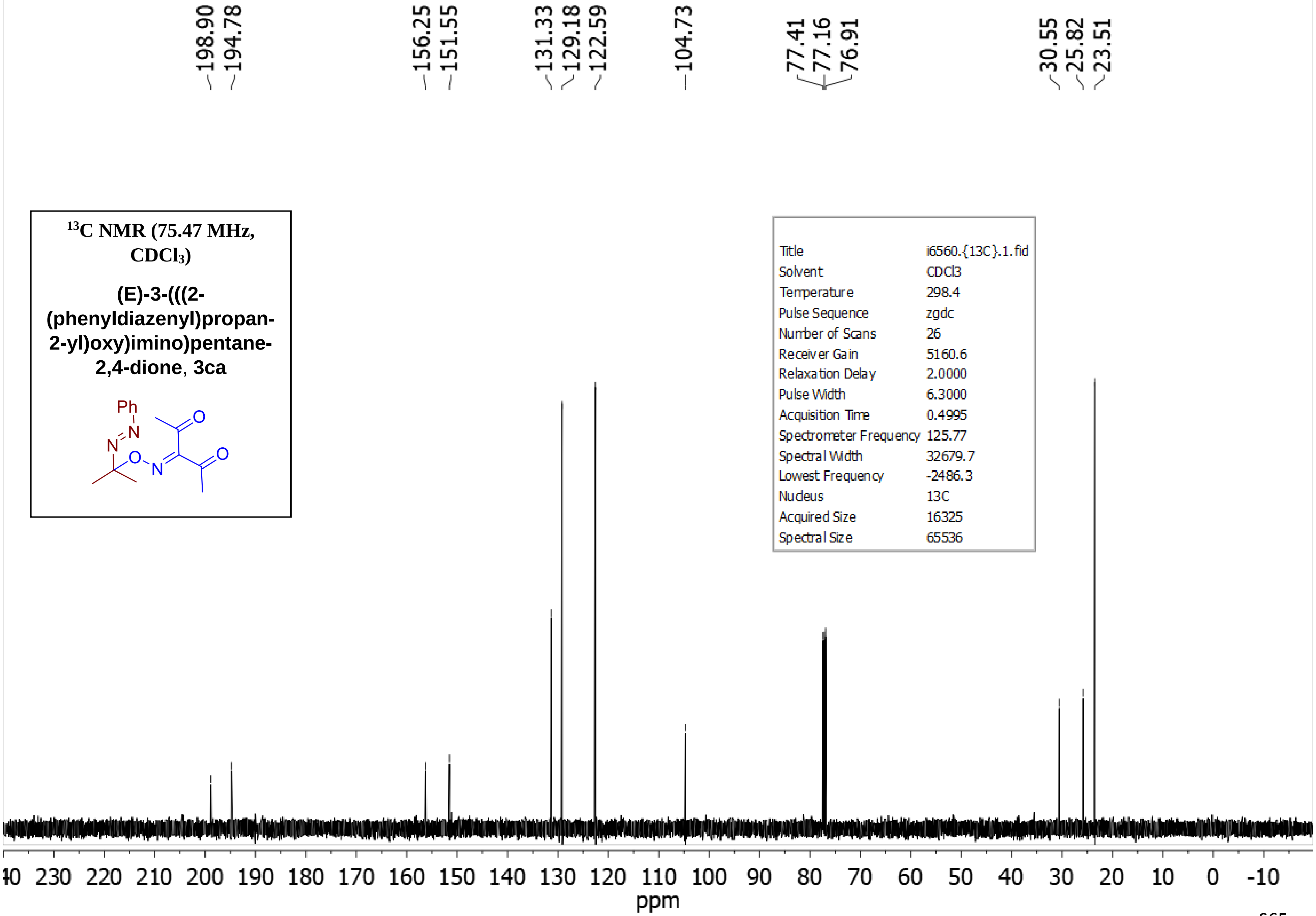

^{13}C NMR (75.47 MHz, $CDCl_3$)

(E)-3-(((2-(phenyldiazenyl)propan-2-yl)oxy)imino)pentane-2,4-dione, 3ca

Title	i6560.{13C}.1.fid
Solvent	CDCl3
Temperature	298.4
Pulse Sequence	zgdc
Number of Scans	26
Receiver Gain	5160.6
Relaxation Delay	2.0000
Pulse Width	6.3000
Acquisition Time	0.4995
Spectrometer Frequency	125.77
Spectral Width	32679.7
Lowest Frequency	-2486.3
Nucleus	13C
Acquired Size	16325
Spectral Size	65536

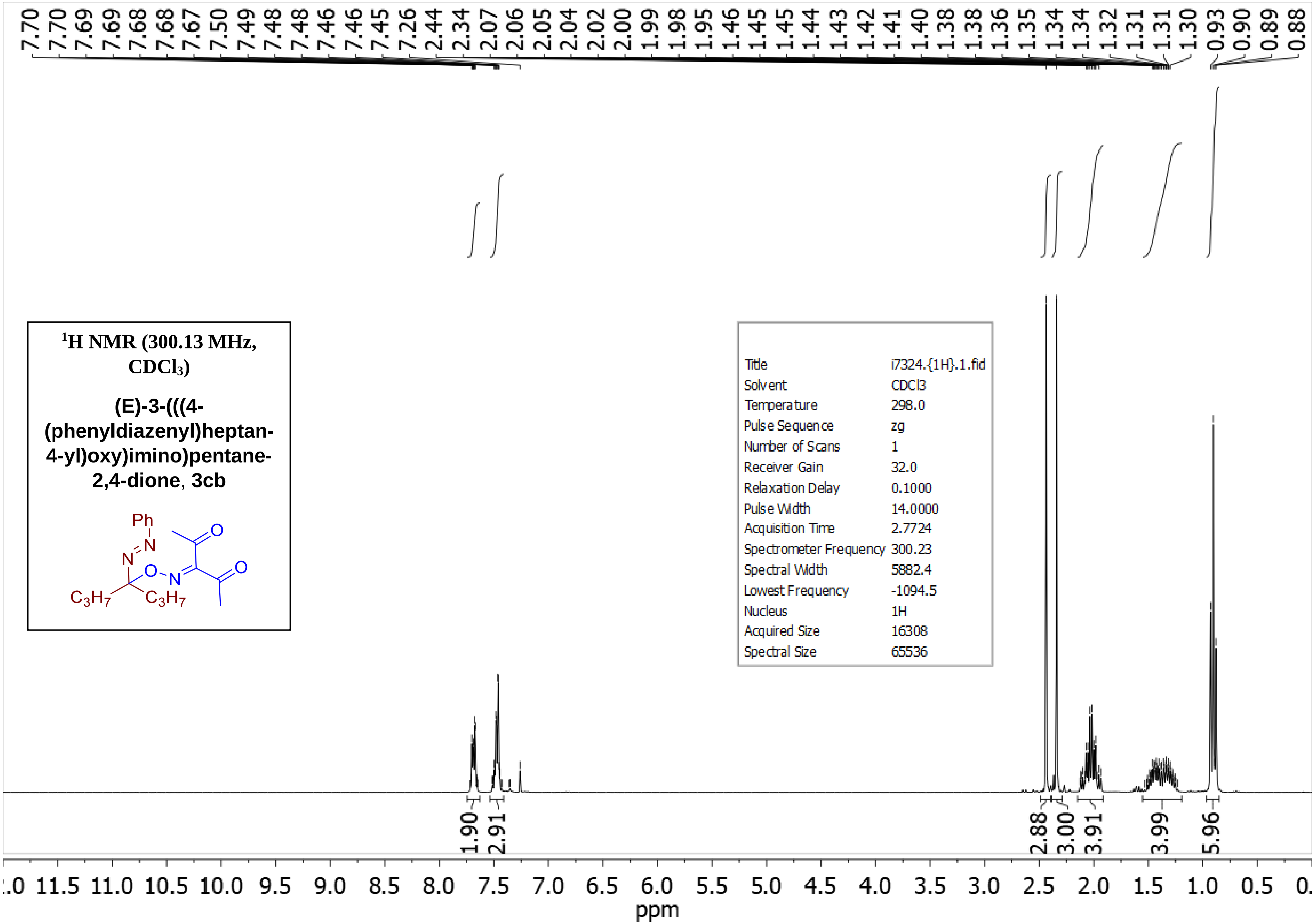
7.70 7.70 7.69 7.69 7.68 7.68 7.67 7.50 7.49 7.48 7.48 7.46 7.46 7.45 7.26 2.44 2.34 2.07 2.06 2.05 2.04 2.02 2.00 1.99 1.98 1.95 1.46 1.45 1.45 1.44 1.43 1.42 1.41 1.40 1.38 1.38 1.36 1.35 1.34 1.34 1.32 1.31 1.31 1.30 0.93 0.90 0.89 0.88
1H NMR (300.13 MHz, CDCl3)
(E)-3-(((4-(phenyldiazenyl)heptan-4-yl)oxy)imino)pentane-2,4-dione, 3cb
Ph
N
N
O
N
O
O
C3H7
C3H7
Title i7324.{1H}.1.fid
Solvent CDCl3
Temperature 298.0
Pulse Sequence zg
Number of Scans 1
Receiver Gain 32.0
Relaxation Delay 0.1000
Pulse Width 14.0000
Acquisition Time 2.7724
Spectrometer Frequency 300.23
Spectral Width 5882.4
Lowest Frequency -1094.5
Nucleus 1H
Acquired Size 16308
Spectral Size 65536
1.90
2.91
2.88
3.00
3.91
3.99
5.96
11.5 11.0 10.5 10.0 9.5 9.0 8.5 8.0 7.5 7.0 6.5 6.0 5.5 5.0 4.5 4.0 3.5 3.0 2.5 2.0 1.5 1.0 0.5
ppm

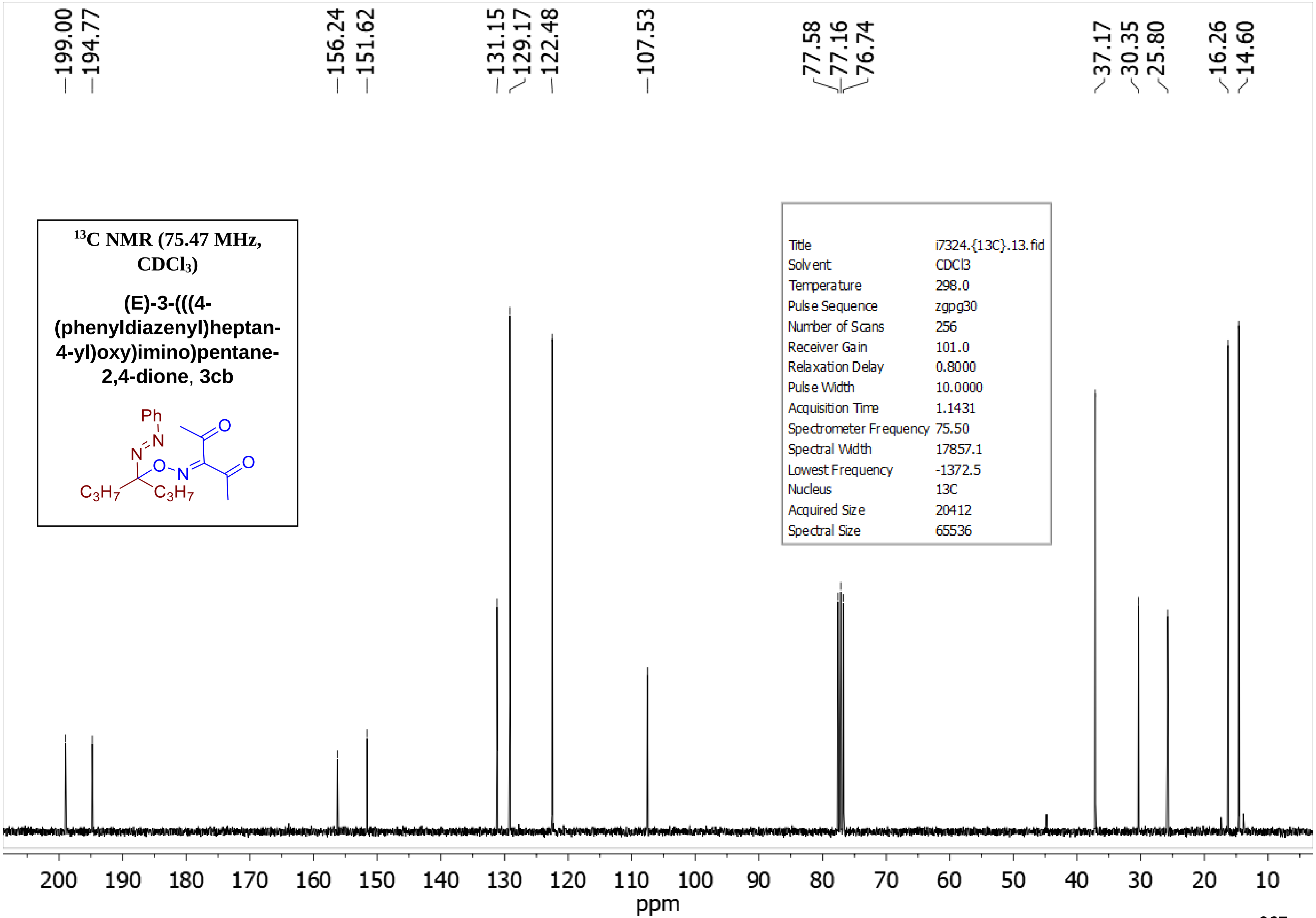
199.00
194.77
156.24
151.62
131.15
129.17
122.48
107.53
77.58
77.16
76.74
37.17
30.35
25.80
16.26
14.60
13C NMR (75.47 MHz, CDCl3)
(E)-3-(((4-(phenyldiazenyl)heptan-4-yl)oxy)imino)pentane-2,4-dione, 3cb
Ph
N
N
O
N
O
O
C3H7
C3H7
Title i7324.{13C}.13.fid
Solvent CDCl3
Temperature 298.0
Pulse Sequence zgpg30
Number of Scans 256
Receiver Gain 101.0
Relaxation Delay 0.8000
Pulse Width 10.0000
Acquisition Time 1.1431
Spectrometer Frequency 75.50
Spectral Width 17857.1
Lowest Frequency -1372.5
Nucleus 13C
Acquired Size 20412
Spectral Size 65536
200
190
180
170
160
150
140
130
120
110
100
90
80
70
60
50
40
30
20
10
ppm

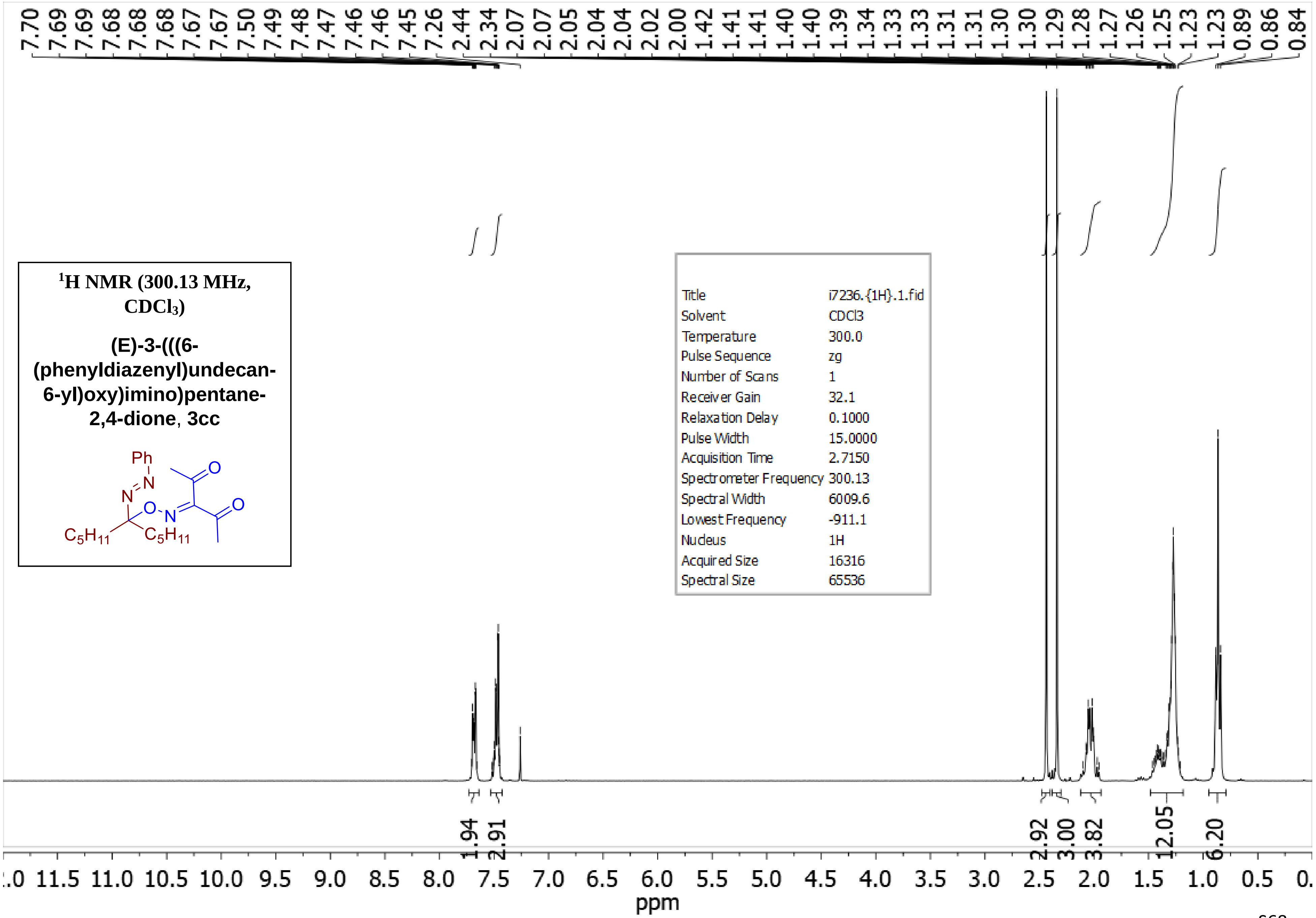

7.70 7.69 7.69 7.68 7.68 7.68 7.67 7.67 7.50 7.49 7.48 7.47 7.46 7.46 7.45 7.26 2.44 2.34 2.07 2.07 2.05 2.04 2.04 2.02 2.00 1.42 1.41 1.41 1.40 1.40 1.39 1.34 1.33 1.33 1.31 1.31 1.30 1.30 1.29 1.28 1.27 1.26 1.25 1.23 1.23 0.89 0.86 0.84
1H NMR (300.13 MHz, CDCl3)
(E)-3-(((6-(phenyldiazenyl)undecan-6-yl)oxy)imino)pentane-2,4-dione, 3cc
Ph
N
N
O
N
O
O
C5H11
C5H11
Title i7236.{1H}.1.fid
Solvent CDCl3
Temperature 300.0
Pulse Sequence zg
Number of Scans 1
Receiver Gain 32.1
Relaxation Delay 0.1000
Pulse Width 15.0000
Acquisition Time 2.7150
Spectrometer Frequency 300.13
Spectral Width 6009.6
Lowest Frequency -911.1
Nucleus 1H
Acquired Size 16316
Spectral Size 65536
1.94 2.91 2.92 3.00 3.82 12.05 6.20
.0 11.5 11.0 10.5 10.0 9.5 9.0 8.5 8.0 7.5 7.0 6.5 6.0 5.5 5.0 4.5 4.0 3.5 3.0 2.5 2.0 1.5 1.0 0.5 0.
ppm

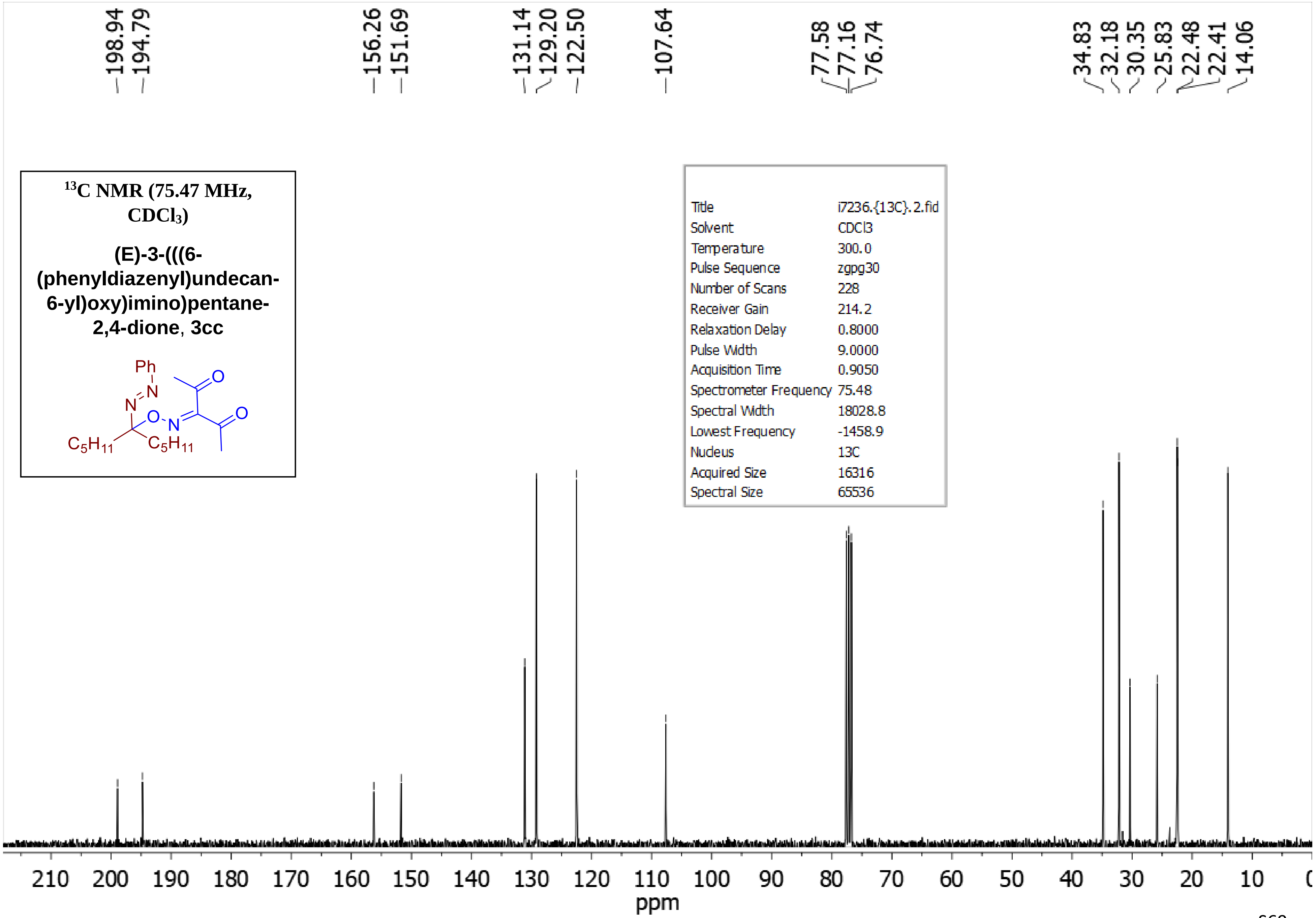

198.94
194.79
156.26
151.69
131.14
129.20
122.50
107.64
77.58
77.16
76.74
34.83
32.18
30.35
25.83
22.48
22.41
14.06
^{13}C NMR (75.47 MHz, $CDCl_3$)
(E)-3-(((6-(phenyldiazenyl)undecan-6-yl)oxy)imino)pentane-2,4-dione, 3cc
Ph
N
N
O
N
O
O
C_5H_{11}
C_5H_{11}
Title i7236.{13C}.2.fid
Solvent CDCl3
Temperature 300.0
Pulse Sequence zgpg30
Number of Scans 228
Receiver Gain 214.2
Relaxation Delay 0.8000
Pulse Width 9.0000
Acquisition Time 0.9050
Spectrometer Frequency 75.48
Spectral Width 18028.8
Lowest Frequency -1458.9
Nucleus 13C
Acquired Size 16316
Spectral Size 65536
210 200 190 180 170 160 150 140 130 120 110 100 90 80 70 60 50 40 30 20 10
ppm

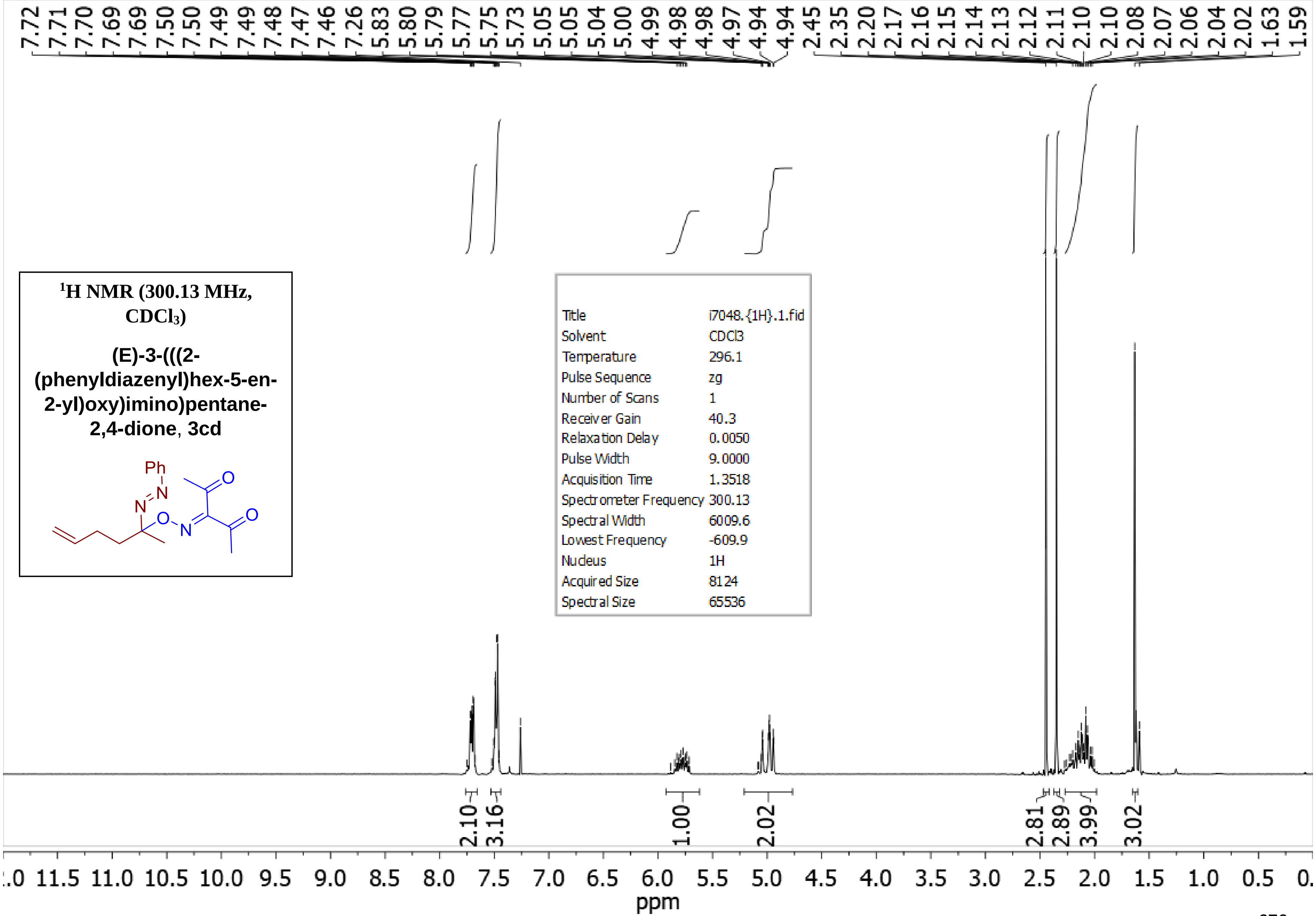

^{1}H NMR (300.13 MHz, $CDCl_3$)

(E)-3-(((2-(phenyldiazenyl)hex-5-en-2-yl)oxy)imino)pentane-2,4-dione, 3cd

Title	i7048.{1H}.1.fid
Solvent	CDCl3
Temperature	296.1
Pulse Sequence	zg
Number of Scans	1
Receiver Gain	40.3
Relaxation Delay	0.0050
Pulse Width	9.0000
Acquisition Time	1.3518
Spectrometer Frequency	300.13
Spectral Width	6009.6
Lowest Frequency	-609.9
Nucleus	1H
Acquired Size	8124
Spectral Size	65536

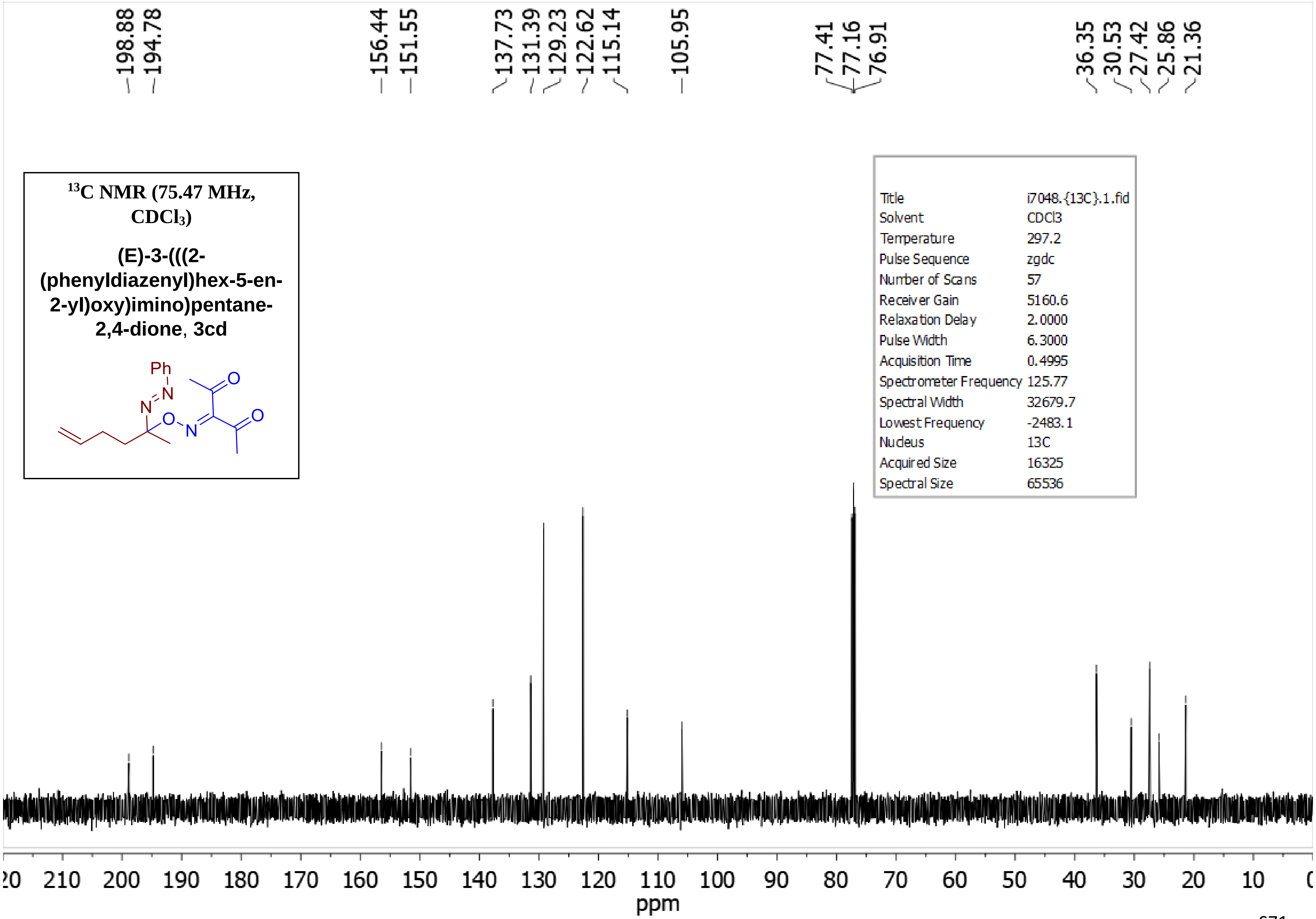
198.88
194.78
156.44
151.55
137.73
131.39
129.23
122.62
115.14
105.95
77.41
77.16
76.91
36.35
30.53
27.42
25.86
21.36
¹³C NMR (75.47 MHz, CDCl₃)
(E)-3-(((2-(phenyldiazenyl)hex-5-en-2-yl)oxy)imino)pentane-2,4-dione, 3cd
Ph
N
N
O
N
O
O
Title i7048.{13C}.1.fid
Solvent CDCl3
Temperature 297.2
Pulse Sequence zgdc
Number of Scans 57
Receiver Gain 5160.6
Relaxation Delay 2.0000
Pulse Width 6.3000
Acquisition Time 0.4995
Spectrometer Frequency 125.77
Spectral Width 32679.7
Lowest Frequency -2483.1
Nucleus 13C
Acquired Size 16325
Spectral Size 65536
210 200 190 180 170 160 150 140 130 120 110 100 90 80 70 60 50 40 30 20 10
ppm

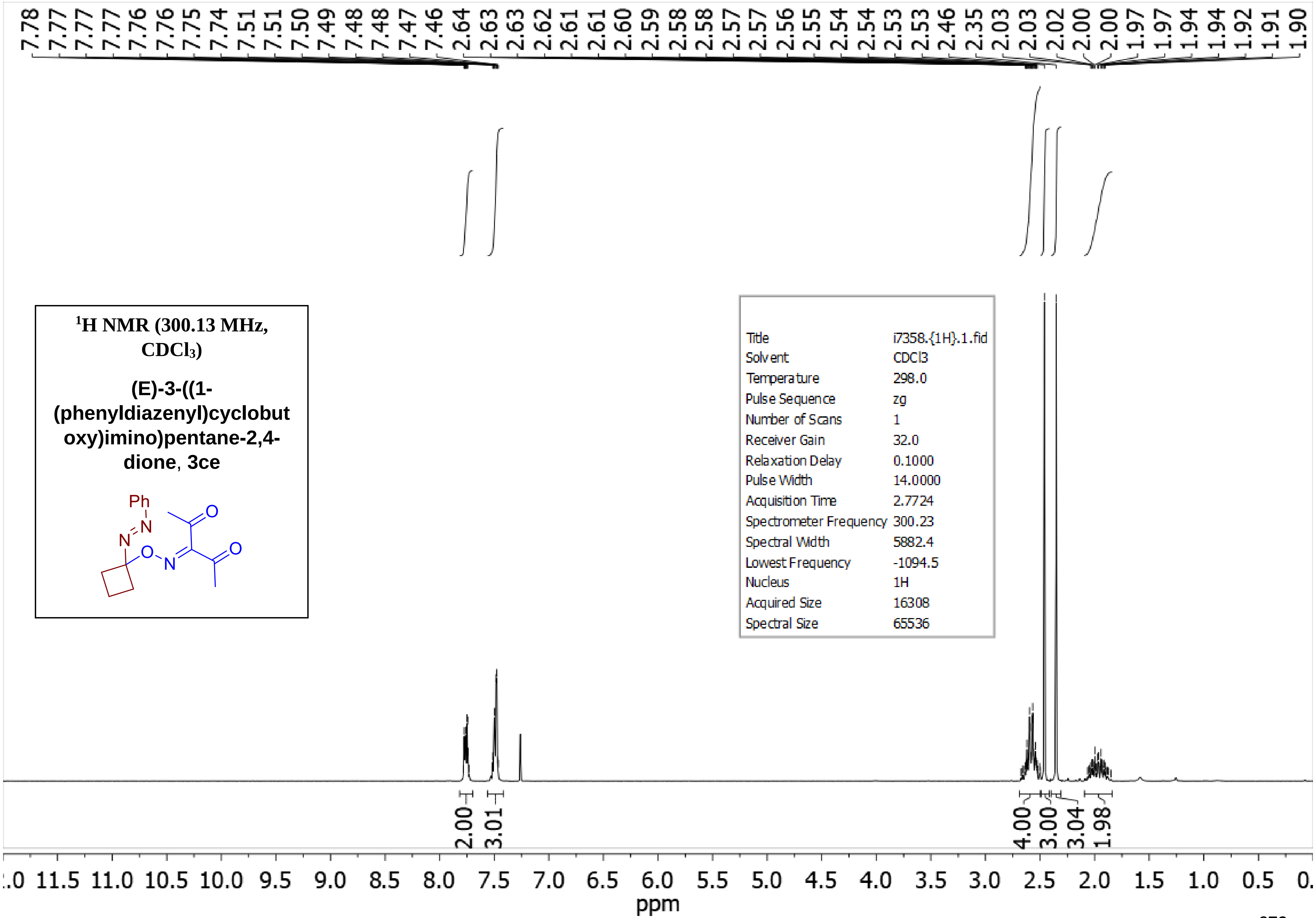
7.78 7.77 7.77 7.77 7.76 7.76 7.75 7.74 7.51 7.51 7.50 7.49 7.48 7.48 7.47 7.46 2.64 2.63 2.63 2.62 2.61 2.61 2.60 2.59 2.58 2.58 2.57 2.57 2.56 2.55 2.54 2.54 2.53 2.53 2.53 2.46 2.35 2.03 2.03 2.02 2.00 2.00 1.97 1.97 1.94 1.94 1.92 1.91 1.90
1H NMR (300.13 MHz, CDCl3)
(E)-3-((1-(phenyldiazenyl)cyclobutoxy)imino)pentane-2,4-dione, 3ce
Ph
N
N
O
N
O
O
Title i7358.{1H}.1.fid
Solvent CDCl3
Temperature 298.0
Pulse Sequence zg
Number of Scans 1
Receiver Gain 32.0
Relaxation Delay 0.1000
Pulse Width 14.0000
Acquisition Time 2.7724
Spectrometer Frequency 300.23
Spectral Width 5882.4
Lowest Frequency -1094.5
Nucleus 1H
Acquired Size 16308
Spectral Size 65536
2.00 3.01 4.00 3.00 3.04 1.98
11.5 11.0 10.5 10.0 9.5 9.0 8.5 8.0 7.5 7.0 6.5 6.0 5.5 5.0 4.5 4.0 3.5 3.0 2.5 2.0 1.5 1.0 0.5
ppm

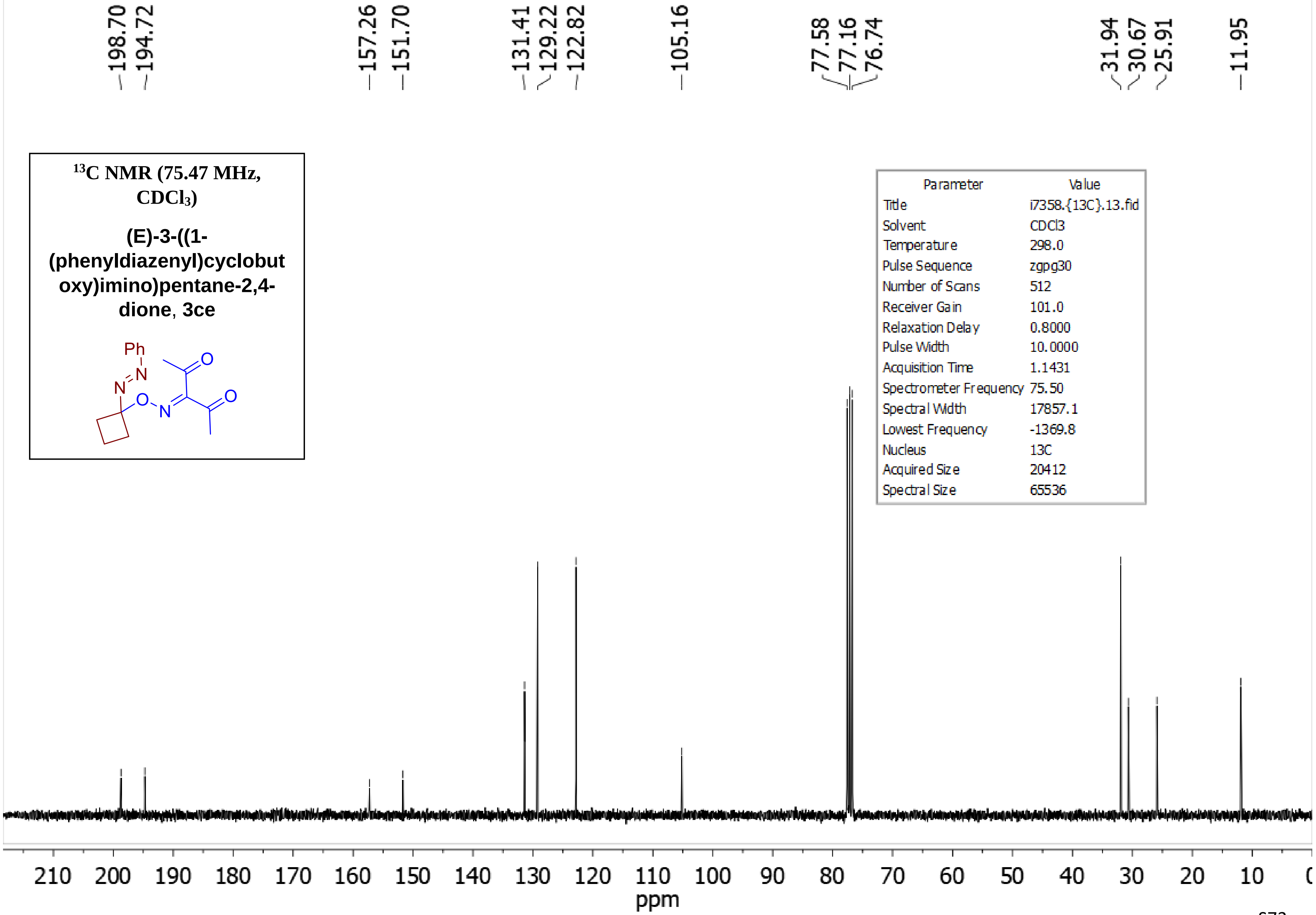

^{13}C NMR (75.47 MHz, $CDCl_3$)

(E)-3-((1-(phenyldiazenyl)cyclobutoxy)imino)pentane-2,4-dione, 3ce

Parameter	Value
Title	i7358.{13C}.13.fid
Solvent	CDCl3
Temperature	298.0
Pulse Sequence	zgpg30
Number of Scans	512
Receiver Gain	101.0
Relaxation Delay	0.8000
Pulse Width	10.0000
Acquisition Time	1.1431
Spectrometer Frequency	75.50
Spectral Width	17857.1
Lowest Frequency	-1369.8
Nucleus	13C
Acquired Size	20412
Spectral Size	65536

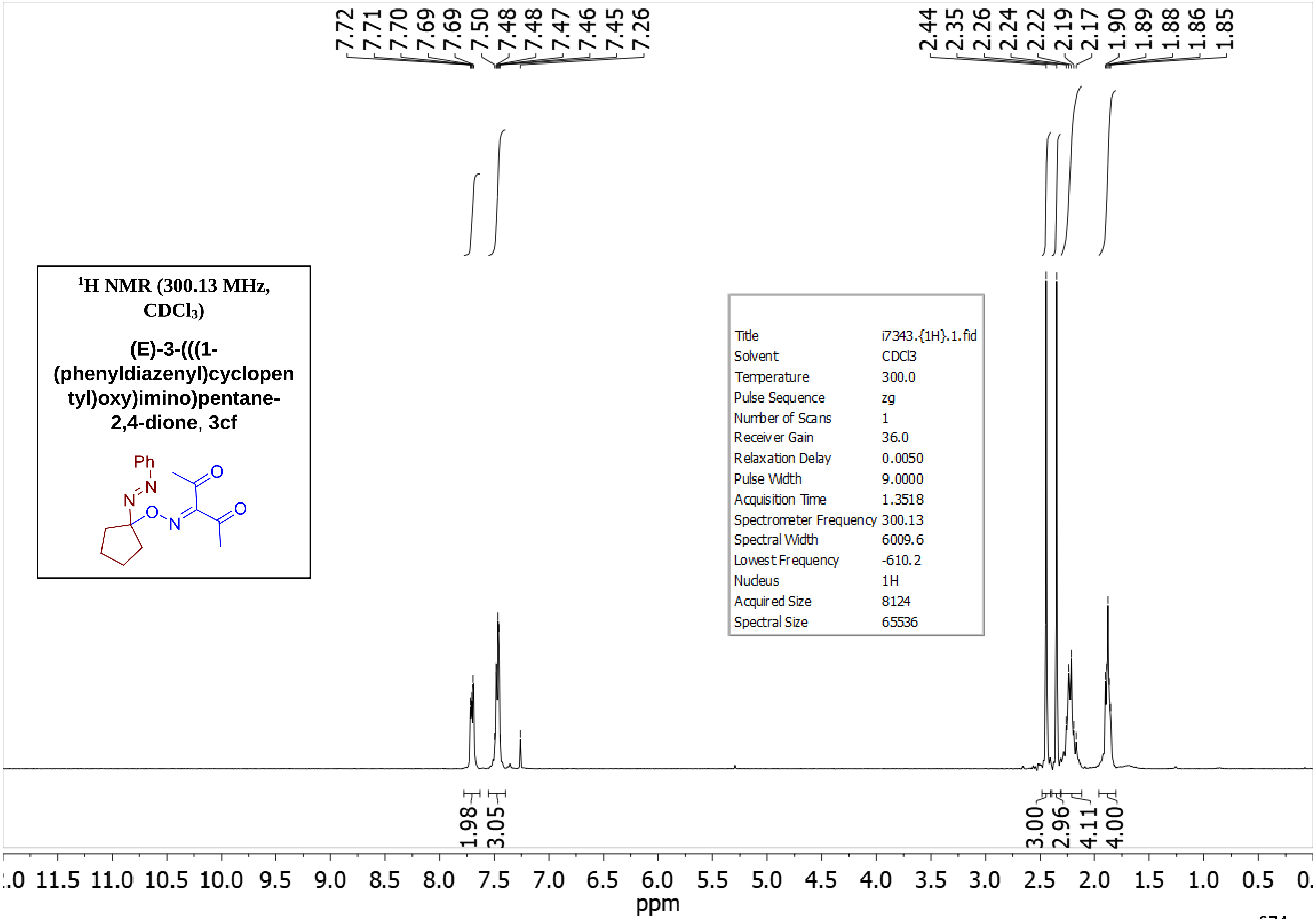

7.72
7.71
7.70
7.69
7.69
7.50
7.48
7.48
7.47
7.46
7.45
7.26
2.44
2.35
2.26
2.24
2.22
2.19
2.17
1.90
1.89
1.88
1.86
1.85
1H NMR (300.13 MHz, CDCl3)
(E)-3-(((1-(phenyldiazenyl)cyclopentyl)oxy)imino)pentane-2,4-dione, 3cf
Ph
N
N
O
N
O
O
Title i7343.{1H}.1.fid
Solvent CDCl3
Temperature 300.0
Pulse Sequence zg
Number of Scans 1
Receiver Gain 36.0
Relaxation Delay 0.0050
Pulse Width 9.0000
Acquisition Time 1.3518
Spectrometer Frequency 300.13
Spectral Width 6009.6
Lowest Frequency -610.2
Nucleus 1H
Acquired Size 8124
Spectral Size 65536
1.98
3.05
3.00
2.96
4.11
4.00
11.5 11.0 10.5 10.0 9.5 9.0 8.5 8.0 7.5 7.0 6.5 6.0 5.5 5.0 4.5 4.0 3.5 3.0 2.5 2.0 1.5 1.0 0.5
ppm

^{13}C NMR (75.47 MHz, $CDCl_3$)

(E)-3-(((1-(phenyldiazenyl)cyclopentyl)oxy)imino)pentane-2,4-dione, 3cf

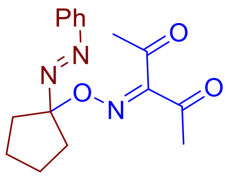

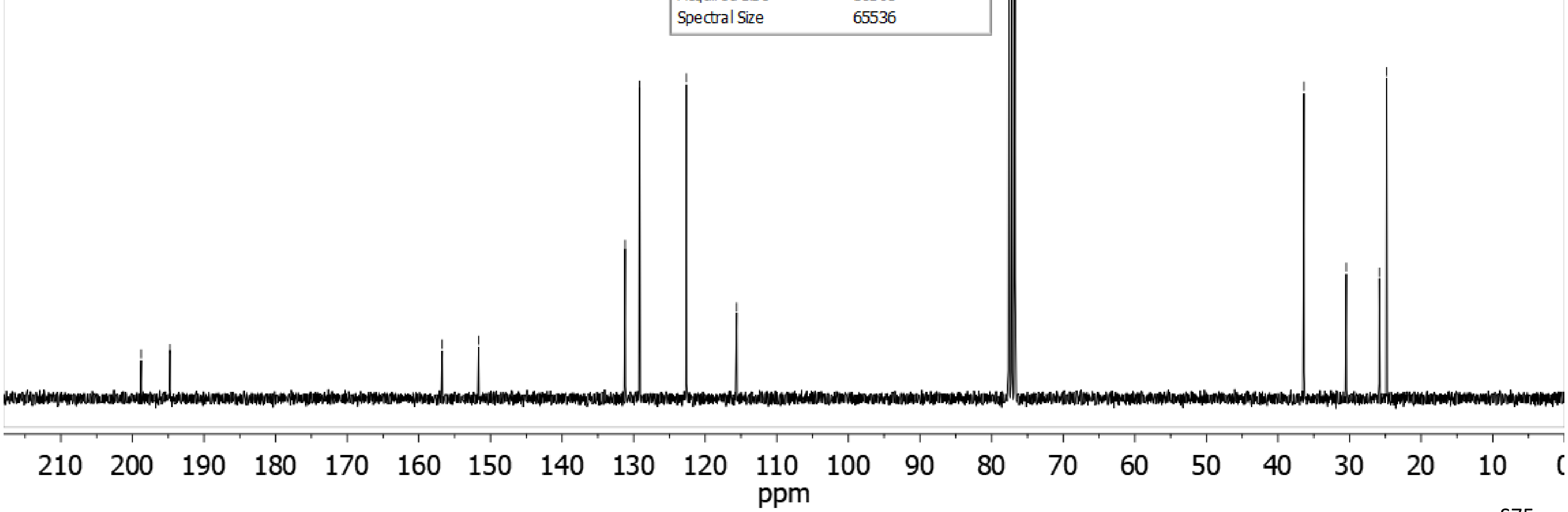

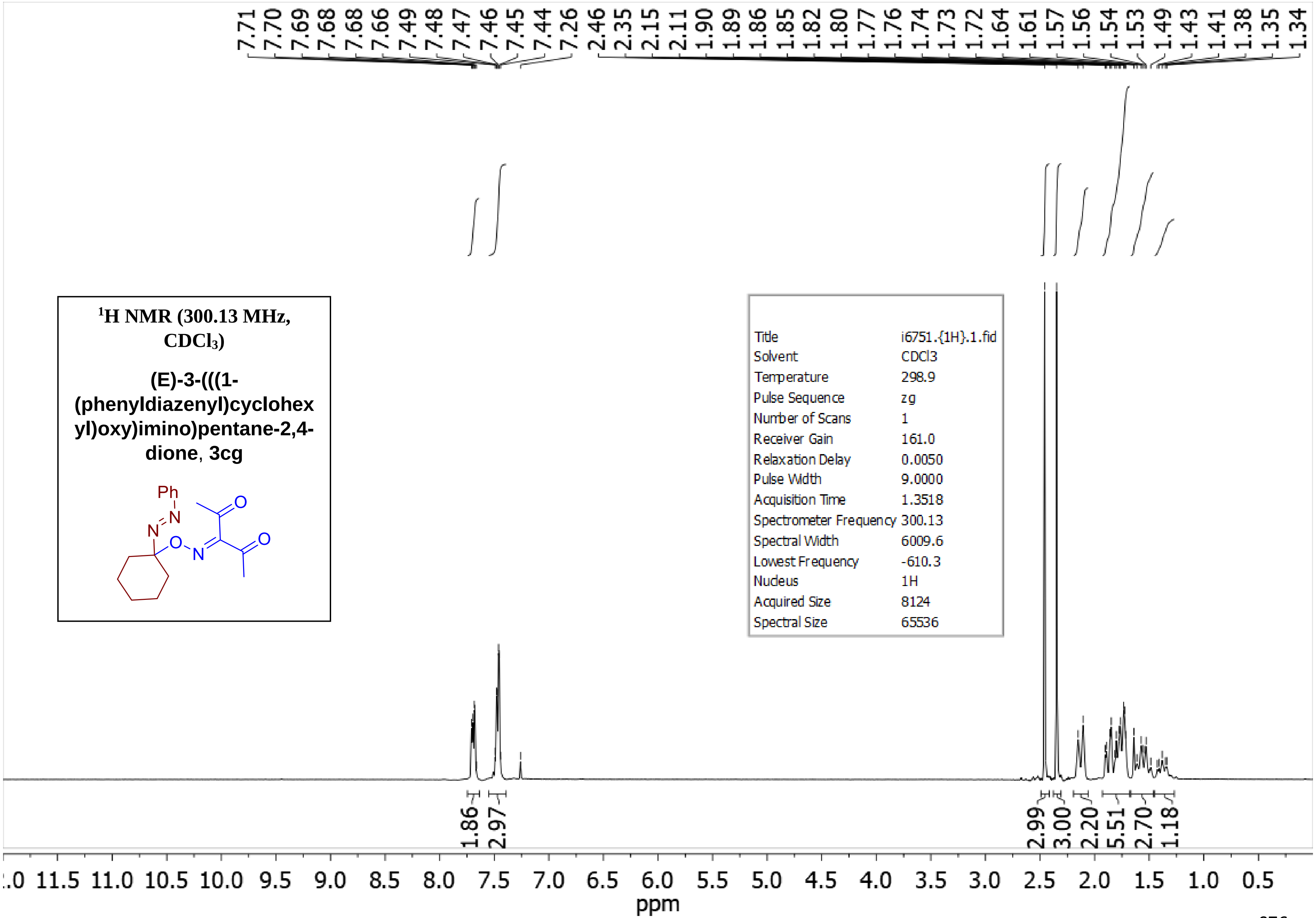
7.71 7.70 7.69 7.68 7.68 7.66 7.49 7.48 7.47 7.46 7.46 7.45 7.44 7.26 2.46 2.35 2.15 2.11 1.90 1.89 1.86 1.85 1.82 1.80 1.77 1.76 1.74 1.73 1.72 1.64 1.61 1.57 1.56 1.54 1.53 1.49 1.43 1.41 1.38 1.35 1.34
¹H NMR (300.13 MHz, CDCl3)
(E)-3-(((1-(phenyldiazenyl)cyclohexyl)oxy)imino)pentane-2,4-dione, 3cg
Ph
N
N
O
N
O
O
Title i6751.{1H}.1.fid
Solvent CDCl3
Temperature 298.9
Pulse Sequence zg
Number of Scans 1
Receiver Gain 161.0
Relaxation Delay 0.0050
Pulse Width 9.0000
Acquisition Time 1.3518
Spectrometer Frequency 300.13
Spectral Width 6009.6
Lowest Frequency -610.3
Nucleus 1H
Acquired Size 8124
Spectral Size 65536
1.86 2.97 2.99 3.00 2.20 5.51 2.70 1.18
.0 11.5 11.0 10.5 10.0 9.5 9.0 8.5 8.0 7.5 7.0 6.5 6.0 5.5 5.0 4.5 4.0 3.5 3.0 2.5 2.0 1.5 1.0 0.5
ppm

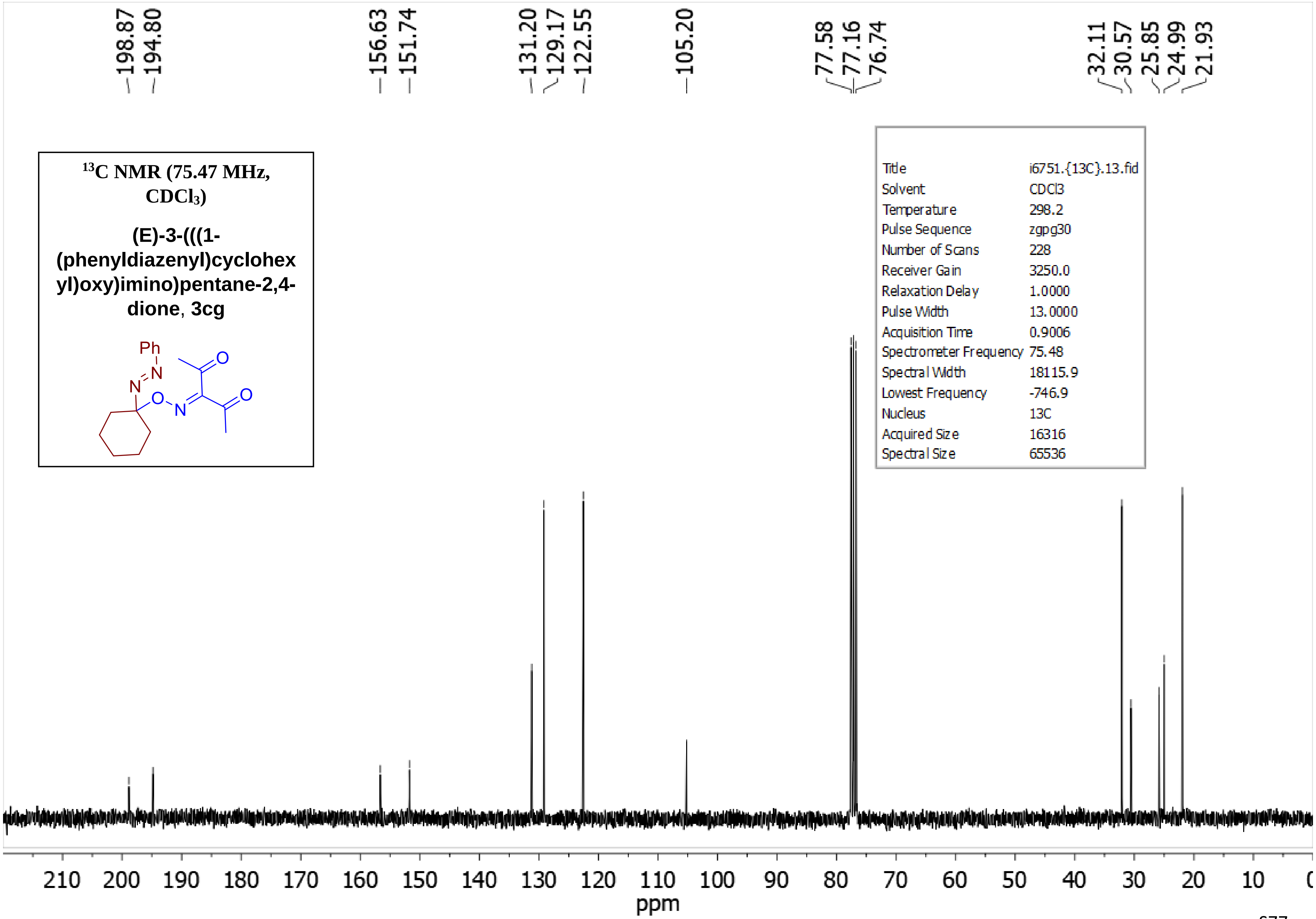
198.87
194.80
156.63
151.74
131.20
129.17
122.55
105.20
77.58
77.16
76.74
32.11
30.57
25.85
24.99
21.93
^{13}C NMR (75.47 MHz, $CDCl_3$)
(E)-3-(((1-(phenyldiazenyl)cyclohexyl)oxy)imino)pentane-2,4-dione, 3cg
Ph
N
N
O
N
O
O
Title i6751.{13C}.13.fid
Solvent CDCl3
Temperature 298.2
Pulse Sequence zgpg30
Number of Scans 228
Receiver Gain 3250.0
Relaxation Delay 1.0000
Pulse Width 13.0000
Acquisition Time 0.9006
Spectrometer Frequency 75.48
Spectral Width 18115.9
Lowest Frequency -746.9
Nucleus 13C
Acquired Size 16316
Spectral Size 65536
210 200 190 180 170 160 150 140 130 120 110 100 90 80 70 60 50 40 30 20 10
ppm

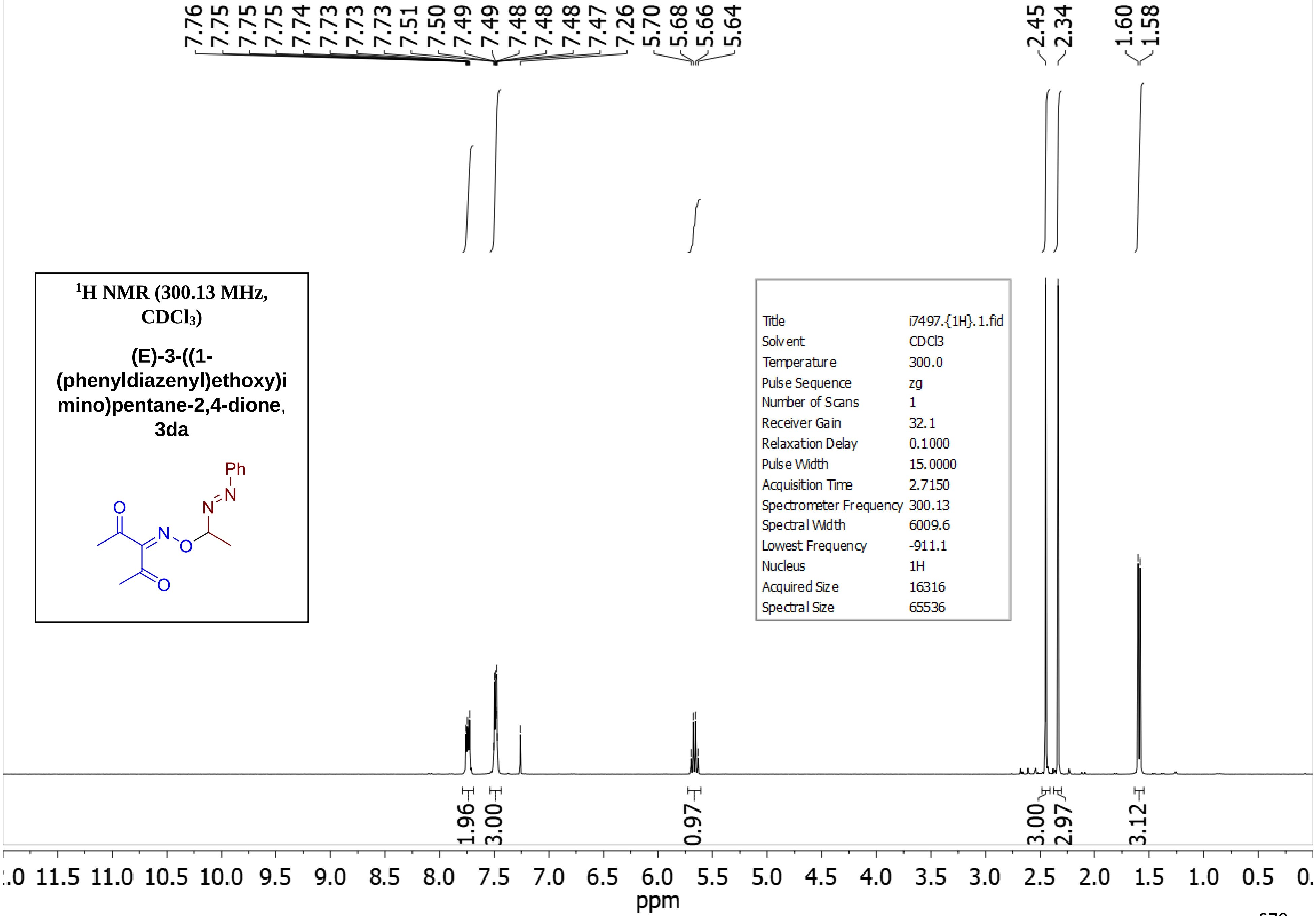

^{1}H NMR (300.13 MHz, $CDCl_3$)

(E)-3-((1-(phenyldiazenyl)ethoxy)imino)pentane-2,4-dione, 3da

Title	i7497.{1H}.1.fid
Solvent	CDCl3
Temperature	300.0
Pulse Sequence	zg
Number of Scans	1
Receiver Gain	32.1
Relaxation Delay	0.1000
Pulse Width	15.0000
Acquisition Time	2.7150
Spectrometer Frequency	300.13
Spectral Width	6009.6
Lowest Frequency	-911.1
Nucleus	1H
Acquired Size	16316
Spectral Size	65536

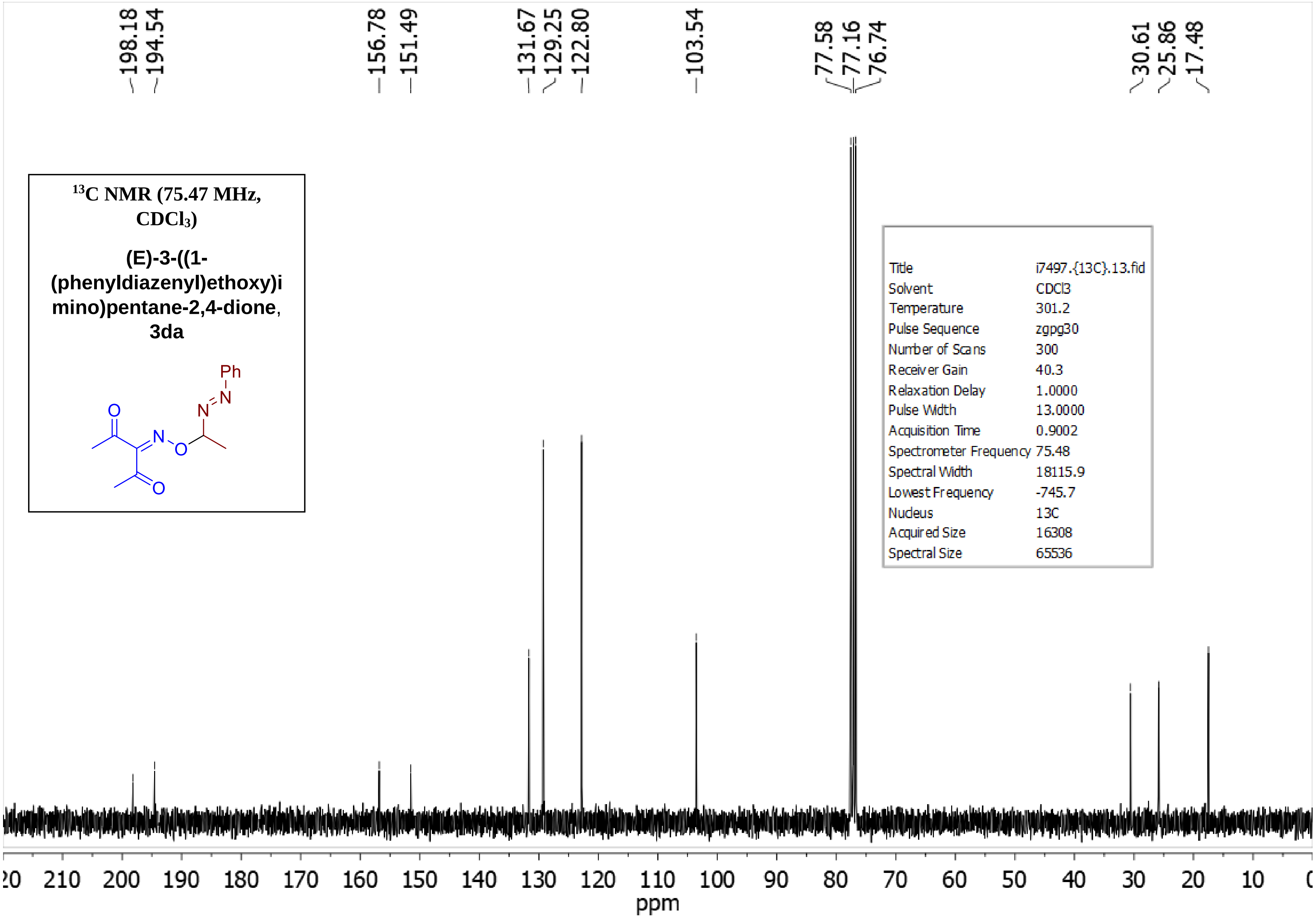
198.18
194.54
156.78
151.49
131.67
129.25
122.80
103.54
77.58
77.16
76.74
30.61
25.86
17.48
13C NMR (75.47 MHz, CDCl3)
(E)-3-((1-(phenyldiazenyl)ethoxy)imino)pentane-2,4-dione, 3da
Ph
N
N
O
N
O
O
Title i7497.{13C}.13.fid
Solvent CDCl3
Temperature 301.2
Pulse Sequence zgpg30
Number of Scans 300
Receiver Gain 40.3
Relaxation Delay 1.0000
Pulse Width 13.0000
Acquisition Time 0.9002
Spectrometer Frequency 75.48
Spectral Width 18115.9
Lowest Frequency -745.7
Nucleus 13C
Acquired Size 16308
Spectral Size 65536
210
200
190
180
170
160
150
140
130
120
110
100
90
80
70
60
50
40
30
20
10
ppm

^{1}H NMR (300.13 MHz, $CDCl_3$)

(E)-3-((1-(phenyldiazenyl)propoxy)imino)pentane-2,4-dione, 3db

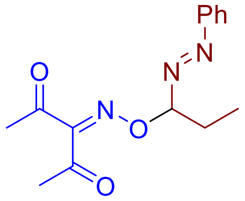

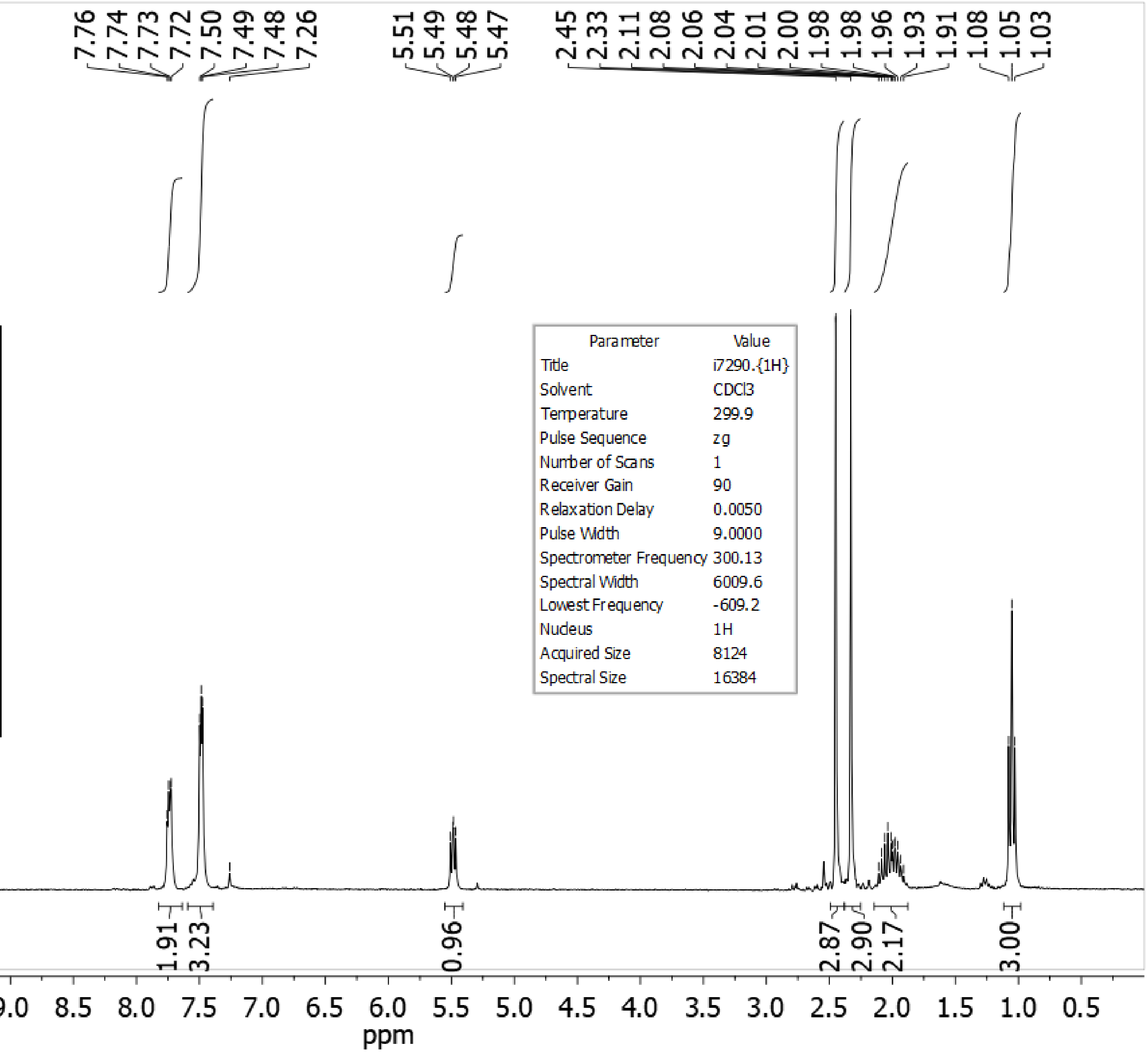

Parameter	Value
Title	i7290.{1H}
Solvent	CDCl3
Temperature	299.9
Pulse Sequence	zg
Number of Scans	1
Receiver Gain	90
Relaxation Delay	0.0050
Pulse Width	9.0000
Spectrometer Frequency	300.13
Spectral Width	6009.6
Lowest Frequency	-609.2
Nucleus	1H
Acquired Size	8124
Spectral Size	16384

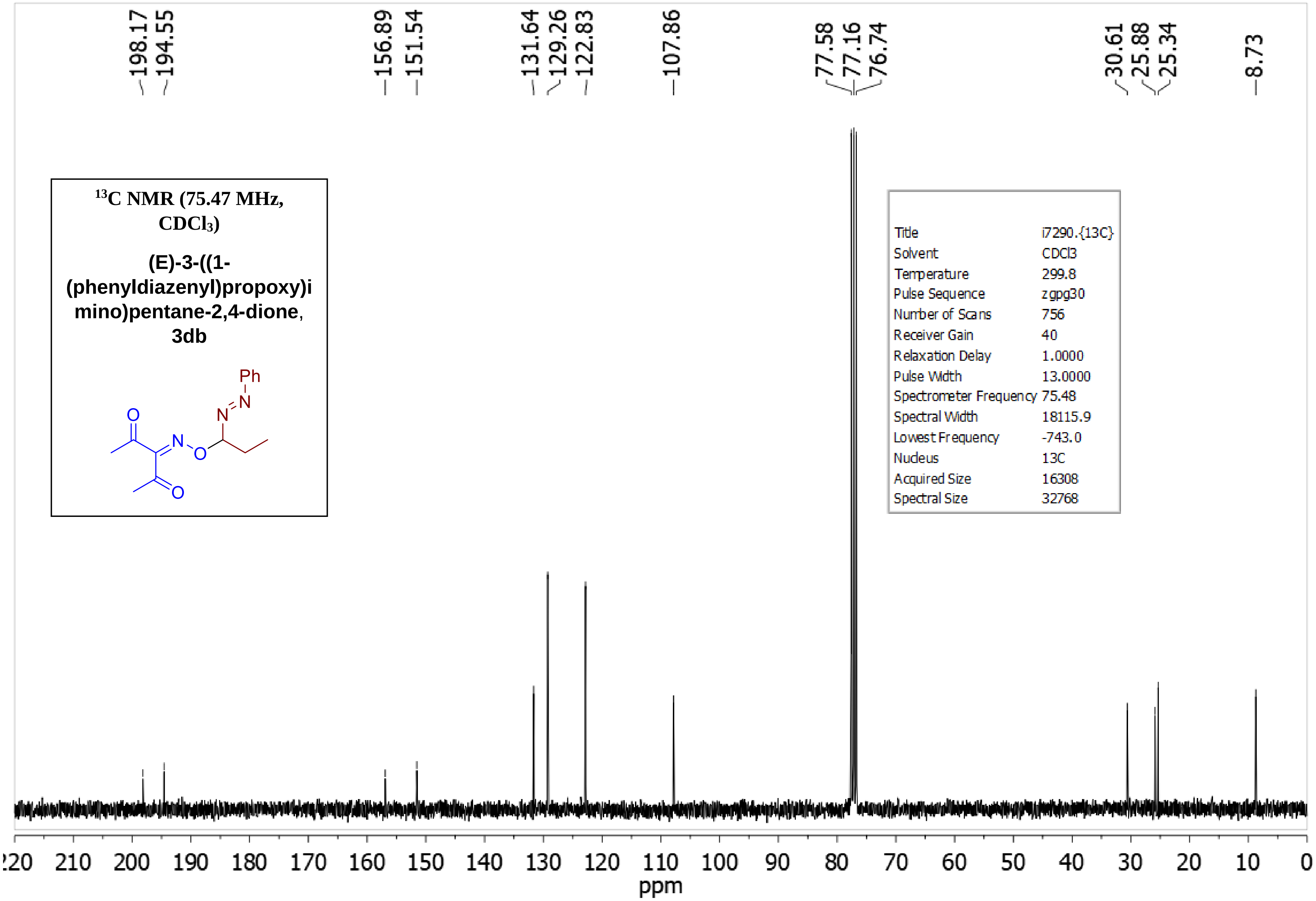
198.17
194.55
156.89
151.54
131.64
129.26
122.83
107.86
77.58
77.16
76.74
30.61
25.88
25.34
8.73
13C NMR (75.47 MHz, CDCl3)
(E)-3-((1-(phenyldiazenyl)propoxy)imino)pentane-2,4-dione, 3db
Ph
N
N
O
N
O
O
Title i7290.{13C}
Solvent CDCl3
Temperature 299.8
Pulse Sequence zgpg30
Number of Scans 756
Receiver Gain 40
Relaxation Delay 1.0000
Pulse Width 13.0000
Spectrometer Frequency 75.48
Spectral Width 18115.9
Lowest Frequency -743.0
Nucleus 13C
Acquired Size 16308
Spectral Size 32768
220 210 200 190 180 170 160 150 140 130 120 110 100 90 80 70 60 50 40 30 20 10 0
ppm

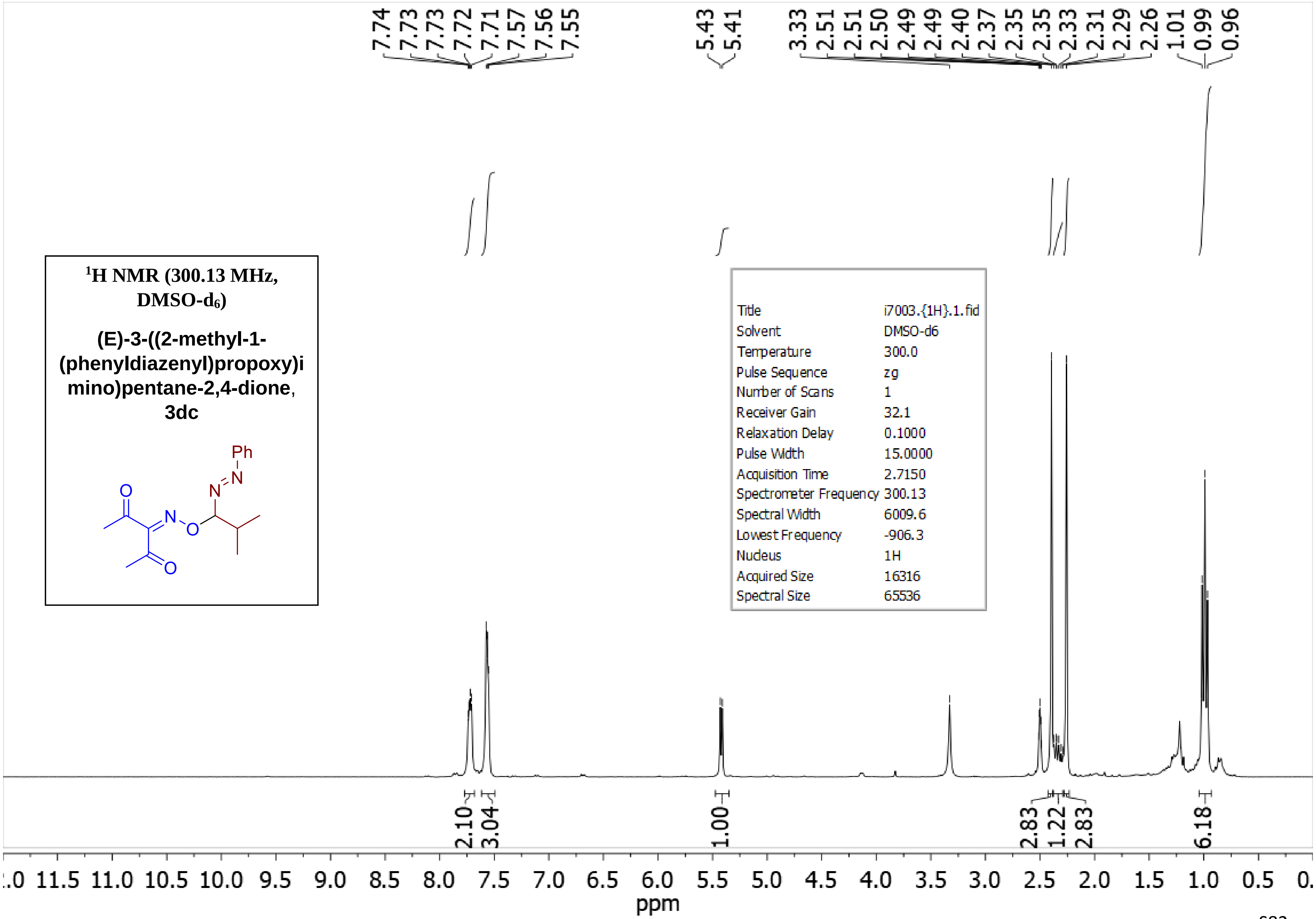

^{1}H NMR (300.13 MHz, DMSO-d6)
(E)-3-((2-methyl-1-(phenyldiazenyl)propoxy)imino)pentane-2,4-dione, 3dc
Ph
N
N
O
N
O
O
7.74
7.73
7.73
7.72
7.71
7.57
7.56
7.55
5.43
5.41
3.33
2.51
2.51
2.50
2.49
2.49
2.40
2.37
2.35
2.35
2.33
2.31
2.29
2.26
1.01
0.99
0.96
Title i7003.{1H}.1.fid
Solvent DMSO-d6
Temperature 300.0
Pulse Sequence zg
Number of Scans 1
Receiver Gain 32.1
Relaxation Delay 0.1000
Pulse Width 15.0000
Acquisition Time 2.7150
Spectrometer Frequency 300.13
Spectral Width 6009.6
Lowest Frequency -906.3
Nucleus 1H
Acquired Size 16316
Spectral Size 65536
2.10
3.04
1.00
2.83
1.22
2.83
6.18
ppm

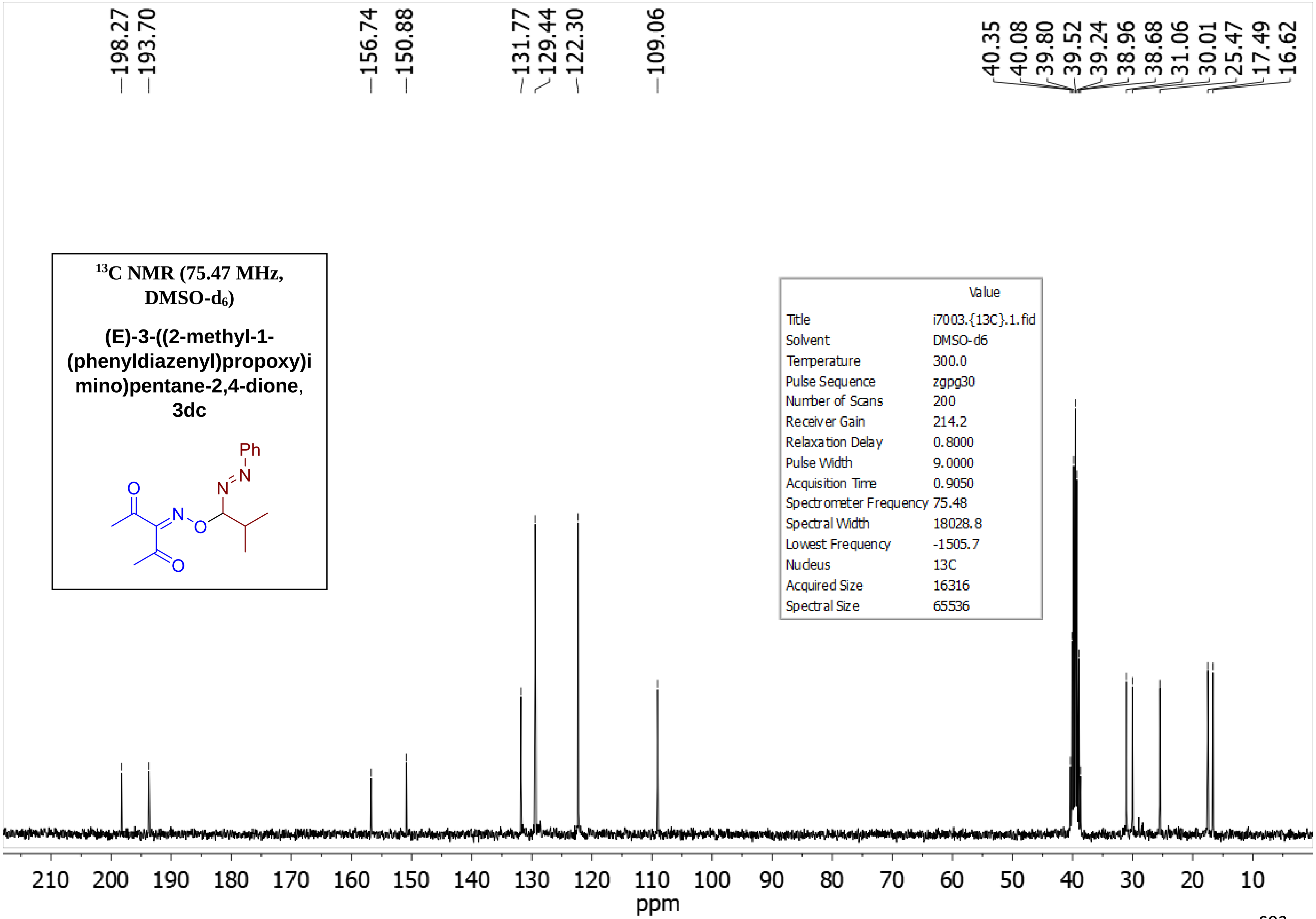
198.27
193.70
156.74
150.88
131.77
129.44
122.30
109.06
40.35
40.08
39.80
39.52
39.24
38.96
38.68
31.06
30.01
25.47
17.49
16.62
13C NMR (75.47 MHz, DMSO-d6)
(E)-3-((2-methyl-1-(phenyldiazenyl)propoxy)imino)pentane-2,4-dione, 3dc
Ph
N
N
O
N
O
O
Value
Title i7003.{13C}.1.fid
Solvent DMSO-d6
Temperature 300.0
Pulse Sequence zgpg30
Number of Scans 200
Receiver Gain 214.2
Relaxation Delay 0.8000
Pulse Width 9.0000
Acquisition Time 0.9050
Spectrometer Frequency 75.48
Spectral Width 18028.8
Lowest Frequency -1505.7
Nucleus 13C
Acquired Size 16316
Spectral Size 65536
210 200 190 180 170 160 150 140 130 120 110 100 90 80 70 60 50 40 30 20 10
ppm

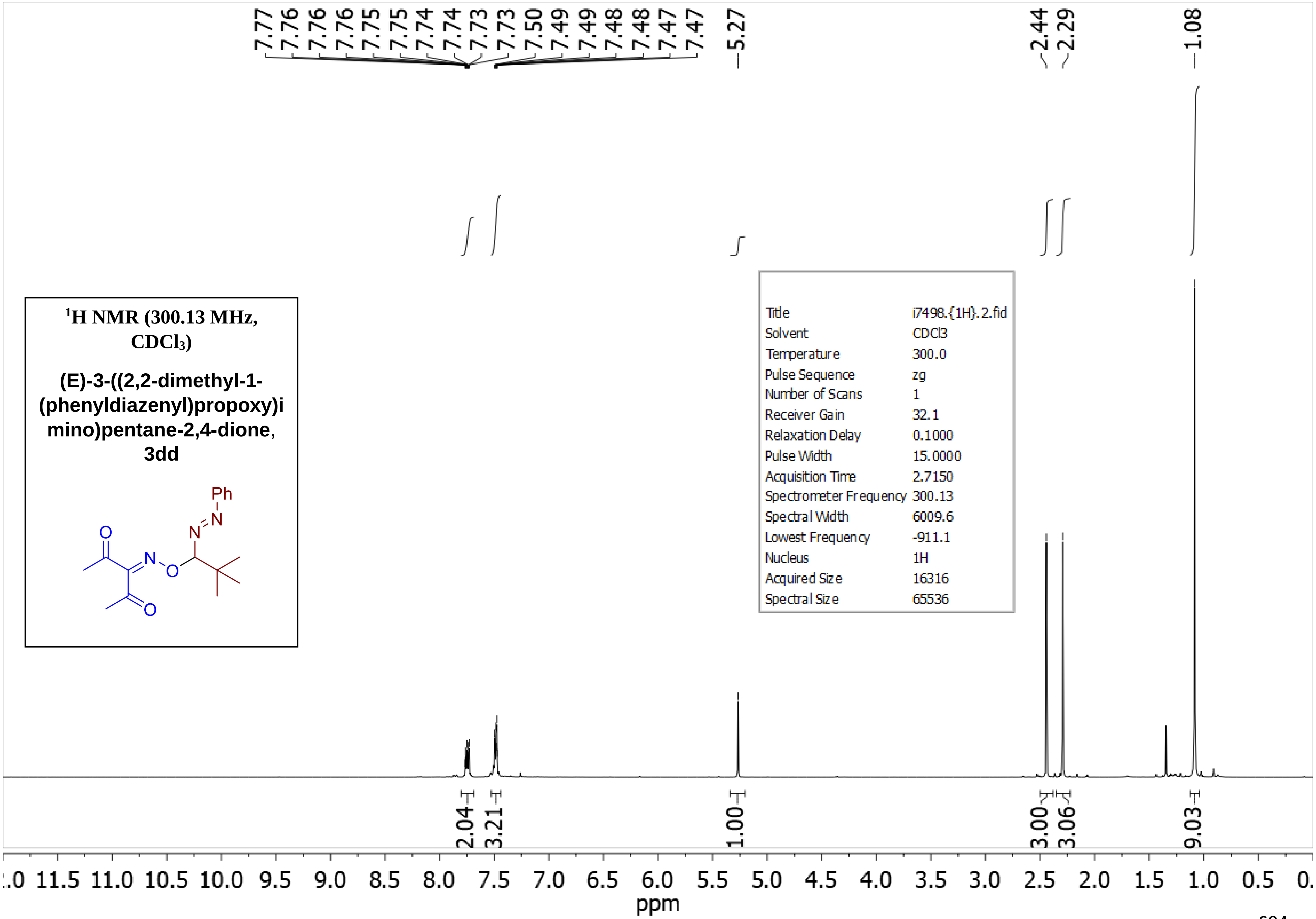

^{1}H NMR (300.13 MHz, $CDCl_3$)

(E)-3-((2,2-dimethyl-1-(phenyldiazenyl)propoxy)imino)pentane-2,4-dione, 3dd

^{13}C NMR (75.47 MHz, $CDCl_3$)

(E)-3-((2,2-dimethyl-1-(phenyldiazenyl)propoxy)imino)pentane-2,4-dione, 3dd

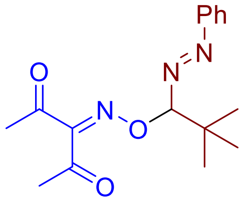

Title	i7498.{13C}.13.fid
Solvent	CDCl3
Temperature	301.9
Pulse Sequence	zgpg30
Number of Scans	1024
Receiver Gain	40.3
Relaxation Delay	1.0000
Pulse Width	13.0000
Acquisition Time	0.9002
Spectrometer Frequency	75.48
Spectral Width	18115.9
Lowest Frequency	-745.1
Nucleus	13C
Acquired Size	16308
Spectral Size	65536

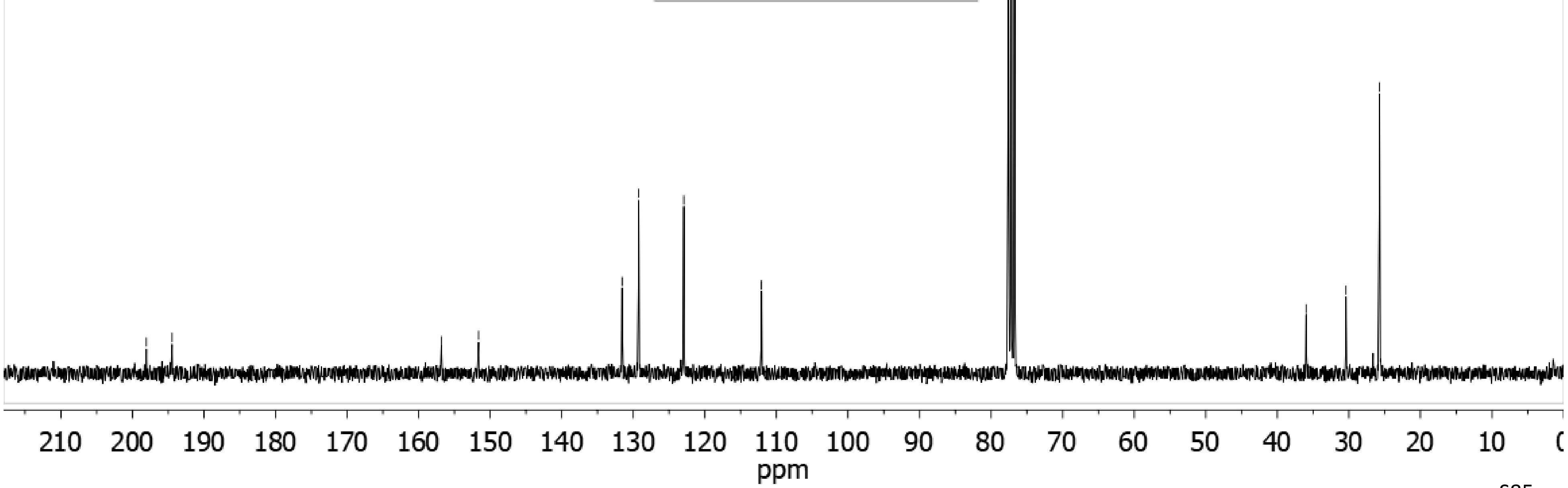

^{1}H NMR (300.13 MHz, $CDCl_3$)

(E)-3-(((1-(phenyldiazenyl)hexyl)oxy)imino)pentane-2,4-dione, 3de

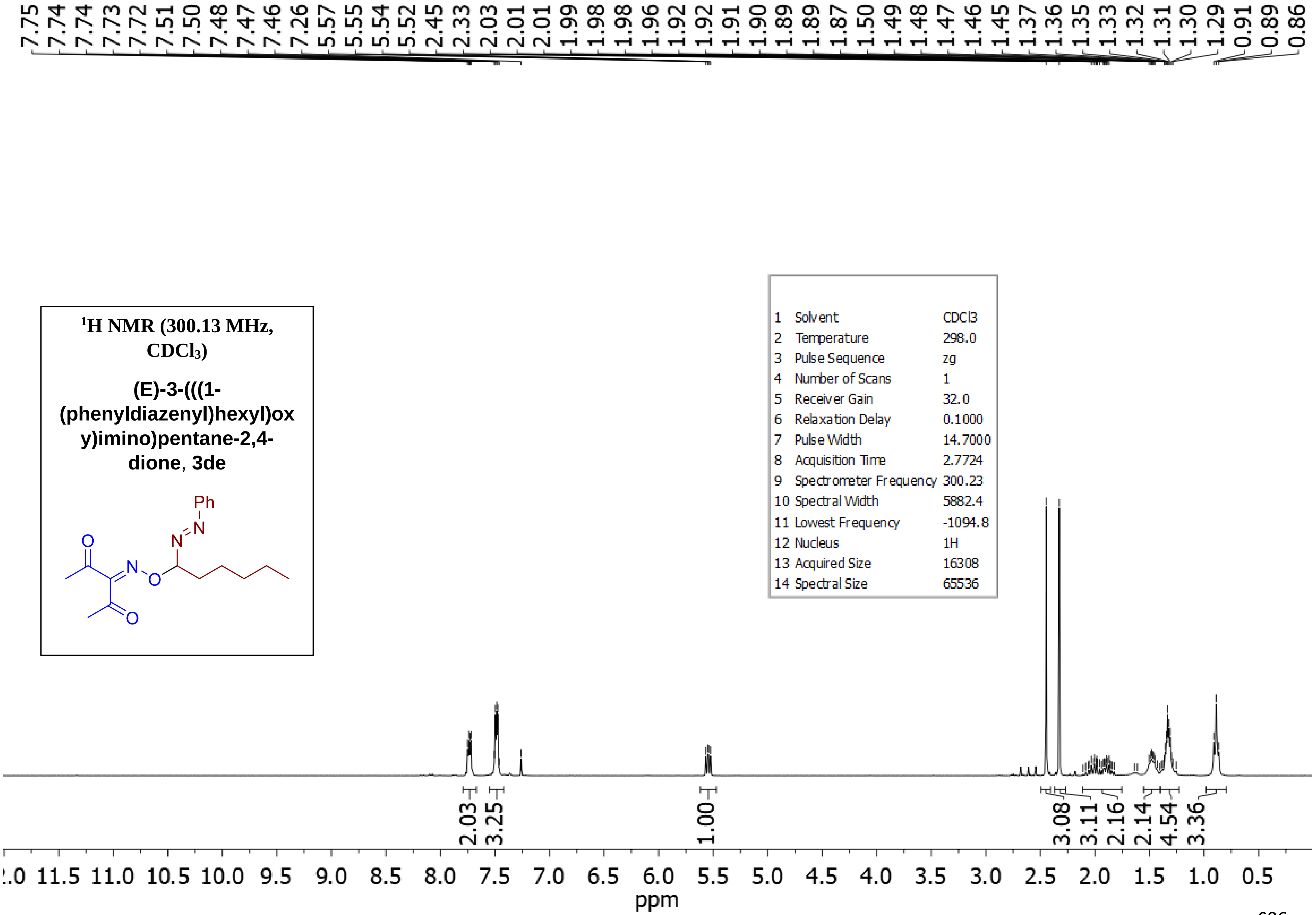

1	Solvent	CDCl3
2	Temperature	298.0
3	Pulse Sequence	zg
4	Number of Scans	1
5	Receiver Gain	32.0
6	Relaxation Delay	0.1000
7	Pulse Width	14.7000
8	Acquisition Time	2.7724
9	Spectrometer Frequency	300.23
10	Spectral Width	5882.4
11	Lowest Frequency	-1094.8
12	Nucleus	1H
13	Acquired Size	16308
14	Spectral Size	65536

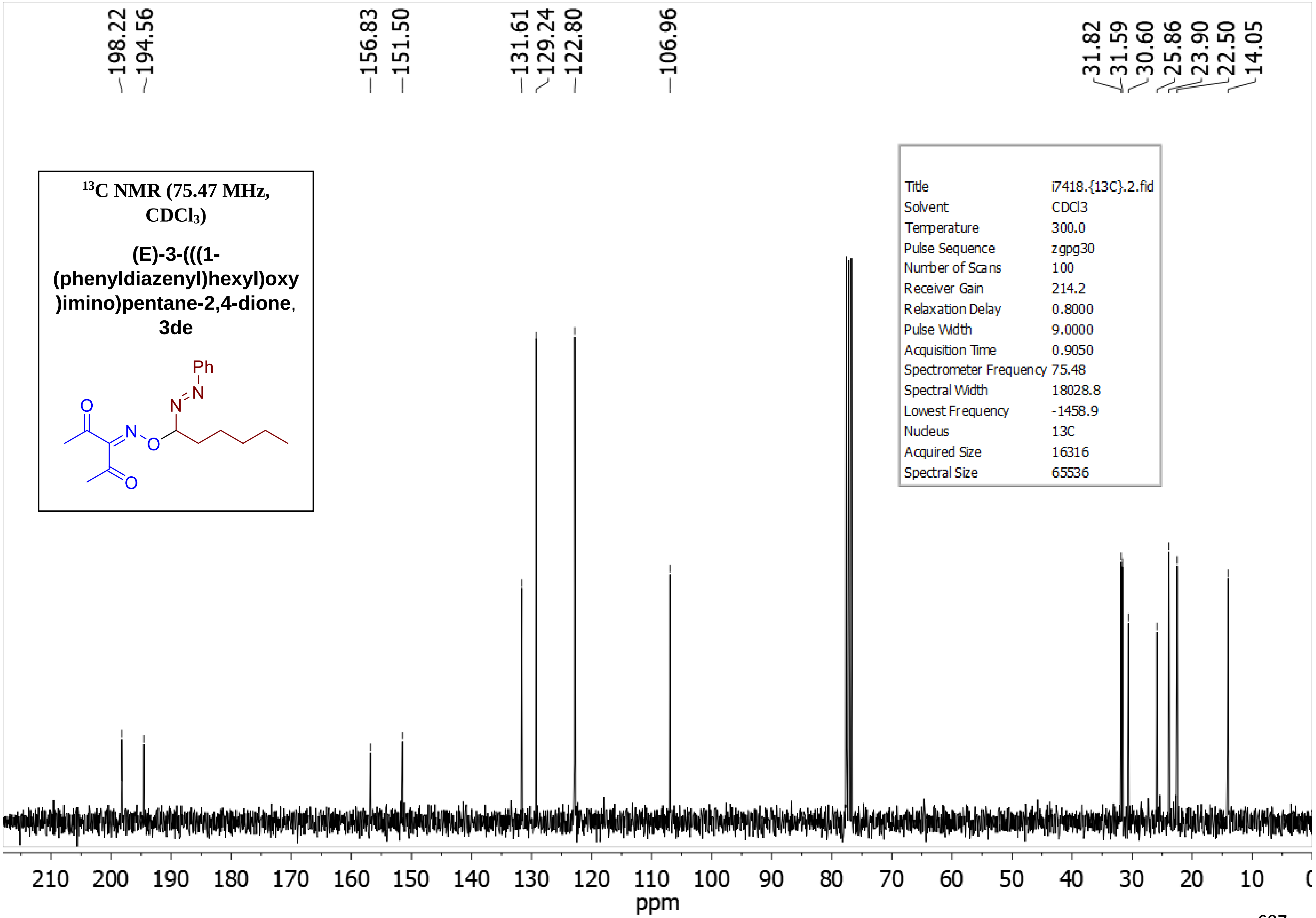
198.22
194.56
156.83
151.50
131.61
129.24
122.80
106.96
31.82
31.59
30.60
25.86
23.90
22.50
14.05
13C NMR (75.47 MHz, CDCl3)
(E)-3-(((1-(phenyldiazenyl)hexyl)oxy)imino)pentane-2,4-dione, 3de
Ph
N
N
O
N
O
O
Title i7418.{13C}.2.fid
Solvent CDCl3
Temperature 300.0
Pulse Sequence zgpg30
Number of Scans 100
Receiver Gain 214.2
Relaxation Delay 0.8000
Pulse Width 9.0000
Acquisition Time 0.9050
Spectrometer Frequency 75.48
Spectral Width 18028.8
Lowest Frequency -1458.9
Nucleus 13C
Acquired Size 16316
Spectral Size 65536
210 200 190 180 170 160 150 140 130 120 110 100 90 80 70 60 50 40 30 20 10
ppm
